# Self-powered mechanoluminescent elastomer for solar-blind ultraviolet emission

**DOI:** 10.1038/s41377-025-02131-2

**Published:** 2026-01-12

**Authors:** Xulong Lv, Tianyi Duan, Shaofan Fang, Zhaofeng Wang, Dongxun Chen, Lipeng Huang, Huanyu Liu, Zheming Liu, Chao Liu, Xiao-Jun Wang, Yanjie Liang

**Affiliations:** 1 School of Materials Science & Engineering, Shandong University, Jinan, 250061 China; 2 State Key Laboratory of Coatings for Advanced Equipment, Shandong University, Jinan, 250061 China; 3 School of Information and Communication Engineering, Beijing University of Posts and Telecommunications, Beijing, 100876 China; 4 Shandong Laboratory of Advanced Materials and Green Manufacturing at Yantai, Yantai, 264006 China; 5 State Key Laboratory of Solid Lubrication, Lanzhou Institute of Chemical Physics, Chinese Academy of Sciences, Lanzhou, 730000 China; 6 Department of Oral and Maxillofacial Surgery, School and Hospital of Stomatology, Cheeloo College of Medicine, Shandong University, Jinan, 250012 China; 7 Department of Oral and Maxillofacial Surgery, Qilu Hospital of Shandong University, Jinan, 250012 China; 8 Department of Physics, Georgia Southern University, Statesboro, GA 30460 USA

**Keywords:** Optical materials and structures, Electronics, photonics and device physics, Lasers, LEDs and light sources

## Abstract

Flexible mechanoluminescence (ML) elastomers show significant potential for next-generation wearable electronics, artificial skin, advanced sensing, and human-machine interaction. However, their broader application has been hindered by challenges such as restricted emission wavelengths, inadequate repeatability, insufficient cyclic stability, and poor self-recovery. Here, we report an innovative and high-performance solar-blind ultraviolet ML elastomer by combining commercial polydimethylsiloxane (PDMS) and newly fabricated Sr_3_(BO_3_)_2_:Pr^3+^ phosphors, capable of generating intense ultraviolet-C (UVC) ML peaked at 272 nm under mechanical stimulation. The composite elastomer exhibits exceptional repeatability and cyclic stability, maintaining detectable UVC emission over 10,000 continuous stretching cycles (power intensity at the 1st cycle is ~6.2 mW m^−2^). It also demonstrates rapid and efficient self-recovery behavior, restoring 43.2% of its initial intensity within 1 s and 90.2% after 24 h. Combined experimental and theoretical analyses reveal that interfacial triboelectrification, involving electron transfer from the phosphor to the PDMS matrix, leads to the observed UVC ML emission. Leveraging the solar-blind nature and high photon energy of UVC light, we further demonstrate the feasibility of self-powered photonics applications. This work not only offers novel insights into the design of advanced UVC ML systems but also provides “power-free” solutions for important applications where UVC photons are essential, such as outdoor optical tagging and microbial sterilization.

## Abstract (web summary)

We developed a robust self-powered and self-recovered solar-blind UV ML elastomer composed of commercial polydimethylsiloxane and newly synthesized Sr_3_(BO_3_)_2_:Pr^3+^ phosphors. The emerging applications of this UVC ML elastomer in covert optical tagging and microbial sterilization were demonstrated.

## Graphical abstract

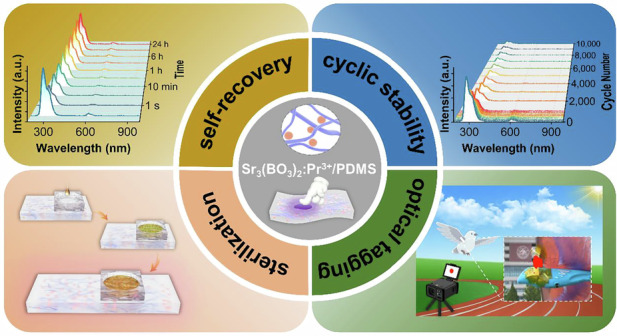

## Introduction

Mechanoluminescence (ML) is the emission of light from materials in response to various mechanical stimuli such as fracture, friction, compression, grinding, and stretching^1–5^. This unique mechanics-to-optics conversion phenomenon has attracted significant research interest since its first reported observation in 1605^6–8^. ML research has evolved from studies of destructive luminescence to the development of repeatable and elastic material systems, spanning a wide range of inorganic and organic compounds^9–16^. Compared to rigid inorganic phosphors and unstable organic polymers, self-powered and self-recovered inorganic–organic ML elastomers, in which luminescent phosphors are embedded within a polymer matrix, offer notable advantages, including facile fabrication, high flexibility, and excellent physical/chemical stability^16–20^. These attributes position them as promising candidates for next-generation wearable electronics, artificial skin, advanced sensing, and human-machine interaction^21–24^.

Over recent decades, various ML elastomer systems have been developed^25–28^. Among them, ZnS:Cu/PDMS stands out as the most extensively studied, exhibiting bright, durable green ML emission over 10,000 mechanical cycles^29–32^. However, the intrinsic instability and limited emission range of sulfide-based systems have motivated exploration of novel ML elastomers with broader emission wavelengths and enhanced repeatability, cyclic stability, and self-recovery^33–45^. Despite significant advancements, the development and application of flexible ML elastomer systems face two major challenges. First, most ML elastomer systems emit within the visible to near-infrared (NIR) spectral region, limiting their applications to dark environments^37,46–50^, as the visible or NIR ML signals are submerged by the overwhelming background signal in bright environments (sunlight and indoor illumination). In contrast, ultraviolet (UV) ML elastomers, particularly those emitting in the shorter-wavelength ultraviolet-C (UVC) spectral region (200–280 nm), remain scarcely explored^36,38,51^. To date, UVC ML emission has only been observed in a very small number of trap-controlled systems using pre-irradiated phosphors such as Sr_2_P_2_O_7_:Pr^3+^ and SrF_2_:Pr^3+^^52,53^. Given the unique advantages of UVC light, such as solar-blind nature and high photon energy, there is growing interest in developing self-powered UVC ML elastomer systems for interference-free operation in bright environments^54–56^. The second challenge lies in achieving adequate repeatability, sufficient cyclic stability, and rapid self-recovery in ML elastomer systems. While ZnS:Cu/PDMS elastomer demonstrates excellent repeatability and cyclic stability via its electroluminescence mechanism^57,58^. This is not generally replicable in most other elastomers, where ML is driven by interfacial triboelectric effects and tends to degrade rapidly within limited cycles^25,45^. Recent studies highlight that robust interfacial interactions can significantly improve ML durability performance, as demonstrated in MPX:Eu/PDMS (M = Ca/Sr/Ba, X = Cl)^41^ and CaBa_4_(PO_4_)_3_Cl:Eu/PDMS^44^ systems, which sustain thousands of cycles with stable visible ML output. Thus, engineering robust phosphor–polymer interfaces is essential for developing UVC ML composite elastomers with outstanding durability and self-recovery capabilities.

In this work, we develop a robust self-powered and self-recovered solar-blind UV ML elastomer composed of commercial polydimethylsiloxane (PDMS) and newly synthesized Sr_3_(BO_3_)_2_:Pr^3+^ (SBO:Pr) phosphors. The SBO:Pr/PDMS composite elastomer exhibits broadband UVC ML emission centered at 272 nm under various mechanical stimuli, along with outstanding repeatability, cyclic stability, and self-recovery. The influence of stretching strain on ML cyclic stability and self-recovery performances was systematically investigated. Comprehensive experimental and theoretical analyses were conducted to elucidate the underlying UVC ML mechanisms. The practical potential of this elastomer system was further demonstrated in self-powered optical tagging and microbial sterilization applications.

## Results

### UVC photoluminescence

The crystal structure, X-ray diffraction (XRD) patterns, scanning electron microscope (SEM) images, energy-dispersive spectroscope (EDS) analysis, X-ray photoelectron spectroscopy (XPS) results, and the inductively coupled plasma mass spectrometry (ICP-MS) analysis of the newly synthesized SBO:Pr phosphors are presented in Figs. S1–S4 and Table S1. The XRD results confirm that the SBO:Pr phosphors possess a single-phase structure. Moreover, the successful incorporation of Pr^3+^ ions into the host lattice is evident, and EDS mapping reveals that all constituent elements are uniformly distributed within a randomly selected particle.

**Fig. 1:**
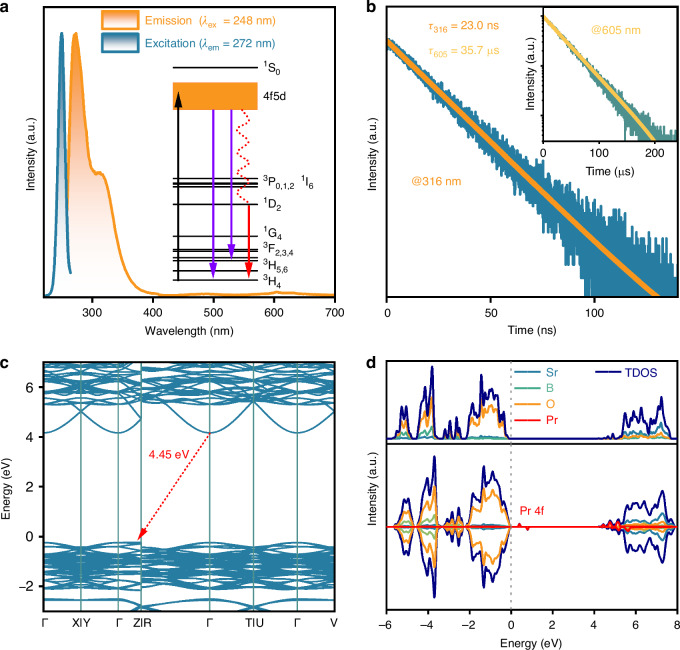
**UVC photoluminescence**. **a** The normalized photoluminescence emission and excitation spectra of the SBO:Pr phosphor at room temperature. Inset is the energy level diagram of the Pr^3+^ ion. The excitation spectrum is monitored at 272 nm, and the emission spectrum is obtained upon 248 nm excitation. **b** The luminescence decay curve and corresponding single exponential fitting curve of the SBO:Pr phosphor monitored at 316 nm. The inset shows the 605 nm emission decay. **c** The electronic band structure of the Sr_3_(BO_3_)_2_ host. **d** The densities of states (DOS) of the Sr_3_(BO_3_)_2_ host and the SBO:Pr phosphor

Figure 1a shows the normalized photoluminescence (PL) emission and excitation spectra of the SBO:Pr phosphor at room temperature, with an inset depicting the energy level diagram of the Pr^3+^ ion. The excitation spectrum monitored at 272 nm emission consists of a broad excitation band ranging from approximately 230 to 265 nm, with a maximum at 248 nm, which can be assigned to the 4f^2^→4f5d excitation transition of Pr^3+^. Under 248 nm excitation, the phosphor exhibits a strong broadband emission centered at 272 nm, accompanied by a weaker shoulder band at approximately 316 nm, both attributed to the Pr^3+^ 4f5d→4f^2^ transitions. Additionally, weak emission peaks are observed in the visible spectral region, corresponding to the intra-configurational 4f^2^→4f^2^ transitions. Figure S5 presents the PL emission spectra of SBO:*x*Pr phosphors, which exhibit identical spectral profiles apart from intensity differences. Figure 1b depicts the luminescence decay curves and the corresponding single exponential fitting curve of the SBO:Pr phosphor monitored at 316 and 605 nm, respectively. The 316 nm emission has a short decay time of 23.0 ns. In contrast, the emission at 605 nm that belongs to the parity-forbidden 4f^2^→4f^2^ transitions displays a much longer lifetime of 35.7 μs, approximately three orders of magnitude longer than that of the allowed 4f5d→4f^2^ transitions, highlighting the distinct radiative processes involved. Furthermore, the photoluminescence quantum yield (PLQY) of the SBO:Pr phosphor upon 250 nm excitation is determined to be 25.1% (Fig. S6).

Given that the densities of states (DOS) and electronic band structure of the host material are closely related to the luminescence properties of phosphors, the predicted electronic band structure and DOS are presented in Fig. 1c, d. The Sr_3_(BO_3_)_2_ host exhibits an indirect bandgap (Eg) with a predicted value of approximately 4.45 eV, demonstrating that it is suitable to accommodate Pr^3+^ ions and can provide a sufficiently wide energy gap to support UVC emission. Figs. 1d and S7 display the DOS of the Sr_3_(BO_3_)_2_ host and SBO:Pr phosphor with the Fermi energy level set to 0 eV. It is found that the conduction band (CB) is primarily derived from the d orbital of Sr atoms, while the valence band (VB) is mainly composed of the p orbitals of O atoms. Compared with the pure Sr_3_(BO_3_)_2_ host, the introduction of Pr^3+^ induces localized electronic states within the bandgap region, primarily originating from the 4f orbitals of Pr atoms. These new states potentially reduce the energy barrier for electron excitation and increase the available density of states near the VB, thereby enhancing the probability of electron transitions and contributing to improved luminescence performance.

### Self-powered and self-recovered UVC ML

**Fig. 2:**
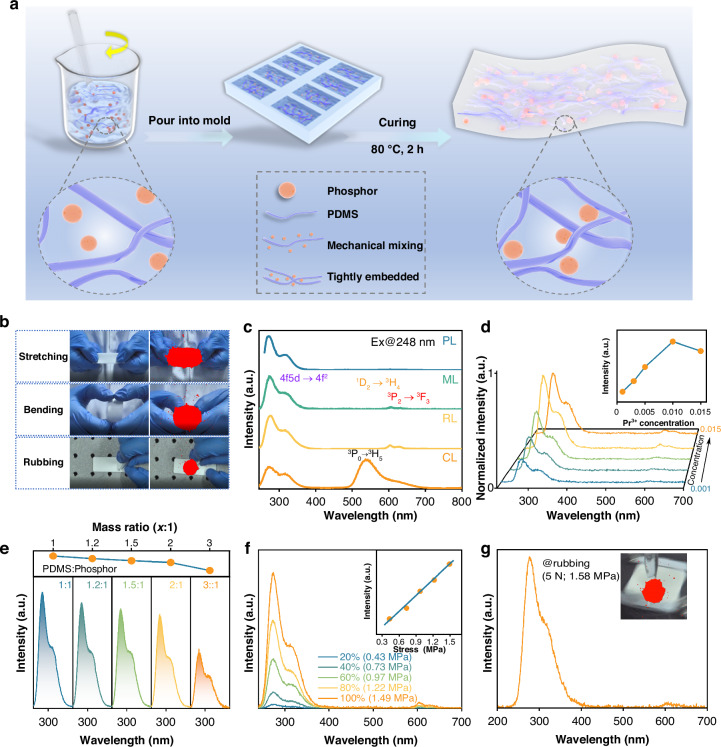
**Self-powered and self-recovered UVC ML**. **a** Schematic illustration of the fabrication process for the SBO:Pr/PDMS elastomer film. **b** UVC ML images of the elastomer film under various mechanical stimuli, including stretching, bending, and rubbing with a glass rod. **c** Comparison of ML, PL, RL, and CL emission spectra of the elastomer film. **d** ML emission spectra of SBO:Pr/PDMS elastomer films with varying Pr^3+^ doping concentrations under stretching. **e** ML emission spectra of elastomer films with different mass ratios of SBO:Pr phosphor to PDMS. The upper panel shows the corresponding variation in ML intensity as a function of mass ratio. **f** ML emission spectra of the elastomer film under different stretching strains. The inset shows a linear relationship between ML intensity and applied stress. **g** ML emission spectrum of the elastomer film under a frictional force of 5 N (stress:1.58 MPa). The inset displays the corresponding UVC ML image

Apart from the UVC PL, the SBO:Pr/PDMS composite elastomer composed of commercial polydimethylsiloxane (PDMS) and newly synthesized SBO:Pr phosphors exhibits robust self-powered and self-recovered UVC ML in response to mechanical stimuli without the need for pre-irradiation. Notably, the pristine SBO:Pr phosphor alone does not emit ML under direct mechanical stimulation, indicating that the UVC ML generation critically depends on interfacial interactions between the SBO:Pr phosphors and the PDMS polymer chains. The fabrication process of the SBO:Pr/PDMS elastomer is schematically illustrated in Fig. 2a. Upon curing, the SBO:Pr microcrystals are uniformly encapsulated and tightly bound by PDMS macromolecular chains, ensuring strong interfacial interactions between them. This robust interface facilitates interfacial contact-induced, self-recovered UVC ML when subjected to mechanical stimuli. Cross-sectional scanning electron microscope (SEM) images of elastomer film confirm the uniform distribution of SBO:Pr microcrystals throughout the PDMS matrix (Fig. S8).

Owing to the elasticity of the composite elastomer, the SBO:Pr/PDMS film exhibits distinct UVC emission in response to various mechanical stimuli, including stretching, bending, and rubbing. These emissions are clearly captured by a solar-blind UV camera, as shown in Fig. 2b and Video S1. Each image was obtained by overlaying the UVC image onto the corresponding visible image, where the red regions represent UVC luminescence. Figure 2c presents the UVC ML emission spectrum of the SBO:Pr/PDMS elastomer film under stretching stimulus, and the schematic diagram of the stretching device is shown in Fig. S9. Notably, the film exhibits intense UVC ML peaking at 272 nm without the need for pre-irradiation, corresponding to the 4f5d→4f^2^ transitions of Pr^3+^. This emission closely matches the PL and radioluminescence (RL) emission spectra, indicating that ML, PL, and RL originate from the same Pr^3+^ emitting centers. In contrast, the ML spectrum is markedly different from the cathodoluminescence (CL) spectrum that is dominated by the intense ^3^P_0_→^3^H_5_ transitions (Figs. S10–S12). This disparity confirms that ML is not induced by high-energy electron bombardment that is typically responsible for the CL^44,59^, but instead arises from a distinct excitation mechanism.

Figure 2d shows the ML emission spectra of SBO:Pr/PDMS elastomer film with varying Pr^3+^ doping concentrations under stretching. Altering the Pr^3+^ concentration from 0.001 to 0.015 does not affect the positions of its characteristic emission peaks, but only the ML intensity. As the Pr^3+^ concentration increases, the ML intensity initially rises, reaching a maximum at 0.010, and subsequently decreases at higher concentrations due to concentration quenching^60^. Furthermore, the influence of phosphor loading on ML performance was investigated by varying the mass ratio of SBO:Pr phosphor within the PDMS matrix, as shown in Fig. 2e. Increasing the phosphor content enhances ML intensity; however, it also significantly reduces the elasticity of the film (Fig. S13). A mass ratio of 1:1.5 (SBO:Pr to PDMS) was selected as optimal, offering a balance between high ML performance and desirable mechanical properties.

In addition, the response of the self-recoverable ML to varying stretching strains was systematically investigated. As shown in Fig. 2f, the UVC ML intensity of the SBO:Pr/PDMS elastomer film increases progressively with increasing applied stretching strain. The corresponding stress-strain curve is provided in Fig. S14, with detailed mechanical parameters summarized in Table S2. Notably, the inset of Fig. 2f reveals an approximately linear correlation between ML intensity and applied stress, indicating potential for stress-sensing applications. Although no visible fracture was observed under high strain, the film exhibited signs of irreversible plastic deformation beyond a certain threshold (Fig. S15). After being naturally placed at room temperature for 24 h, the film subjected to 60% strain recovered its original state (Fig. S16), whereas those stretched to 80% or 100% strain failed to return, suggesting the occurrence of permanent structural damage at these higher deformation levels (Fig. S17). These findings highlight the critical role of stretching strain in influencing the long-term mechanical integrity and optical performance of the elastomer film. A more detailed analysis of the strain-dependent ML behavior is presented in the following section.

The ML emission behavior of the SBO:Pr/PDMS elastomer film was further examined under varying tensile frequencies, as shown in Fig. S18. An increase in tensile frequency leads to a continuous enhancement of ML intensity. This can be attributed to the mechanical response of the polymer matrix: at higher strain rates, the polymer chains are unable to fully extend and reorient in response to deformation, necessitating greater external force to achieve the same elongation (Fig. S19)^61,62^. These findings underscore the importance of analyzing the intrinsic mechanical properties of the elastomer when interpreting ML behavior in inorganic–organic composite systems. In addition to stretching, the SBO:Pr/PDMS elastomer film also exhibits intense ML emission under continuous friction. As shown in Fig. 2g, when subjected to a 5 N frictional force (stress:1.58 MPa), the film emits intense UVC ML corresponding to Pr^3+^ transitions. This emission is clearly captured by a solar-blind UV camera, with the corresponding UVC overlay image shown in the inset. These results highlight the elastomer film’s versatile responsiveness to various mechanical stimuli, reinforcing its potential for practical applications in wearable sensing and intelligent interfaces.

### Cyclic stability and self-recovery behavior of UVC ML elastomer

**Fig. 3:**
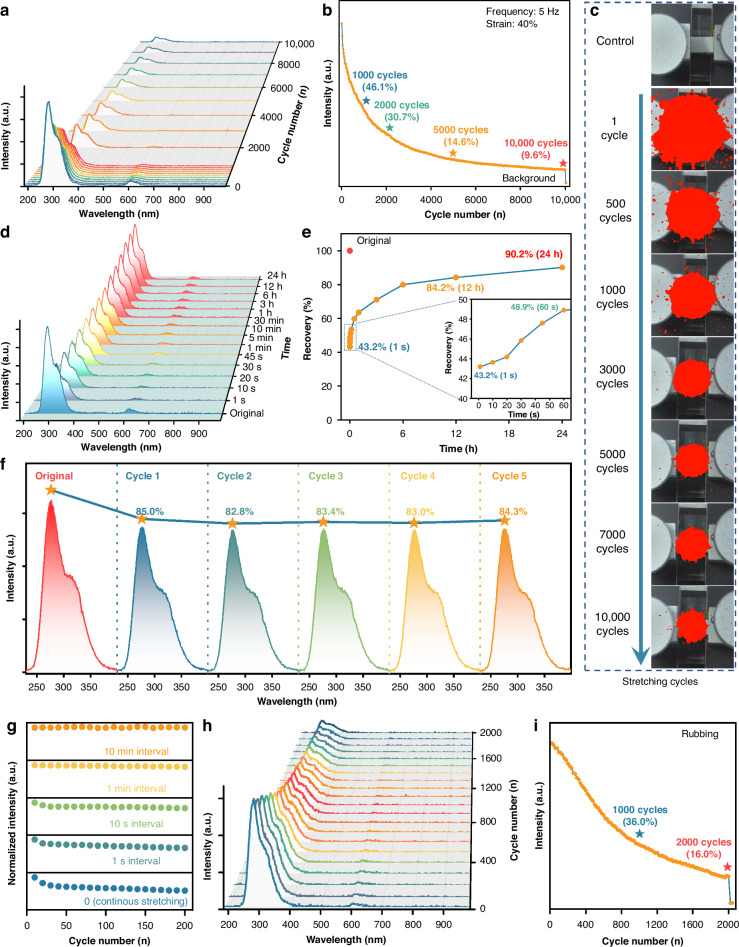
**Cyclic stability and self-recovery behavior of UVC ML elastomer**. **a** ML emission spectra and **b** ML intensity evolution of the SBO:Pr/PDMS elastomer film over 10,000 continuous stretching cycles (frequency: 5 Hz; strain: 40%). **c** Corresponding UVC ML images of the film recorded throughout the 10,000 stretching cycles. **d** ML emission spectra and **e** ML intensity recovery of the elastomer film after being naturally placed at room temperature for varying durations from 1 s to 24 h following 10,000 continuous stretching. **f** ML emission spectra over five consecutive cycles following 12 h of natural recovery. **g** ML repeatability performance under various time intervals between mechanical stimuli. **h** ML emission spectra and **i** corresponding intensity evolution of the elastomer film subjected to 2000 continuous rubbing cycles (frequency: 5 Hz; applied force: 5 N)

The cyclic stability, repeatability, and self-recovery of ML elastomers are critical parameters for evaluating the practical viability in advancing display, imaging, and sensing applications^58^. In this study, the SBO:Pr/PDMS composite elastomer exhibits excellent UVC ML performance, characterized by superior cyclic stability and rapid, effective self-recovery. Figs. 3a and S20 present the ML emission spectra of the elastomer film over 10,000 continuous stretching cycles (frequency: 5 Hz; strain: 40%), while Fig. 3b tracks the corresponding evolution of UVC ML intensity. During the initial cycling (1st to 2000th cycle), the UVC ML intensity rapidly attenuates, dropping to 46.1% and 30.7% of its initial value by the 1000th and 2000th cycle, respectively. However, beyond this point, the decay rate significantly slows, and by the 10,000th cycle, the ML intensity remains at 9.6% of the initial value, indicating a transition to a stable emission state. The decay of ML intensity arises from the adsorption variation between the phosphor and the polymer matrix (e.g., rupture and reformation of the physical adsorptions or hydrogen bonds binding) during cyclic stretching. Moreover, the ML power intensity was measured and displayed as ~6.2 mW m^−2^ at the 1st stretching (Figs. S21 and S22). Importantly, the ML spectral profile remains unchanged throughout 10,000 cycles, aside from ML intensity variation, confirming that the emission originates from the doped Pr^3+^ emitters and demonstrating excellent structural and spectral stability of the elastomer. Notably, even after 10,000 stretching cycles at 40% strain, the UVC ML intensity remains over an order of magnitude above the background signal and is readily detectable, underscoring the outstanding ML cyclic stability of the SBO:Pr/PDMS elastomer system. Furthermore, the UVC ML images during the 10,000 continuous stretching cycles were visually recorded, as depicted in Fig. 3c and Video S2 (the first 1000 stretching cycles). The progressive decrease in the red area graphically illustrates the decline in UVC ML intensity. In the early cycles (1–2000 cycles), the rapid shrinkage of the red region reflects sharp attenuation, consistent with the spectral data in Fig. 3a, b. In later cycles, the gradual reduction of the red region signifies a more stabilized decay. Remarkably, the red signal remains clearly visible even after 10,000 cycles, further validating the outstanding ML cyclic stability of the SBO:Pr/PDMS composite elastomer.

Although the UVC ML intensity of the SBO:Pr/PDMS composite elastomer attenuates during a continuous stretching cycle, the interfacial interactions can partially self-repair once the mechanical stimulus ceases, enabling a distinct self-recovery behavior. Figure 3d, e presents the UVC ML emission spectra and corresponding intensity recovery of the elastomer film after being naturally placed at room temperature for various durations ranging from 1 s to 24 h. Prior to the measurement, the film was subjected to 10,000 continuous stretching cycles. Strikingly, after just 1 s of rest at room temperature, the UVC ML intensity recovered to 43.2% of its initial value without any external intervention, indicating a rapid self-recovery process, which is attributed to fast interfacial relaxation and partial reconstruction of phosphor–polymer interactions. Prolonging the resting time significantly enhances this recovery, but the recovery proceeds more slowly, exhibiting a quasi-logarithmic relationship between ML intensity and rest duration, which is dominated by gradual chain rearrangements. Notably, after 12 h and 24 h of natural rest, the ML intensity recovered to 84.2% and 90.2% of its initial value, respectively, underscoring the efficient and passive self-recovery capacity of the elastomer. Throughout this process, the emission spectral profile remained unchanged, further confirming the excellent spectral stability of the elastomer.

To evaluate the reproducibility of the self-recovery behavior, the ML emission spectra were recorded over five consecutive cycles involving pre-stretching followed by 12 h of rest (Fig. 3f). In each cycle, the ML intensity consistently returned to approximately 84.0% of the initial value, demonstrating excellent repeatability and reliability. Furthermore, the relationship between the time interval and ML degradation was explored. As shown in Figs. 3g and S23, increasing the resting interval from 0 to 10 min significantly reduced the rate of ML intensity attenuation. At a 10-min interval, the ML intensity exhibited only minor fluctuation, indicating a stabilized emission output. These results suggest that the ML performance of the elastomer can be effectively modulated by controlling the interval between mechanical stimuli, offering enhanced tunability for practical applications.

Furthermore, the ML behavior of the SBO:Pr/PDMS elastomer film under continuous rubbing was investigated under a constant applied force of 5 N. The corresponding UVC ML spectra and intensity evolution over 2000 rubbing cycles (frequency: 5 Hz; applied force: 5 N) are shown in Figs. 3h, i and S24. Notably, the emission spectra remain consistent throughout the entire rubbing process, indicating stable spectral characteristics and excellent cyclic durability of the ML emission under repeated frictional stimuli. However, the UVC ML intensity exhibits a gradual attenuation with increasing rubbing cycles. Specifically, although the initial decline in intensity during the first 1–200 cycles is slower than that observed under stretching conditions (Fig. S25), the UVC ML intensity decreases to 36.0% and 16.0% of its initial value after 1000 and 2000 cycles, respectively, exhibiting a faster attenuation. Critically, even after 2000 cycles, the UVC ML signal remains well above the background level and is clearly detectable. A visual representation of this process is provided in Video S3. The observed accelerated attenuation in UVC ML intensity under prolonged rubbing is likely due to localized structural degradation at the frictional contact interface. As evidenced in Fig. S26, significant abrasion and material degradation are observed along the frictional trace, suggesting that continuous mechanical friction progressively disrupts the interfacial interactions between the SBO:Pr particles and the PDMS matrix. Such degradation likely impairs triboelectric charge transfer at the interface, thereby reducing the efficiency of UVC ML generation over time.

### The effect of mechanical behavior on the performance of UVC ML

**Fig. 4:**
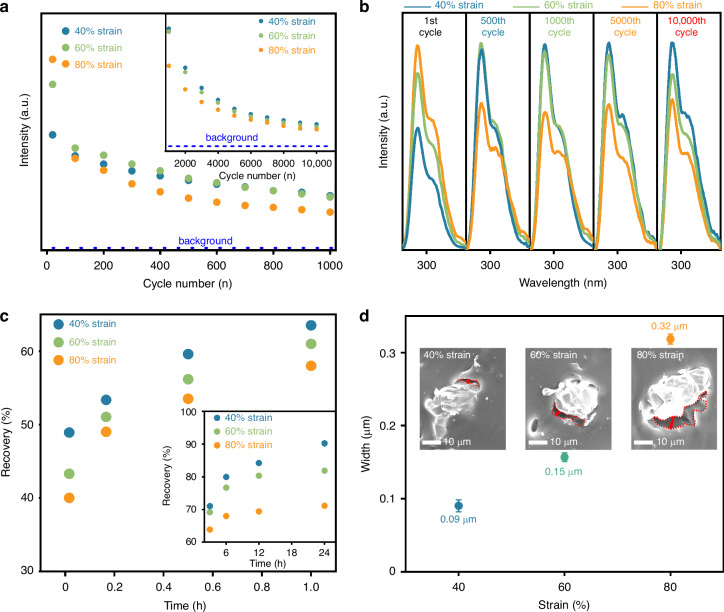
**The effect of mechanical behavior on the performance of UVC ML**. **a** Evolution of ML intensity over 10,000 stretching cycles, and **b** corresponding ML emission spectra over varied stretching cycles of the SBO:Pr/PDMS elastomer film under varying numbers of stretching cycles at different strain levels (40%, 60% and 80%, frequency: 5 Hz). **c** ML intensity recovery of the SBO:Pr/PDMS elastomer film after natural rest periods ranging from 1 min to 24 h at room temperature, following different stretching strains (40%, 60%, and 80%; frequency: 5 Hz). **d** Interfacial gap width between the SBO:Pr particles and the PDMS matrix under different stretching strains. Insets show the corresponding cross-sectional SEM images

Since the SBO:Pr/PDMS composite elastomer comprises SBO:Pr microcrystals embedded in a PDMS matrix, its ML behavior is closely related to mechanical strain and interfacial interactions. As illustrated in Figs. S15–S17, the elastomer films undergo irreversible deformation at high strain levels, suggesting strain-dependent UVC ML properties. To examine this, UVC ML intensity evolution and emission spectra were measured over 10,000 continuous stretching cycles (strain: 40%, 60%, and 80%, frequency: 5 Hz), as shown in Figs. 4a, b, S27 and S28. Initially, ML intensity increases with strain due to enhanced triboelectric excitation. However, higher strains lead to faster signal degradation—ML intensity drops to 46.1% at 40% strain, 31.0% at 60%, and 19.0% at 80% by the 1000th cycle. This trend indicates that while greater strain elevates initial ML output, it concurrently accelerates mechanical fatigue and interfacial degradation, thereby reducing ML cyclic stability. Under 40% stretching strain, the SBO:Pr/PDMS elastomer film exhibits low initial ML emission intensity but excellent cyclic stability due to its elastic, damage-free deformation. In contrast, exceeding 60% strain induces irreversible structural damage that significantly compromises its cyclic stability. At 60% strain, the film initially shows higher ML intensity than at 40% due to greater stress, but decays more rapidly over cycles. After approximately 800 cycles, the intensities at 40% and 60% strains converge due to partial damage at 60% strain, marking a transition from elastic to early plastic deformation. At 80% strain, permanent damage, primarily from the partial detachment of SBO:Pr particles, results in rapid ML attenuation despite a strong first-cycle emission. These strain-dependent behaviors are reflected in the UVC ML emission spectra over 10,000 cycles (Figs. 4b, S27 and S28). Notably, all elastomer films maintain consistent spectral profiles throughout the test, confirming that the UVC ML originates from the same luminescent centers and the elastomer system has excellent spectral stability under repeated mechanical stimuli.

The self-recovery behavior of the SBO:Pr/PDMS elastomer films under different stretching strains is shown in Figs. 4c, S29 and S30. All films exhibited rapid recovery, achieving at least 40.0% recovery within 1 min of natural rest at room temperature, confirming the rapid self-recovery behavior of the elastomer film. However, higher stretching strains resulted in slower recovery kinetics and reduced final recovery levels. After 24 h, recovery reached 90.2% for 40% strain, but only 81.9% and 71.1% for 60% and 80% strains, respectively. This strain-dependent recovery degradation is attributed to increased microstructural damage induced by higher strains, which compromises both cyclic stability and self-recovery performance. These results underscore the importance of maintaining the composite film within its elastic deformation range to preserve optimal mechanical and luminescent functionality.

The variations in UVC ML performance under different stretching strains are primarily due to progressive interfacial degradation between the phosphor particles and the PDMS matrix. As shown in Fig. S31, after 10,000 continuous stretching cycles, the film strained at 40% maintained an intact surface, while the 60%-strained film showed minor damage, and the 80%-strained film exhibited severe degradation with evident detachment of phosphor particles—accounting for its significantly reduced cyclic stability and impaired self-recovery. Cross-sectional SEM images (Figs. 4d and S32) further confirm strain-induced interfacial deterioration, with wider interfacial gaps between the SBO:Pr microcrystals and the PDMS matrix as strain increases. Quantitative analysis reveals a higher probability of the formation of interfacial gaps with increasing applied strain, indicating weakened adhesion at the phosphor–matrix interface. These results highlight that stretching strain critically affects interfacial structural integrity, thereby directly influencing UVC ML performance. Monitoring such interfacial dynamics during mechanical loading is essential for designing durable and high-performance UVC ML composite elastomers.

### UVC ML mechanism

At the macroscopic level, ML is observed as a force-to-light conversion phenomenon, while at the microscopic level, it involves interfacial electron transfer processes. To elucidate the self-powered and self-recovered ML mechanism, we investigated three potential contributing factors: piezoelectricity, thermoluminescence (TL), and triboelectricity. Piezoresponse force microscopy (Fig. S33) reveals that the SBO host is intrinsically non-piezoelectric, and Pr^3+^ doping does not break the symmetry, thus excluding piezoelectricity as a driving force. TL measurements (Figs. S34–S36) confirm the absence of electron trapping states in SBO:Pr phosphor after 30-day ambient illumination and illustrate no detectable change in the ML intensity or spectral profile even after pre-irradiation (UV and X-ray), effectively ruling out trapped-electron involvement in the UVC ML process. These results highlight the necessity of further investigating alternative interfacial charge generation mechanisms—such as triboelectric effects—to account for the observed ML behavior.

**Fig. 5:**
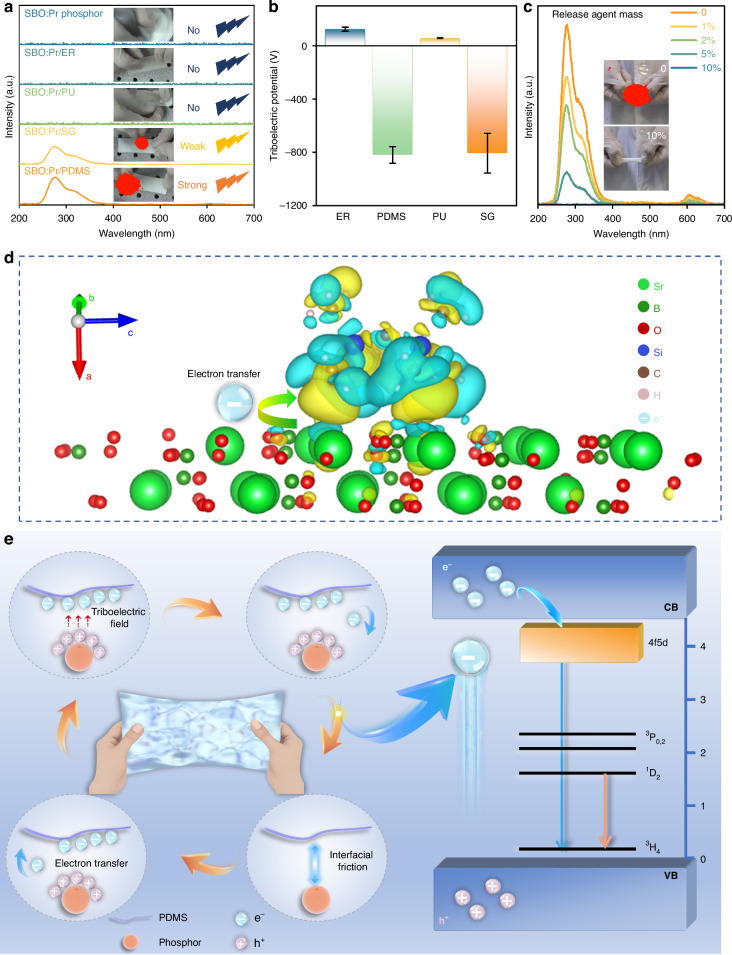
**UVC ML mechanism**. **a** UVC ML emission spectra of SBO:Pr phosphors embedded in different matrices (ER, PU, SG, and PDMS) under rubbing stimuli. Insets show the corresponding UVC ML images. **b** Triboelectric potential of the ER, PDMS, PU, and SG after rubbing with the SBO:Pr phosphor under 120 rpm for 1 min. **c** UVC ML emission spectra of SBO:Pr/PDMS elastomer film with varying mass ratios of release agent to PDMS. The insets show UVC ML images of the film containing 0 and 10% release agent. **d** Simulated electron-gain (yellow) and electron-loss (light-blue) regions at the SBO-PDMS monomer interface. **e** Schematic diagram of the proposed UVC ML mechanism of the SBO:Pr/PDMS elastomer film under mechanical stimuli

Given the intense UVC ML response of the SBO:Pr/PDMS elastomer under mechanical stimulation, where unavoidable contact-separation cycles occur at phosphor–matrix interfaces, it is reasonable to conclude that interfacial electron transfer between the PDMS matrix and embedded phosphor particles plays a critical role in the observed ML phenomenon. To validate this hypothesis, we systematically compared the UVC ML emission behaviors of SBO:Pr phosphor embedded in different polymer matrices, including epoxy resin (ER), polyurethane (PU), silicone gel (SG), and PDMS, under identical rubbing conditions (Fig. 5a), and the insets show the corresponding UVC ML images. Only the SBO:Pr/PDMS and SBO:Pr/SG systems exhibited intense and observable UVC ML, with SBO:Pr/PDMS system showing higher UVC ML intensity. In contrast, the pristine SBO:Pr phosphor powder, as well as the SBO:Pr/ER and SBO:Pr/PU systems, showed no detectable ML emission signal under the same mechanical conditions. Moreover, we also used various expanded organic matrices, including PVDF, PTFE, FEP, PFA, and PMMA; however, none of these systems exhibited detectable UVC ML signals (Fig. S37). These results strongly support that matrix-specific interfacial interactions are essential for facilitating electron transfer processes responsible for UVC ML generation. Only when the phosphor is embedded in specific matrices, such as PDMS and SG, can effective electron transfer between the phosphor particles and matrix occur, enabling intense UVC ML emission.

To further elucidate the interfacial interactions mechanism, we propose that the mechanical stimuli inevitably induce contact-separation cycles at the phosphor–matrix interface, leading to triboelectrification^63,64^. This process could generate localized triboelectric potentials that drive interfacial electron transfer. To validate this, the triboelectric properties of the SBO:Pr phosphor in contact with different polymers were investigated, as shown in Figs. 5b and S38. Before the test, the probe was calibrated, and the blank potential in air was set to 0. After 1 min of frictional contact with SBO:Pr phosphor disc, PDMS, and SG matrices exhibited strong negative surface potentials, indicating substantial electron acquisition from the phosphor particles. In contrast, ER and PU matrices developed positive surface charges, signifying electron loss. Notably, UVC ML emission was observed exclusively in the SBO:Pr/PDMS and SBO:Pr/SG systems, where the matrix becomes negatively charged upon contact with the phosphor. This correlation strongly supports a mechanism involving direction-specific electron transfer from the phosphor particles to the polymer matrix. Although both PDMS and SG exhibited similar triboelectric potentials, the SBO:Pr/PDMS system showed significantly higher UVC ML intensity. This discrepancy is likely attributable to their distinct optical transmittance (Fig. S39), which influences the UVC photon transmission efficiency and ultimately affects the observed UVC ML intensity.

To clarify the role of interfacial triboelectricity in UVC ML generation, we examined the ML emission spectra of SBO:Pr/PDMS elastomer films prepared with varying amounts of release agent, which acts as a lubricant to reduce interfacial friction. Since interfacial triboelectric interactions are essential for ML generation, modulating this friction offers insight into the correlation between interfacial triboelectrification and ML intensity. As illustrated in Fig. 5c, increasing the release agent content leads to a gradual decrease in ML intensity, with complete quenching of ML emission at 10% release agent. It should be noted that the incorporation of release agent exhibits almost no impact on either the UVC PL properties of the film (Fig. S40) or the optical transmittance of the PDMS matrix (Fig. S41). These results further confirm that UVC ML emission originates from triboelectric charge transfer at the phosphor–matrix interface.

To further elucidate the interfacial triboelectricity mechanism, we analyzed the electron transfer between the SBO crystal and PDMS matrix by density functional theory (DFT)^65^. Because PDMS is an insulator with localized electron distributions along its molecular chains, it is appropriate to simplify the model by replacing the full polymer chain with a monomer to study its electronic behaviors. Based on the most stable SBO/PDMS contact model (Fig. S42), we calculated the electron density difference (ρ_diff_), which reflects the changes in electron density distributions before and after contact electrification. ρ_diff_ is defined as:

1 $${\rho }_{\mathrm{diff}}={\rho }_{\mathrm{SBO}/\mathrm{PDMS}}-{\rho }_{\mathrm{SBO}}-{\rho }_{\mathrm{PDMS}}$$

where *ρ*_SBO/PDMS_ is the electron density distribution of the SBO/PDMS after contact; *ρ*_SBO_ and *ρ*_PDMS_ are electron density distributions of the SBO crystal and PDMS monomer before contact, respectively. The resulting electron-gain (yellow) and electron-loss (light-blue) regions at the SBO-PDMS interface are shown in Figs. 5d and S43. Due to the disorder of amorphous polymer models, the electron-gain and -loss regions are mixed together. However, the crystal surface is dominated by electron-loss regions, with only a few fragmented electron-gain regions, attributed to the existence of covalent bonds within the SBO crystal. This indicates that the electron transfer from the SBO microcrystal to the PDMS matrix, consistent with triboelectric potentials measurements. These findings reinforce that interfacial electron transfer is responsible for the underlying UVC ML mechanism.

Based on the aforementioned results and discussions, we propose a mechanistic model to elucidate the origin of the intense UVC ML, as illustrated in Figs. 5e and S44. Under mechanical stimulation, the contact-separation at the phosphor-PDMS interface occurs, and interfacial friction induces electron transfer from the SBO:Pr phosphor particles to the PDMS matrix, generating a triboelectric field at the interface. In this process, the elastomer absorbs mechanical energy, which is converted into electrostatic potential energy through the triboelectric effect. The electron transfer results in the accumulation of positive electrostatic charges (holes) on the particle surface. Subsequently, electrons on the surface of PDMS are attracted back to the SBO:Pr particles under the influence of the interfacial triboelectric field. Consequently, the electron-hole recombination occurs at the particle–matrix interface in a short time to release excitation energy rather than being emitted directly as light. It can promote electrons in SBO:Pr from the VB to the CB. These excited electrons then transfer to the 4f5d energy level of Pr^3+^ ions and subsequently relax radiatively to the 4f² levels, producing UVC ML.

### Self-powered photonics applications

**Fig. 6:**
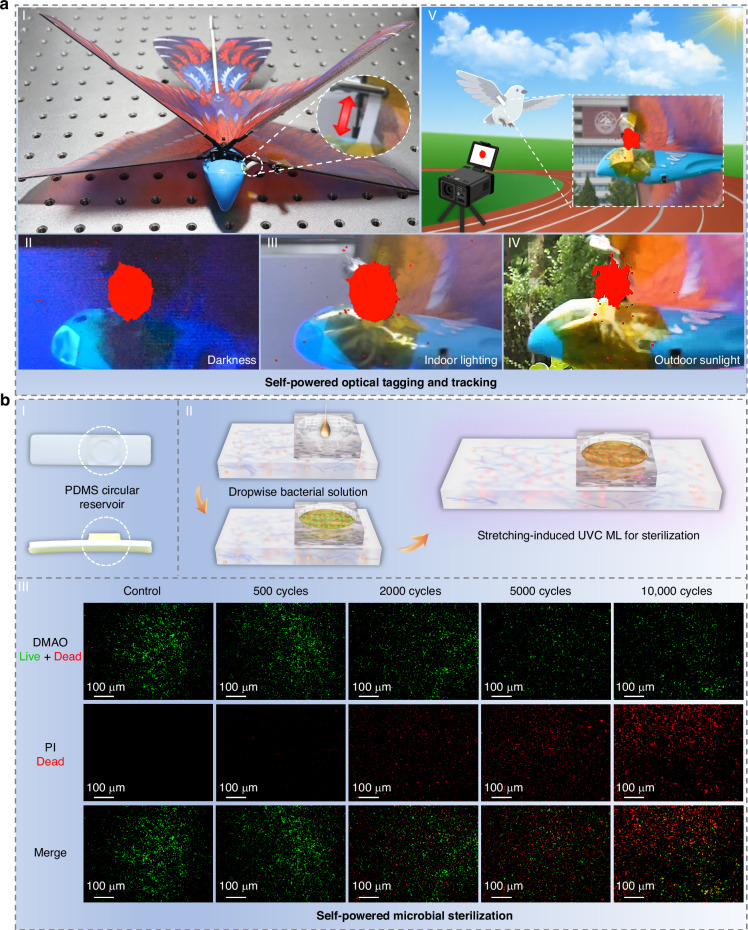
**Self-powered photonics applications**. **a** Demonstration of self-powered optical tagging and tracking. I: Image of a remote-controlled bird model. Inset shows the enlargement of the attached elastomer film. II–IV: UVC ML images of wing flap under dark, indoor-lighting and outdoor sunlight conditions (distance: 3 m). V: In-flight demonstration of UVC ML as a visible signaling source. **b** Demonstration of self-powered microbial sterilization. I–II: Schematic illustrations of the sterilization process. III: Inactivation of *Escherichia coli* by UVC ML after various stretching cycles

Taking advantage of the solar-blind nature of UVC light (Fig. S45 and Video S4), along with the self-powered and self-recovered ML characteristic and easily detectable UVC ML signals of the SBO:Pr/PDMS elastomer (Fig. S46), we first demonstrated its potential for self-powered optical tagging and tracking applications. The elastomer film was attached to the wing of a remote-controlled bird model (Fig. 6aI), where vertical flapping motion generated sufficient mechanical stimulus to trigger intense UVC ML emission, enabling effective optical tagging and tracking under various environmental and lighting conditions (Fig. 6aII–IV and Video S5). Building on this concept, we further showcased in-flight UVC ML as a visible signaling source (Video S6), highlighting its practical utility in emergency scenarios such as distress signaling and search-and-rescue operations (Fig. 6aV). Additionally, we proposed a bright-field stress visualization application for structural damage monitoring in mechanical systems (Fig. S47), enabling real-time observation of crack formation and expansion.

In addition to its covert optical tagging capabilities in bright indoor and outdoor environments, the UVC ML also offers promising potential for self-powered microbial sterilization applications. To evaluate its bactericidal performance under mechanical stimulation, a PDMS circular reservoir was integrated onto the film surface to hold bacterial suspensions during continuous stretching (Fig. 6bI), with a schematic illustration of the sterilization process shown in Fig. 6bII. A series of bactericidal experiments were conducted to assess the inactivation effect of the UVC ML on *Escherichia coli* (EC) and Methicillin-resistant *Staphylococcus aureus* (MRSA). As shown in Fig. 6bIII, EC exhibited progressively greater inactivation with increasing stretching cycles. Notably, the laser scanning confocal microscopy revealed that the majority of EC were inactivated after 10,000 stretching cycles, indicating that the mechanically induced UVC ML effectively causes bacterial death. Similar results were observed for MRSA (Fig. S48), confirming the strong bactericidal efficacy of the elastomer film (Fig. S49, 88.9% mortality rate for EC and 70.1% for MRSA). These findings demonstrate the significant potential of the UVC ML elastomer for the development of mechanically responsive, efficient and “power-free” sterilization systems.

### ML spectral tunability

Beyond the intense UVC ML exhibited by the SBO:Pr/PDMS elastomer film, other SBO:Ln/PDMS composite systems (Ln = Gd^3+^, Dy^3+^, Sm^3+^, Tm^3+^, Ce^3+^, Tb^3+^, Eu^3+^, and Eu^2+^) also demonstrate pronounced ML under mechanical stimulation, as shown in Figs. S50–S58. Upon stretching, each elastomer film exhibits characteristic emission corresponding to its respective doped lanthanide ion, confirming that lanthanide-doped Sr_3_(BO_3_)_2_ is well-suited for integration with the PDMS matrix to produce intense ML emission. Moreover, the SBO:Ln phosphor exhibits the same selectivity with the various polymer matrices, as shown in Figs. S59–S66, further demonstrating the robust interfacial interactions between the SBO:Ln phosphor and the PDMS matrix. Notably, the SBO:Gd/PDMS, SBO:Dy/PDMS, SBO:Sm/PDMS, SBO:Tm/PDMS and SBO:Tb/PDMS elastomers show persistent ML, with detectable emission continuing even after cessation of mechanical stimulus. This afterglow behavior is likely attributed to delayed electron transfer processes. The ML performance of the SBO:Dy/PDMS elastomer film over 10,000 stretching cycles is shown in Fig. S67, where it consistently emits visible ML, with the persistent luminescence lasting up to 10 s after stretching stops.

## Discussion

In conclusion, we report robust self-powered and self-recovered solar-blind UV ML in an SBO:Pr/PDMS inorganic–organic composite elastomer system under various mechanical stimuli. The effect of stretching strain on ML cyclic stability and self-recovery was systematically investigated, revealing that 40% strain does not compromise the microstructure of the film, thereby enabling optimal UVC ML performance. The elastomer exhibits excellent repeatability and cyclic durability, maintaining a detectable UVC emission over 10,000 continuous stretching cycles (power intensity at 1st cycle is ~6.2 mW m^−2^). It also demonstrates rapid and efficient self-recovery behavior, restoring 43.2% of its initial UVC ML intensity within 1 s of natural rest, and up to 90.2% after 24 h. Comprehensive experimental and theoretical studies elucidate that the UVC ML originates from interfacial triboelectrification, driven by electron transfer from the SBO:Pr phosphor to the PDMS matrix. Leveraging the solar-blind nature and high photon energy of UVC light, we further demonstrate emerging applications of the solar-blind UV ML elastomer in self-powered optical tagging and microbial sterilization (88.9% mortality rate for EC and 70.1% for MRSA). This study expands ML research to the solar-blind UV spectral region and brings ML applications to light.

## Materials and methods

### Materials

SrCO_3_ (99.99%, Aladdin), H_3_BO_3_ (99.99%, Aladdin), Gd_2_O_3_ (99.99%, Aladdin), Dy_2_O_3_ (99.99%, Aladdin), Sm_2_O_3_ (99.99%, Aladdin), Tm_2_O_3_ (99.99%, Aladdin), and Eu_2_O_3_ (99.99%, Aladdin) were used as the starting chemicals without further treatment. In order to introduce Pr^3+^, Ce^3+^ and Tb^3+^ ions precisely, a Pr(NO_3_)_3_ (Ce(NO_3_)_3_/Tb(NO_3_)_3_) solution (1 mol L^−1^) was prepared using Pr(NO_3_)_3_‧6H_2_O (99.99%, Aladdin), Ce(NO_3_)_3_‧6H_2_O (99.99%, Aladdin), Tb(NO_3_)_3_‧6H_2_O (99.99%, Aladdin). PDMS (Dowsil 184) and Release Agent 184 were received from Dow Corning. Additional polymer matrices, including SG (Shenzhen Guoyuan), PU (Beijing Haibeisi), and ER (Shandong Deyuan), were used. Commercial ZnS:Cu powder was directly purchased online.

### Synthesis of phosphor

Sr_3-*x*_(BO_3_)_2_:*x*Pr^3+^ (0 < *x* ≤ 0.015) phosphors were synthesized via a traditional high-temperature solid-state reaction method. Stoichiometric amounts of raw materials were weighed accurately. First, the raw materials were ground thoroughly in an agate mortar, and then the mixture was pre-fired at 600 °C in air for 2 h. After that, the pre-fired powders were ground again and sintered at 1050 °C for 5 h in air. Finally, after cooling to room temperature, the SBO:Pr powders were obtained by grinding. The synthesis of the SBO:Ln (Ln = Gd^3+^, Dy^3+^, Sm^3+^, Tm^3+^, Ce^3+^, Tb^3+^ and Eu^3+^) is similar to the SBO:Pr. For the synthesis of SBO:Eu^2+^, the sintering is carried out under the atmosphere of 95% N_2_ and 5% H_2_ in a tube furnace.

### Fabrication of SBO:Pr/PDMS (ER, PU, SG) films

The SBO:Pr phosphors were mixed with the PDMS precursor and the curing agent with a weight ratio of 1:1.5:0.15. Then the mixture was mixed in a glass mortar using mechanical stirring for 10 min. After that, the mixture was poured into a rectangular polytetrafluoroethylene (PTFE) mold. After curing at 80 °C for 2 h, the SBO:Pr/PDMS composite films were obtained. The SBO:Pr powders were mixed with the ER, PU, and SG precursor with a weight ratio of 1:2:0.6, 1:1:1, and 1:1:1, and the preparation processes followed the same procedure as for SBO:Pr/PDMS composite films.

### Characterization

The crystal structure of the as-synthesized samples was characterized via powder XRD (DMAX-2500PC, Rigaku) with Cu Kα (40 kV, 100 mA) irradiation (*λ* = 1.5418 Å) in a scanning speed of 10° min^−1^. The morphology and element distribution were analyzed using a field-emission high-temperature scanning electron microscope (SEM, JSM-7800F, JEOL) equipped with an energy-dispersive X-ray spectroscope (EDS). The XPS analysis was performed on Shimadzu AXIS SUPRA. The color optical micrographs were recorded on the optical microscope (Aosvi). The actual Pr^3+^ concentrations were measured by ICP-MS (Agilent-7800). The stress-strain curves were measured using a UTM6103X universal testing machine (Shenzhen Suns Technology Stock Co., Ltd.). The photoluminescence, radioluminescence, and PLQY were measured using an FLS1000 spectrofluorometer (Edinburgh Instruments) equipped with a photomultiplier tube detector (PMT, 200-900 nm), a 400 W Xe lamp as the excitation source, and an integrating sphere. The ML spectra were measured using the fiber spectrometer (Ocean Insight QE-Pro). The thermoluminescence (TL) spectra were recorded using an SL18 thermoluminescence instrument (Guangzhou Rongfan Science and Technology Co., Ltd.). The transmission spectra were measured by a UV–VIS spectrophotometer (Shimadzu 2600i). Phase and amplitude of piezoresponse force microscopies were recorded by Atomic Force Microscope (MFP-3D Origin, Oxford). The power intensity was measured using a Newport 1936-R optical power meter equipped with a Newport 918D-UV-OD3R UV-enhanced silicon photodetector. The triboelectric potential was measured using a friction tester (MS-T3001, Lanzhou Huahui Instrument Technology Co., Ltd.) equipped with an electrostatic measurement probe (SK050, KEYENCE (Japan) Co., Ltd.). The cathodoluminescence (CL) spectrum was obtained through a device coupled to SEM (GeminiSEM560). The UVC luminescence images were recorded by using a solar-blind UV camera (ZH580, Shanghai Pumeng Optoelectronics Technology Co., Ltd.) with dual UVC (240–280 nm)-visible channels, which are overlay images after the addition of a UVC image onto a visible image.

### Computational details

The initial atomic positions and symmetry information of the SBO crystal structure were taken from the Inorganic Crystal Structure Database, and the periodic 2 × 2 × 1 supercell was used to simulate. Using the Vienna Ab initio simulation package^66^, theoretical simulations were carried out using the DFT, and the generalized gradient approximation-Perdew-Burke-Ernzerhof exchange-correlation functional was adopted. The energy change and the Hellmann-Feynman forces on atoms were set to 10^−5^ eV and 0.01 eV Å^−1^, respectively. The plane-wave cut-off energy was set at 520 eV. For k-point integration within the first Brillouin zone, a 3 × 3 × 3 Monkhorst–Pack grid was selected.

### Sterilization experiment

Suspensions of *Escherichia coli* (EC) and Methicillin-resistant *Staphylococcus aureus* (MRSA) (10^6^ cfu/mL) were inoculated into a PDMS circular reservoir (100 μL) positioned on the composite surface. Experimental groups consisted of SBO:Pr/PDMS composites subjected to various stretching cycles, while the control group was set be SBO/PDMS. After stretching, the bacterial solutions were stained using a Live/Dead Bacterial Staining Kit with DMAO and PI (C2030S, Beyotime), which could label live bacteria in green and dead bacteria in red and green. Next, the EC and MRSA bacterial suspensions were incubated in the dark for 15 min and then imaged using a confocal laser scanning microscope.

## Supplementary information

Supporting Information

UVC mechanoluminescence video of the SBO:Pr/PDMS elastomer under stretching, bending, and rubbing

UVC mechanoluminescence video of the SBO:Pr/PDMS elastomer under the first 1,000 stretching cycles

UVC mechanoluminescence video of the SBO:Pr/PDMS elastomer under the continuous rubbing cycles

Demonstration of the solar-blind nature of UVC light from the SBO:Pr/PDMS elastomer

Self-powered mechanoluminescence driven by flapping wings in a remote-controlled bird

Demonstration of self-powered optical tagging and tracking

## Data Availability

All the data supporting the findings of this study are presented within the article and its Supplementary Information. Additional data related to this paper are available from the corresponding authors upon request. Source data are provided within this paper.
