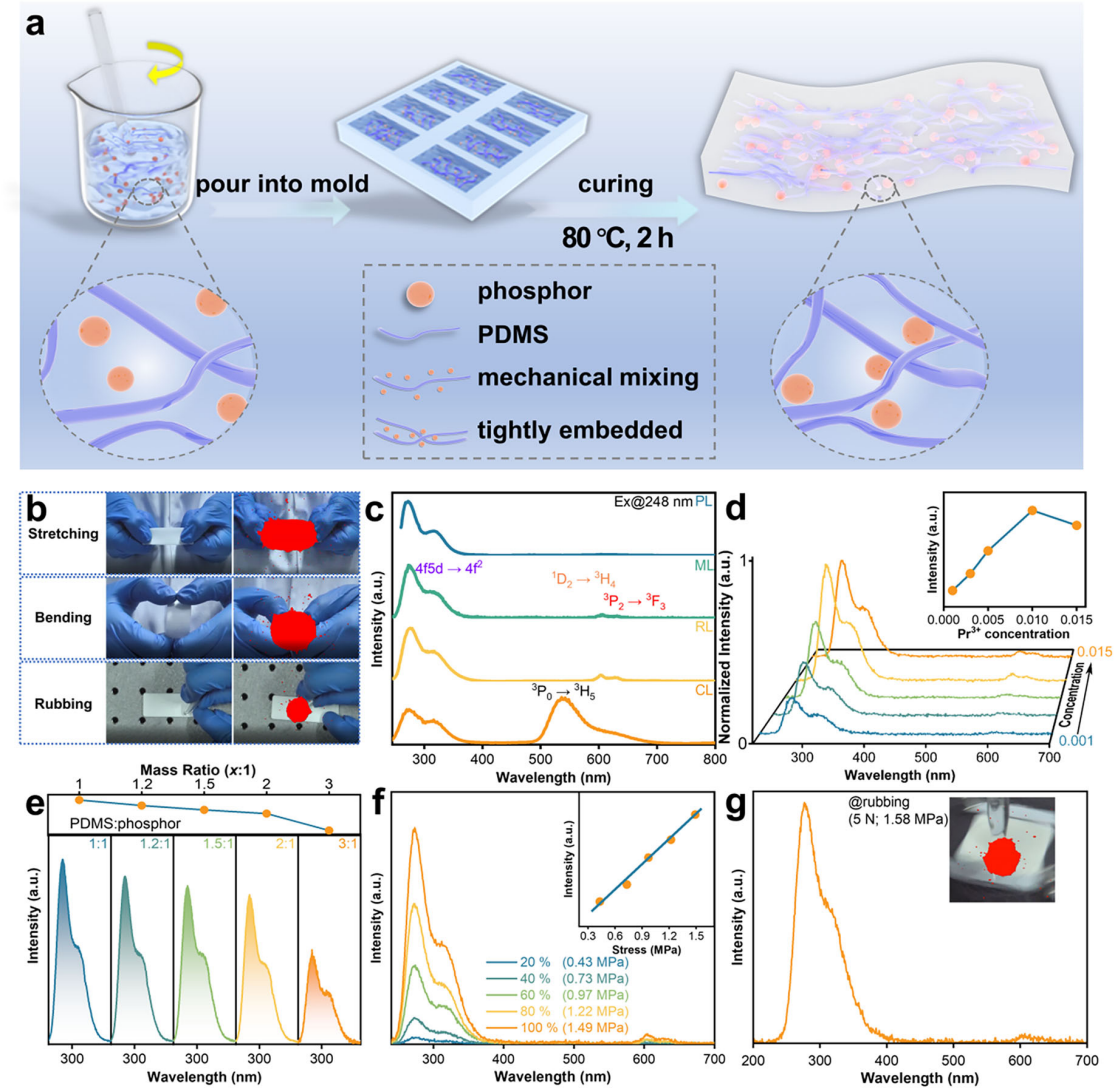# Correction: Self-powered mechanoluminescent elastomer for solar-blind ultraviolet emission

**DOI:** 10.1038/s41377-026-02213-9

**Published:** 2026-03-27

**Authors:** Xulong Lv, Tianyi Duan, Shaofan Fang, Zhaofeng Wang, Dongxun Chen, Lipeng Huang, Huanyu Liu, Zheming Liu, Chao Liu, Xiao-Jun Wang, Yanjie Liang

**Affiliations:** 1 School of Materials Science & Engineering, Shandong University, Jinan, 250061 China; 2 State Key Laboratory of Coatings for Advanced Equipment, Shandong University, Jinan, 250061 China; 3 School of Information and Communication Engineering, Beijing University of Posts and Telecommunications, Beijing, 100876 China; 4 Shandong Laboratory of Advanced Materials and Green Manufacturing at Yantai, Yantai, 264006 China; 5 State Key Laboratory of Solid Lubrication, Lanzhou Institute of Chemical Physics, Chinese Academy of Sciences, Lanzhou, 730000 China; 6 Department of Oral and Maxillofacial Surgery, School and Hospital of Stomatology, Cheeloo College of Medicine, Shandong University, Jinan, 250012 China; 7 Department of Oral and Maxillofacial Surgery, Qilu Hospital of Shandong University, Jinan, 250012 China; 8 Department of Physics, Georgia Southern University, Statesboro, GA 30460 USA

**Keywords:** Optical materials and structures, Electronics, photonics and device physics, Lasers, LEDs and light sources

Correction to: *Light: Science & Applications* 10.1038/s41377-025-02131-2, published online 12 January 2026

After the publication of this article, it was brought to our attention that some issues still require further revision.

the figure 3 has some errors: In Figure 3a, the x-axis tick label should be corrected from “500” (after “800”) to “900”. In Figure 3e, the x-axis label unit should be changed from “Time (s)” back to “Time (h)”. In Figure 3f, the text “85.0%” overlaps with the pentagram symbol and should be moved upward to prevent any visual obstruction and improve readability.

Commas are still added for the 4-digit numbers in Figure 4a, Figure 4b, and Figure 6b. In these figures, commas should also be deleted, including:

- In Figure 4a, the x-axis tick label “1,000” should be corrected to “1000”.
- In Figure 4a, the x-axis tick labels “2,000” “4,000” “6,000” and “8,000” in the inset should be corrected to “2000” “4000” “6000” and “8000”.
- In Figure 4b, the text “1,000th cycle” and “5,000th cycle” in the figure should be corrected to “1000th cycle” and “5000th cycle”.
- In Figure 6b III, the text “2,000 cycles” and “5,000 cycles” should be corrected to “2000 cycles” and “5000 cycles”.
  In the main text (Page 7, right column, line 2): “2,000” should be changed to “2000”.
  The unit “HZ” should be changed to “Hz”, including:
- Page 5, main text, right column, line 27.
- Page 6, the text in Figure 3b.
- Page 6, the caption of Figure 3b.
- Page 6, the caption of Figures 3h,i.
- Page 7, main text, right column, line 3.
- Page 8, the caption of Figure 4b.

We apologize for any inconvenience these errors may have caused. The original article has been updated.

Incorrect figure 3
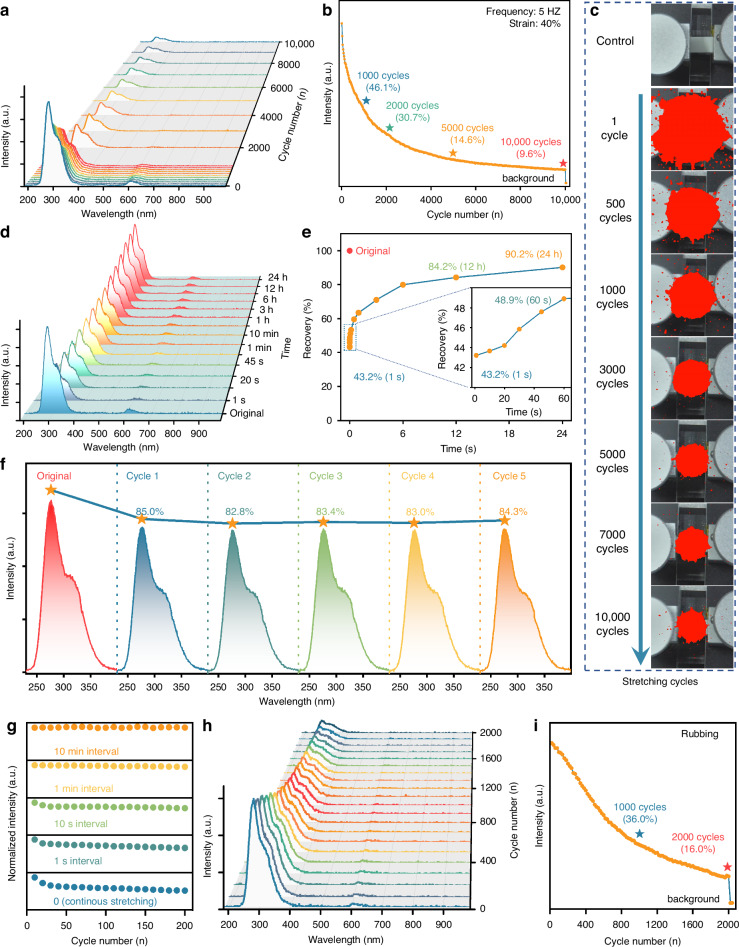


Correct figure 3
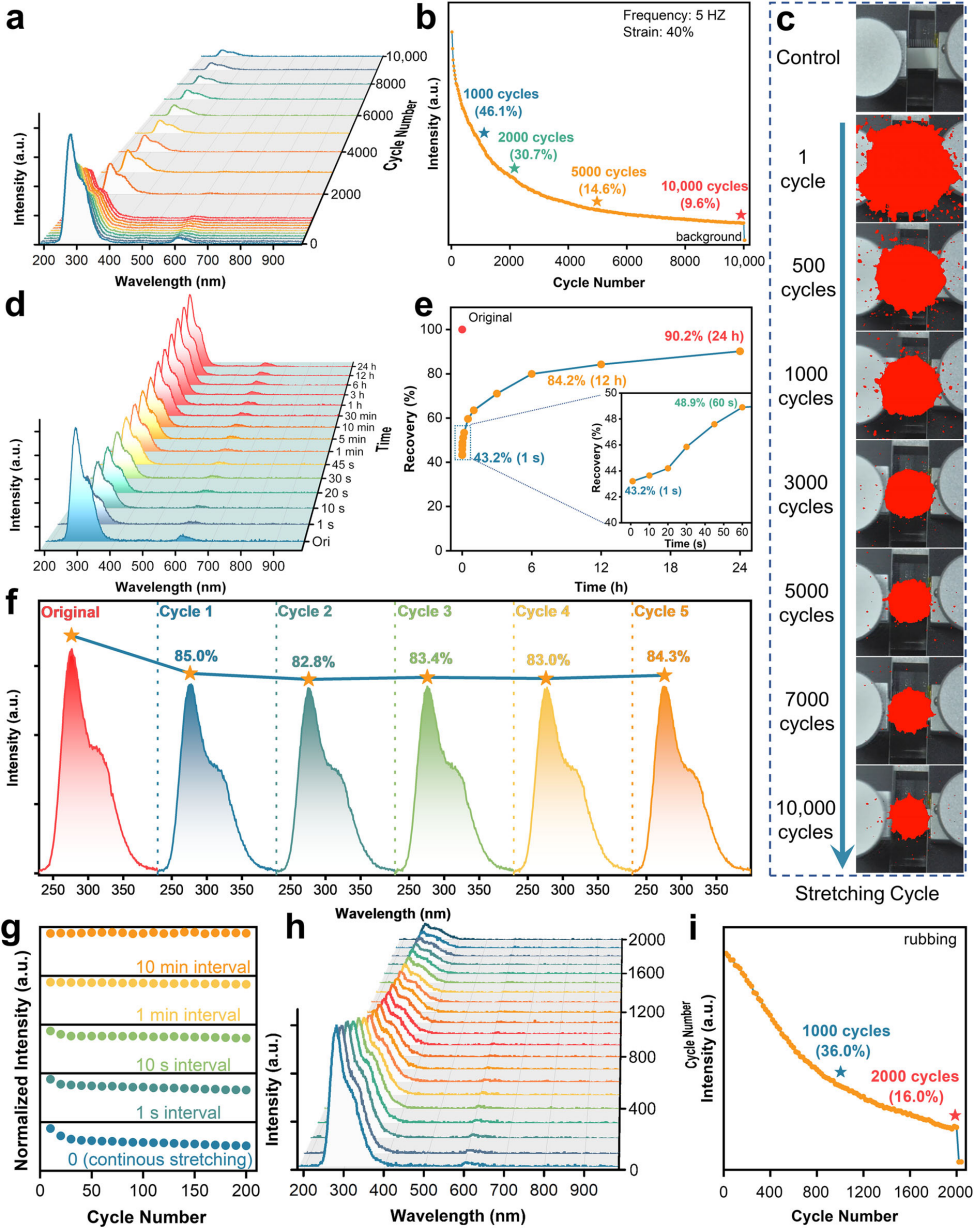


the figure 2 has some errors: Figure 2b will be moved up so that its top and bottom edges align horizontally with those of Figure 2c. The “Mass ratio (x:1)” text beneath Figure 2b will be adjusted downward to serve as the axis title for Figure 2e.

Incorrect figure 2
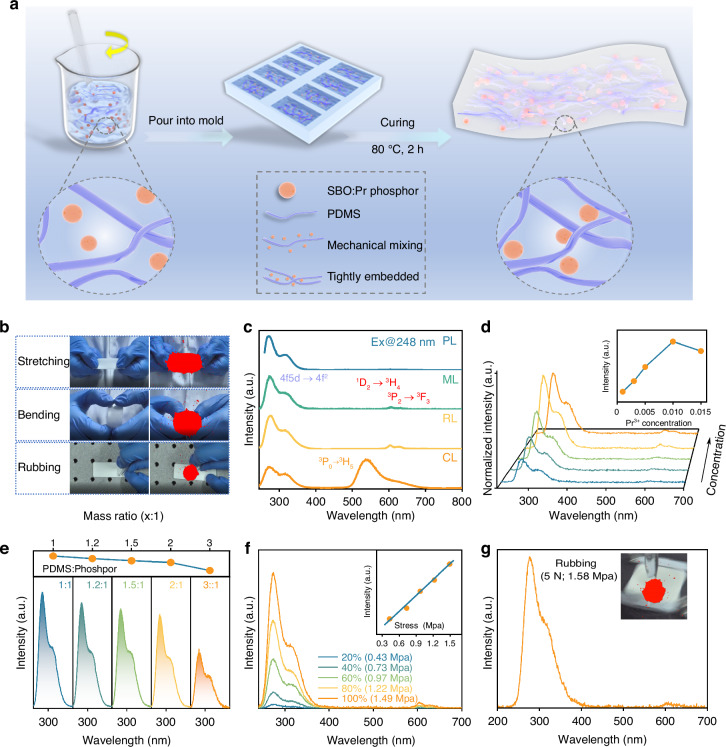


Correct figure 2