# Supplementary material for: Self-powered mechanoluminescent elastomer for solar-blind ultraviolet emission
Source: Light Sci Appl. 2026 Jan 12;15:61. doi: 10.1038/s41377-025-02131-2 (PMC12791142; doi:10.1038/s41377-025-02131-2)

**Supporting Information**

Self-powered mechanoluminescent elastomer for solar-blind ultraviolet emission

Xulong Lv^1,2^, Tianyi Duan^3^, Shaofan Fang^4^, Zhaofeng Wang^4,5^*, Dongxun Chen^1,2^, Lipeng Huang^1,2^, Huanyu Liu^6^, Zheming Liu^7^, Chao Liu^7^, Xiao-Jun Wang^8^*, Yanjie Liang^1,2^*

^1^School of Materials Science & Engineering, Shandong University, Jinan 250061, China

^2^State Key Laboratory of Coatings for Advanced Equipment, Shandong University, Jinan 250061, China

^3^School of Information and Communication Engineering, Beijing University of Posts and Telecommunications, Beijing 100876, China

^4^Shandong Laboratory of Advanced Materials and Green Manufacturing at Yantai, Yantai 264006, China

^5^State Key Laboratory of Solid Lubrication, Lanzhou Institute of Chemical Physics, Chinese Academy of Sciences, Lanzhou 730000, China

^6^Department of Oral and Maxillofacial Surgery, School and Hospital of Stomatology, Cheeloo College of Medicine, Shandong University, Jinan 250012, China

^7^Department of Oral and Maxillofacial Surgery, Qilu Hospital of Shandong University, Jinan 250012, China

^8^Department of Physics, Georgia Southern University, Statesboro, GA 30460, USA

Correspondence: Yanjie Liang (yanjie.liang@sdu.edu.cn)

Xiao-Jun Wang [(xwang@georgiasouthern.edu)](mailto:(xwang@georgiasouthern.edu))

[Zhaofeng Wang (zhfwang@licp.cas.cn)](mailto:(xwang@georgiasouthern.edu))


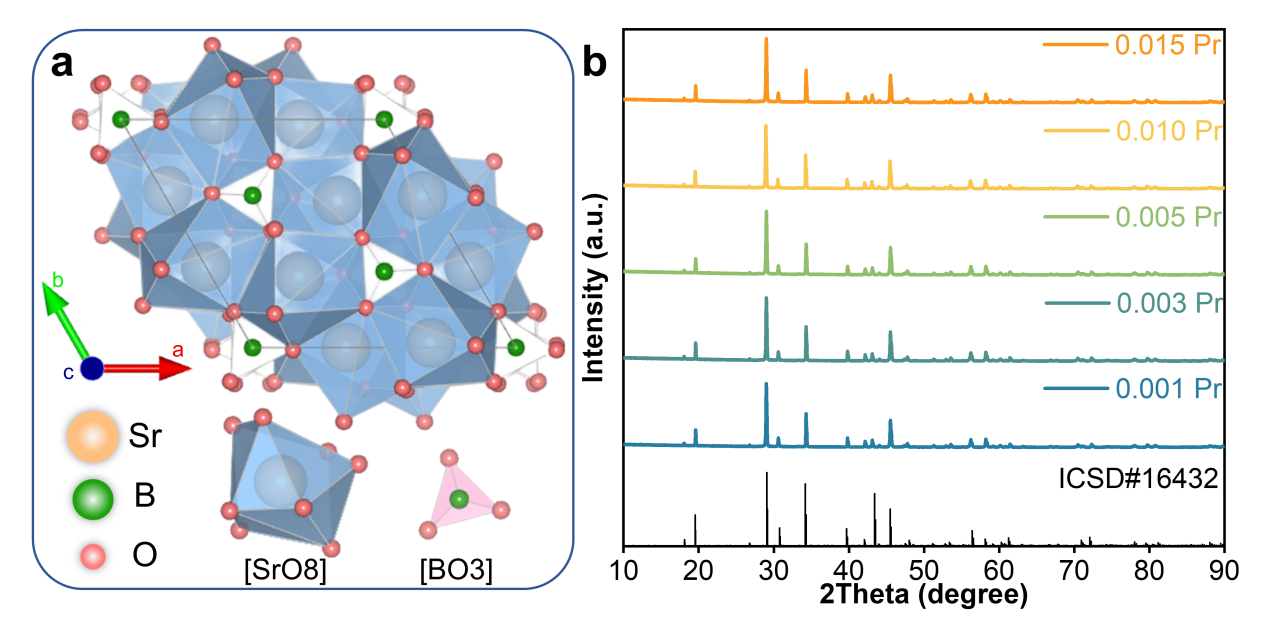


**Fig. S1** **(a)** A schematic diagram of the crystal structure of Sr_3_(BO_3_)_2_ host. **(b)** XRD patterns of SBO:*x*Pr^3+^ phosphors (0<*x*≤0.015).


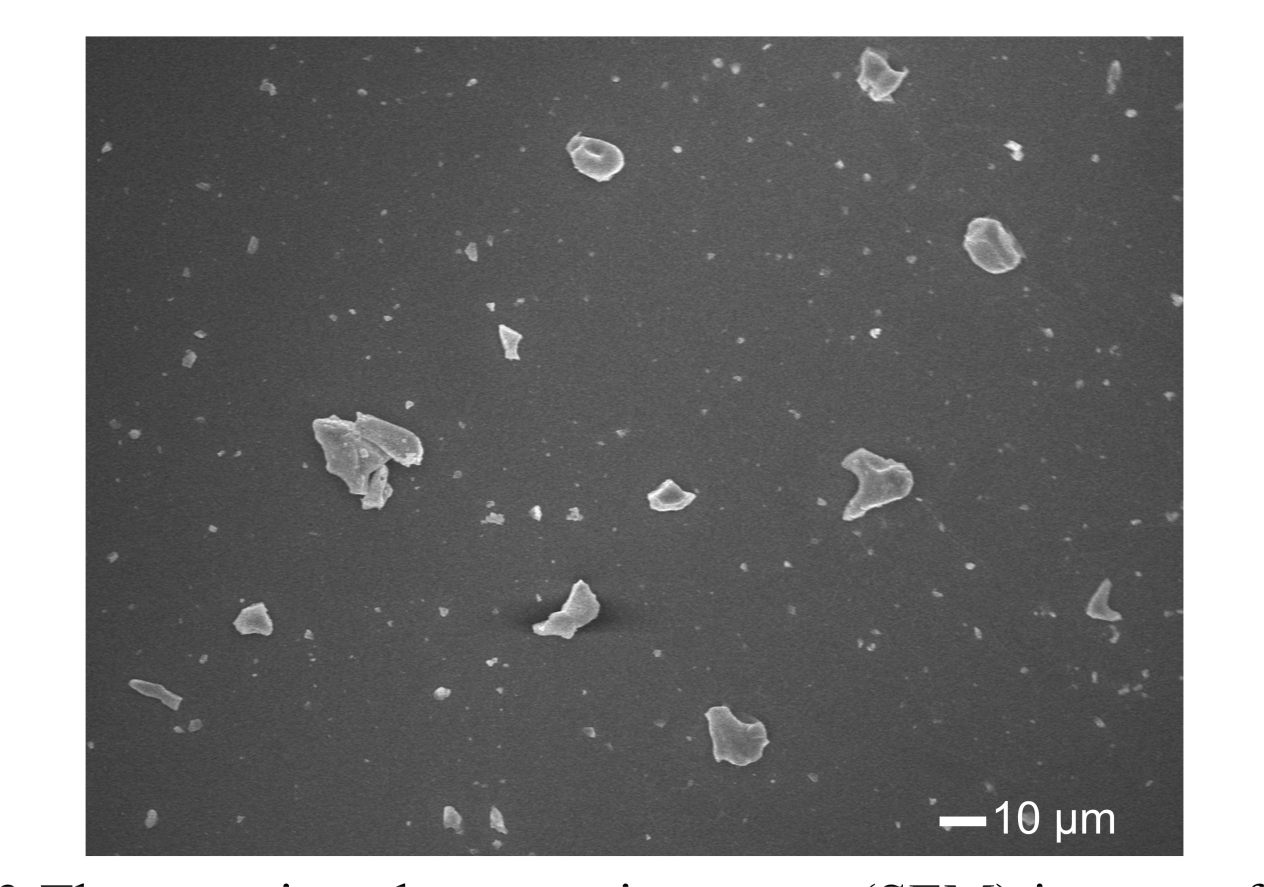


**Fig. S2** Scanning electron microscope (SEM) images of the SBO:Pr phosphor.


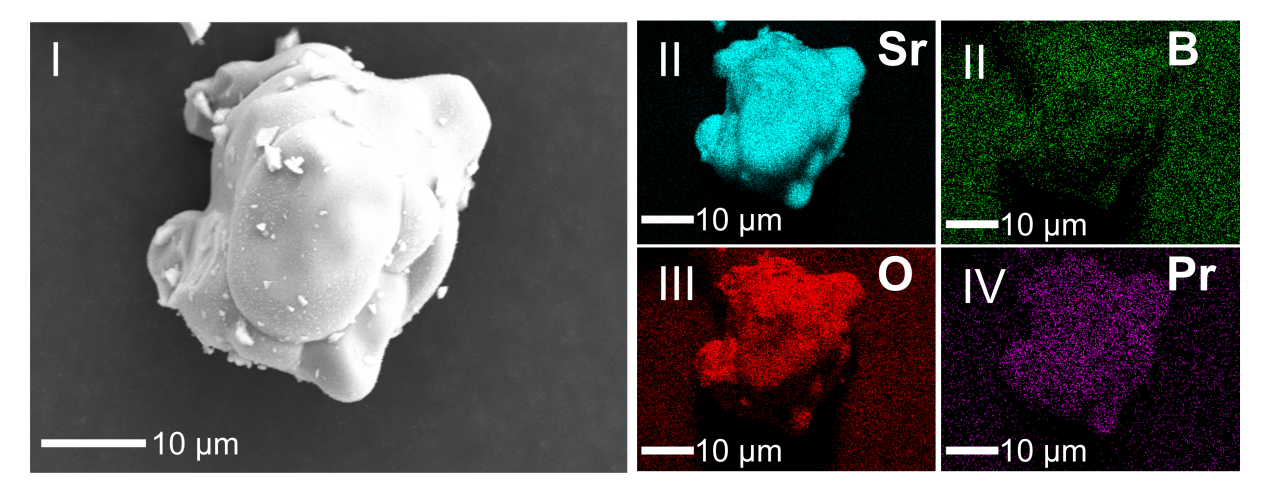


**Fig. S3** Energy-dispersive spectroscopy (EDS) mapping of the SBO:Pr phosphor.


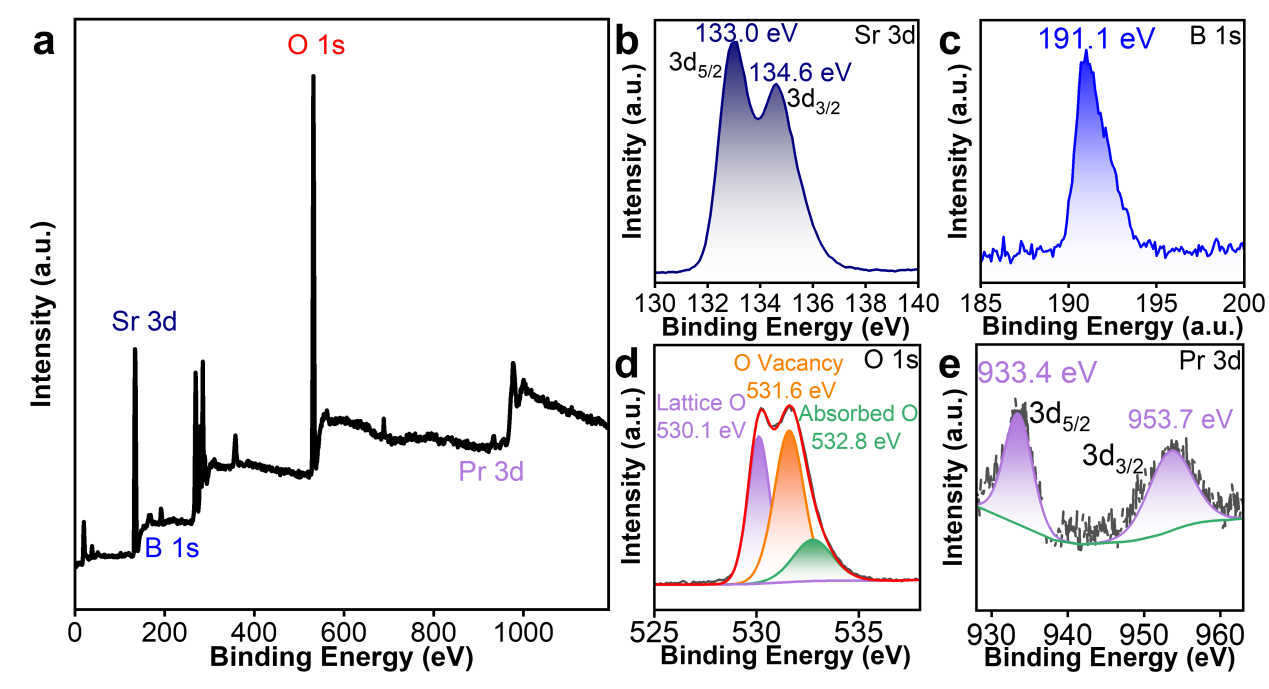


**Fig. S4** X-ray photoelectron spectroscopy (XPS) analysis of the SBO:Pr phosphor. **(a)** Full spectrum of XPS spectrum and enlarged XPS spectra of **(b)** Sr 3d, **(c)** B1s, **(d)** O1s, and **(e)** Pr3d of the SBO:Pr phosphor.


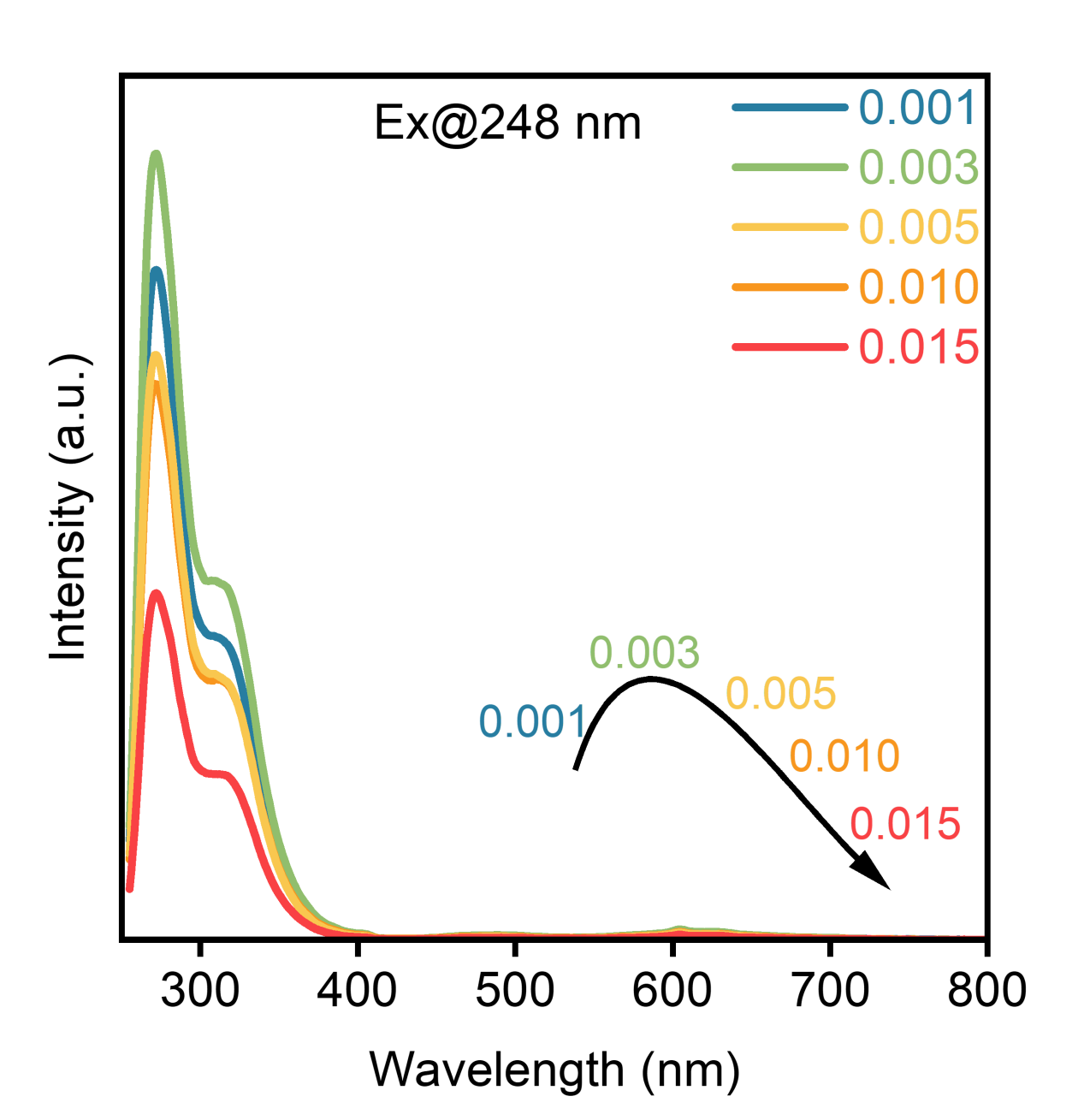


**Fig. S5** PL emission spectra of the SBO:*x*Pr phosphors.


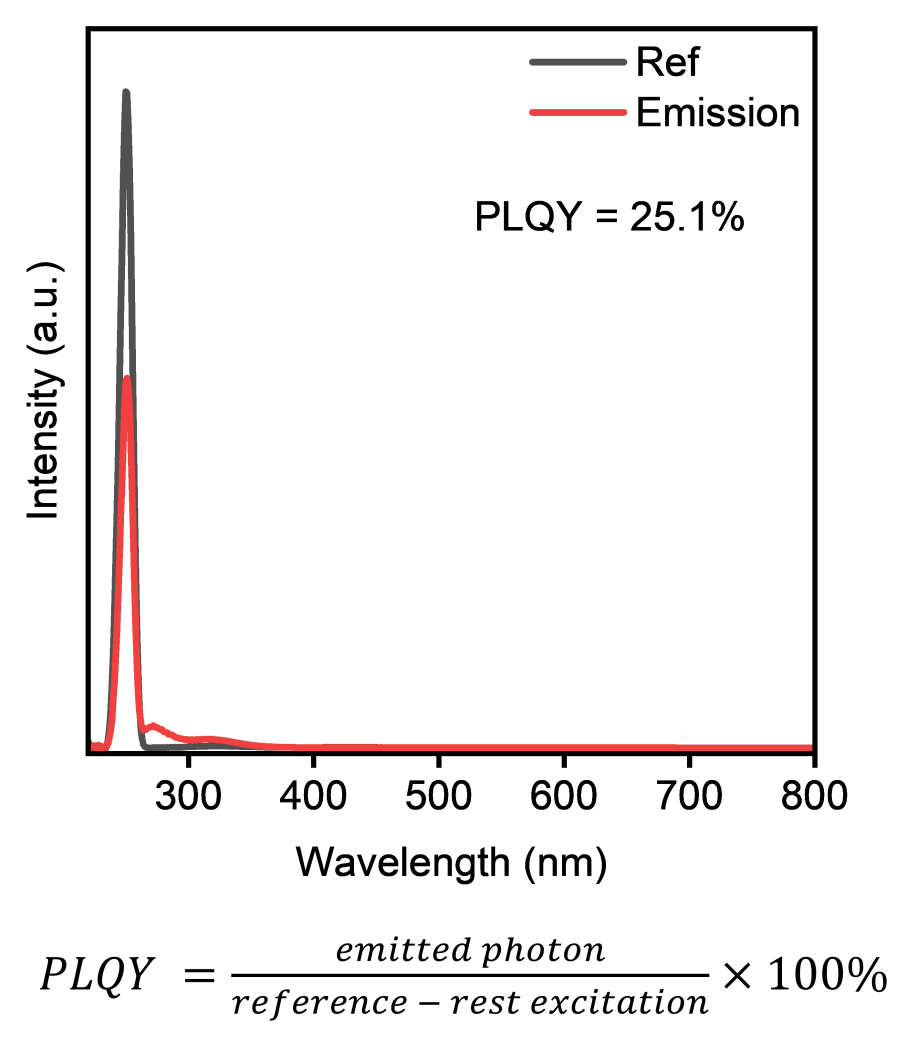


**Fig. S6** Photoluminescence quantum yield (PLQY) measurement of the SBO:Pr phosphor upon 250 nm excitation.


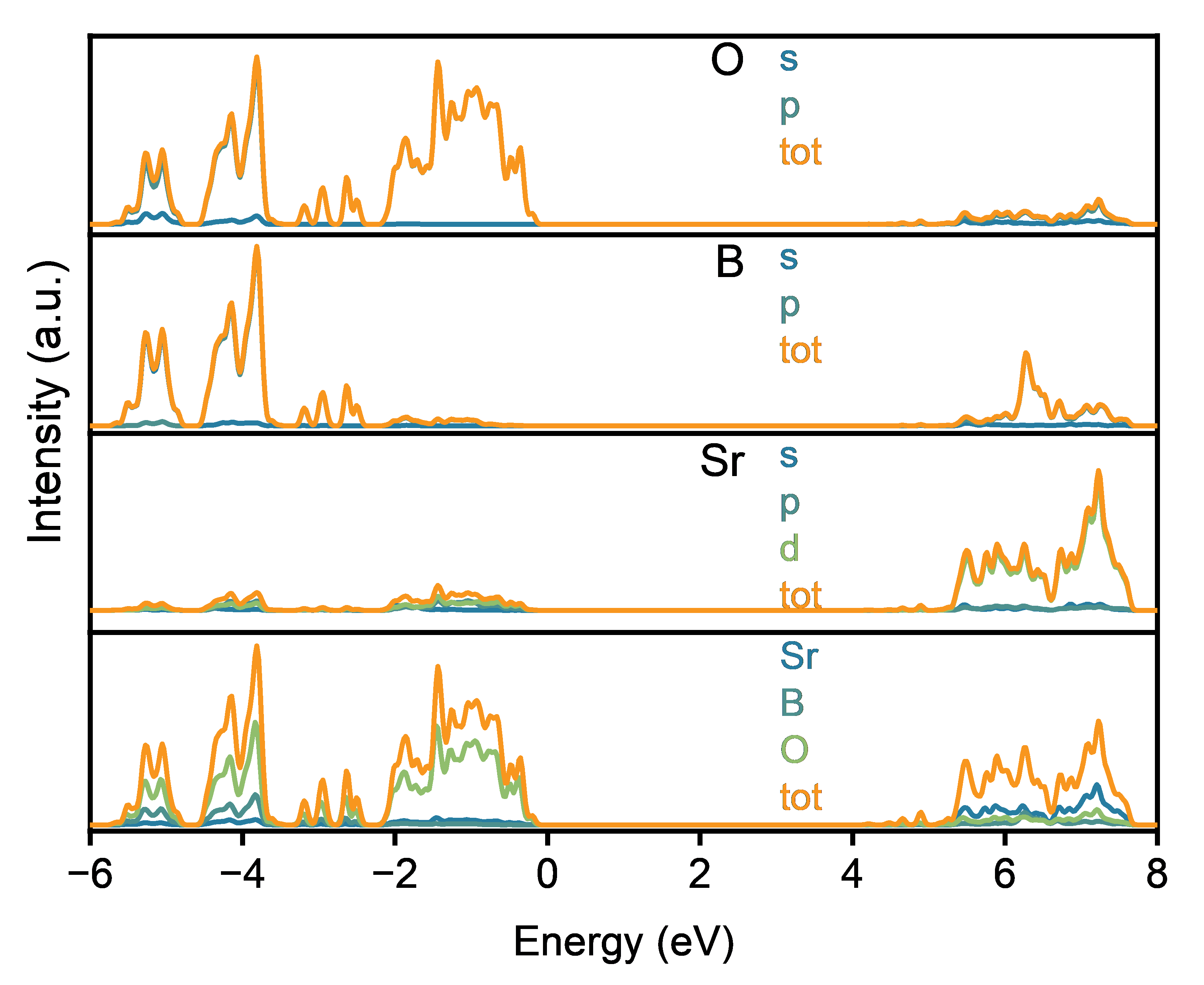


**Fig. S7** The densities of states (DOS) of the atoms within the Sr_3_(BO_3_)_2_ host.


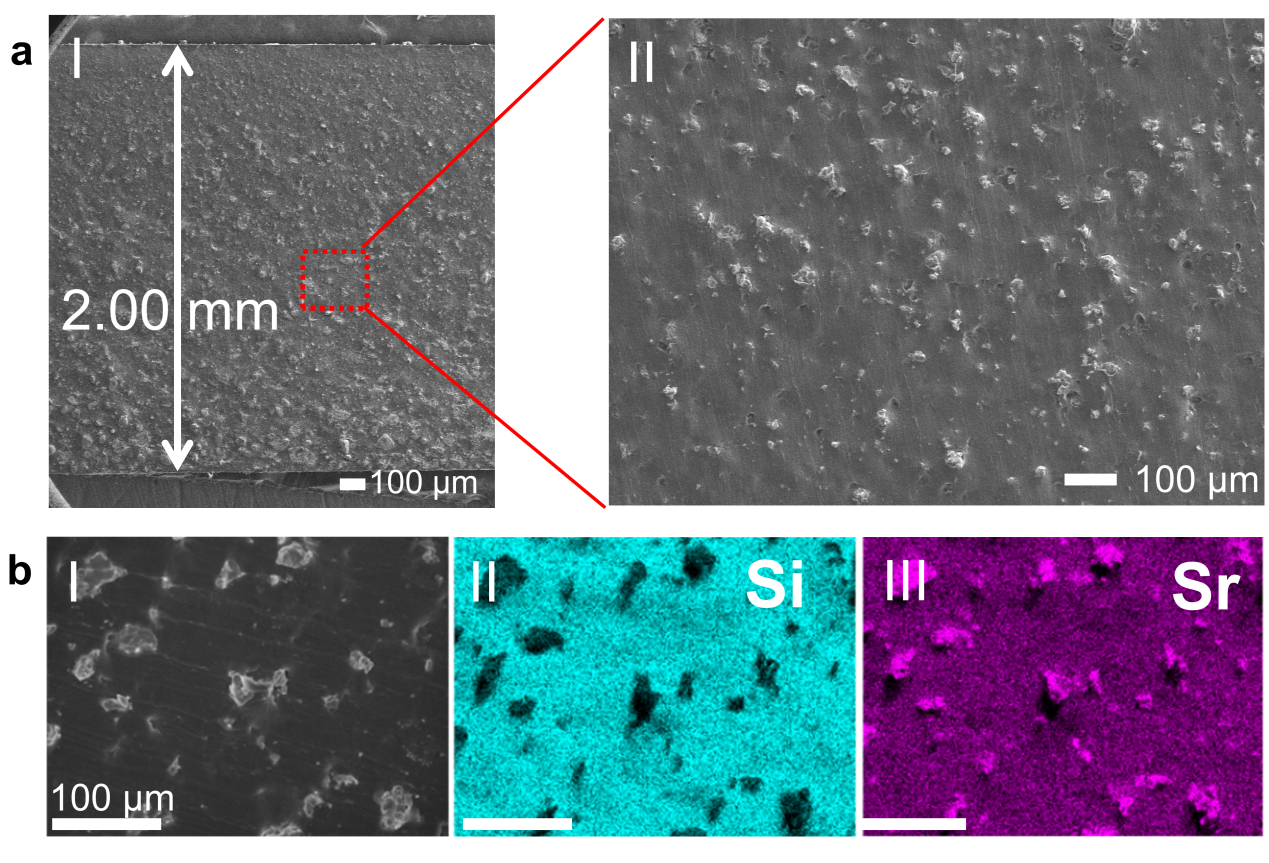


**Fig. S8 (a)** The cross-sectional scanning electron microscope (SEM) images of the SBO:Pr/PDMS elastomer film. **(b)** The Energy-dispersive spectroscopy (EDS) mapping of the SBO:Pr/PDMS elastomer film.


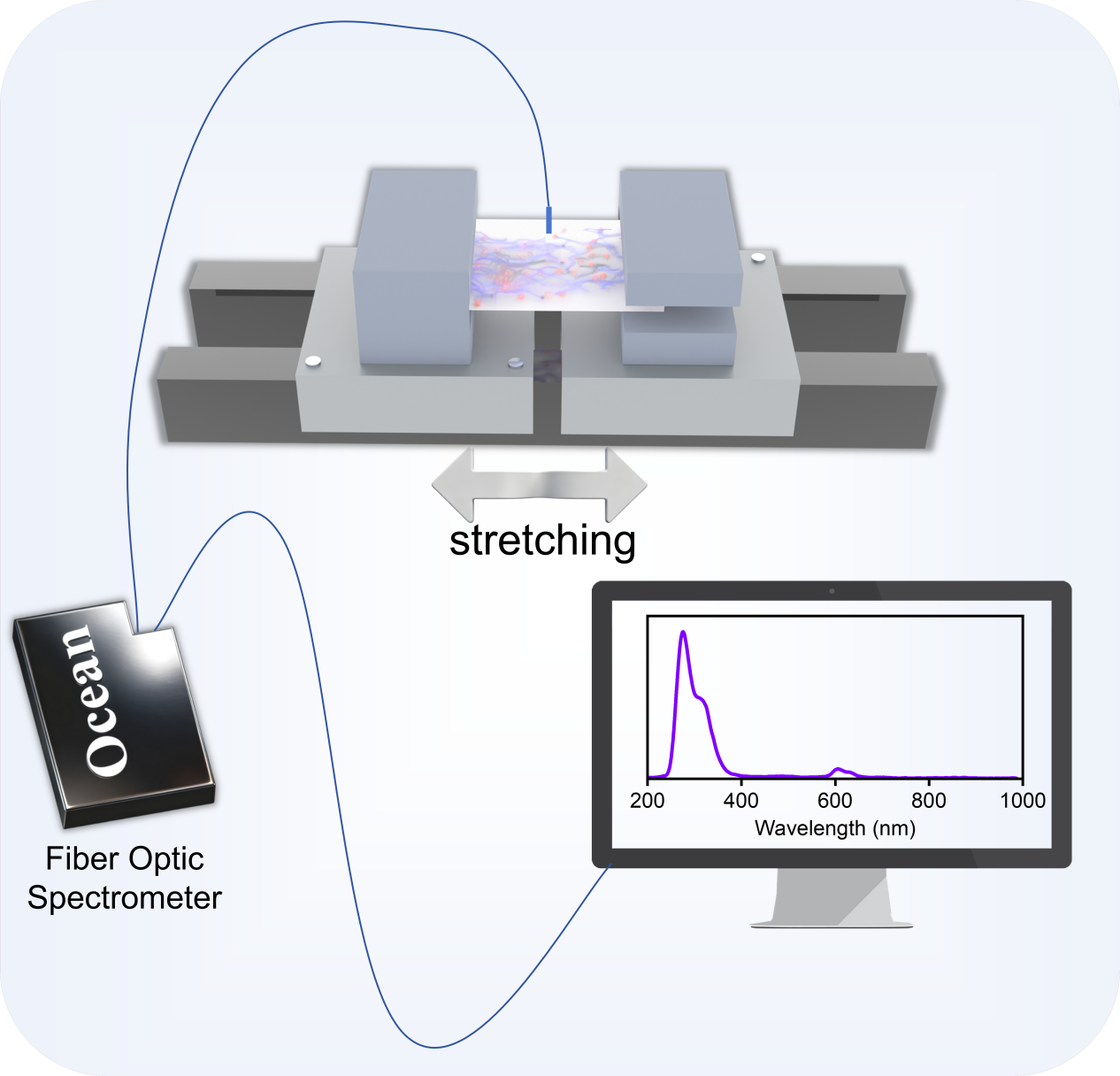


**Fig. S9** The schematic diagram of the stretching device.


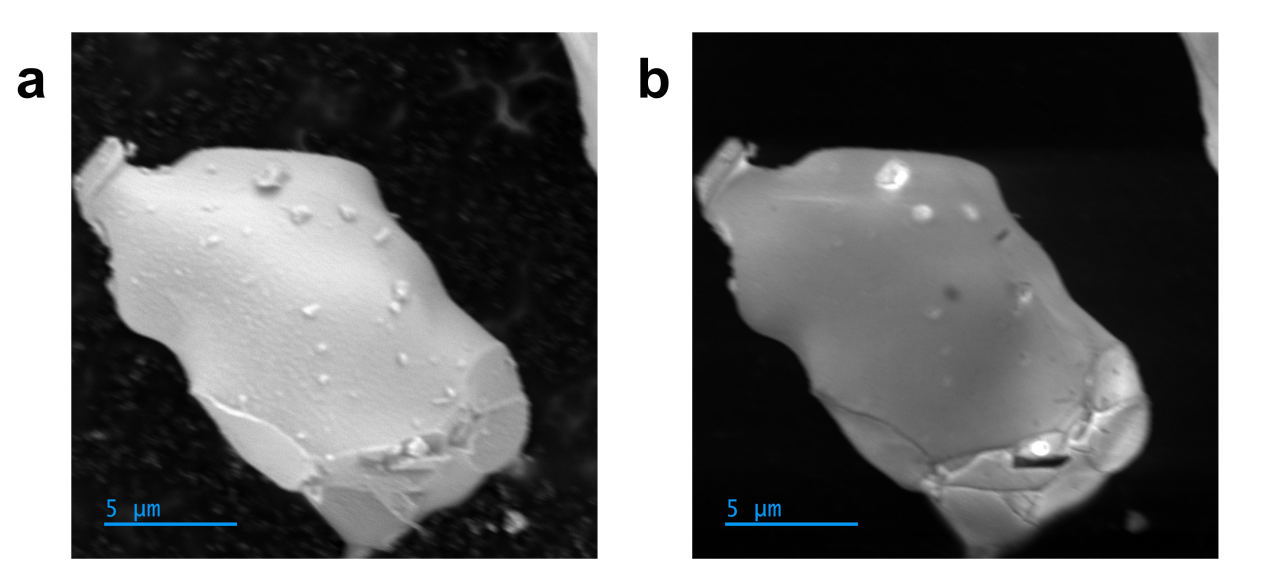


**Fig. S10** **(a)** SEM image and **(b)** CL image of the SBO:Pr phosphor particle.


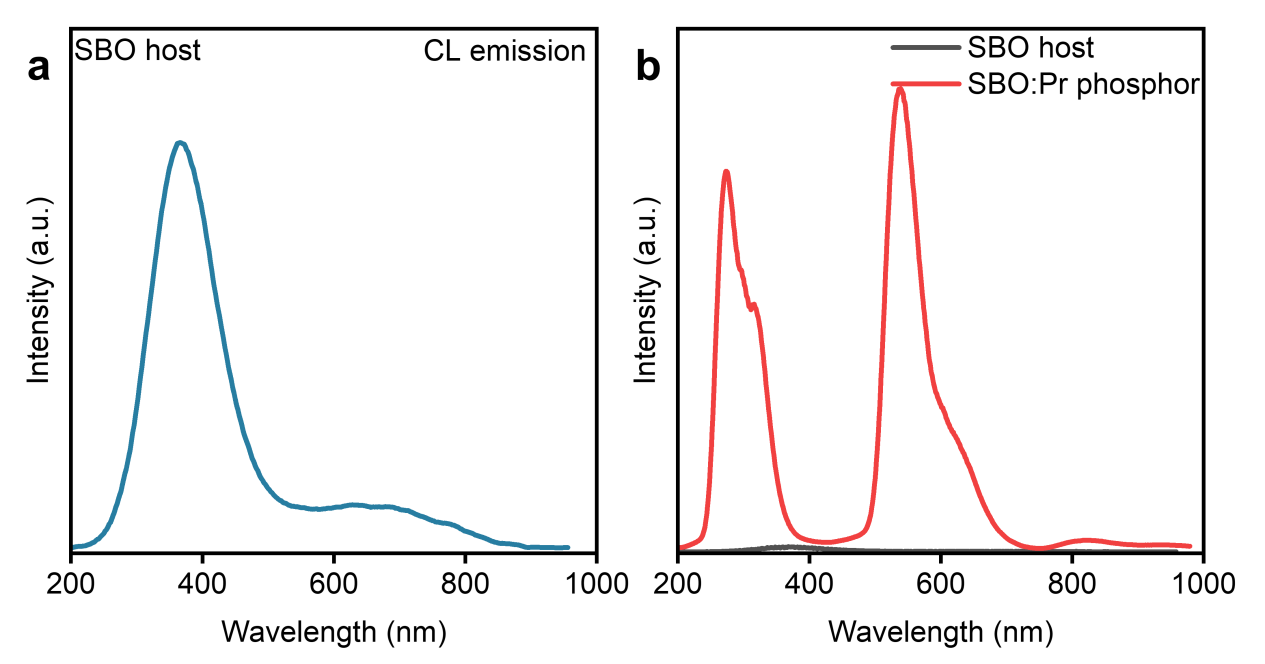


**Fig. S11** CL emission spectrum of **(a)** the SBO host and **(b)** SBO:Pr phosphor.


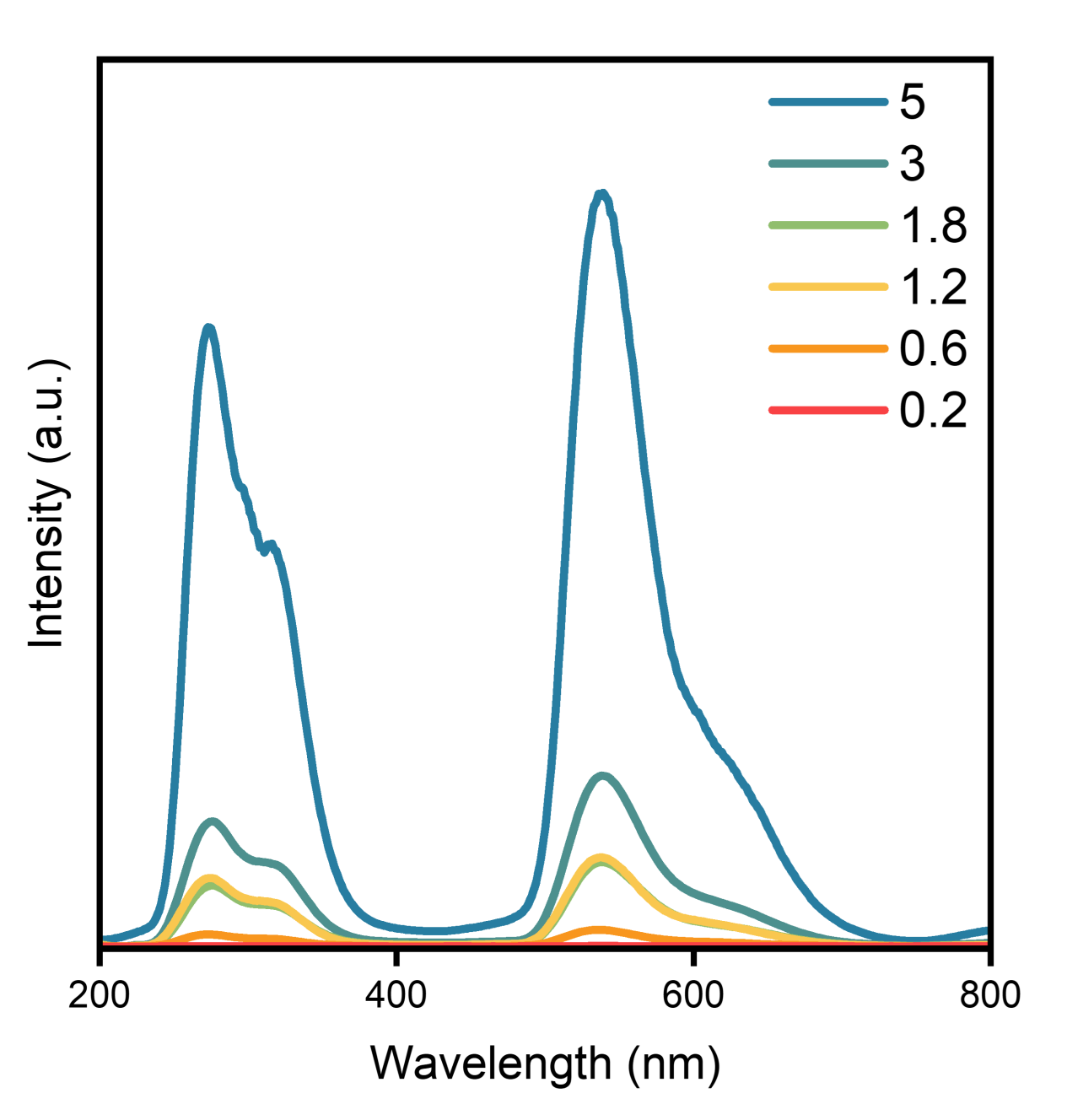


**Fig. S12** CL emission spectra of the SBO:Pr phosphor under different voltage (kV).


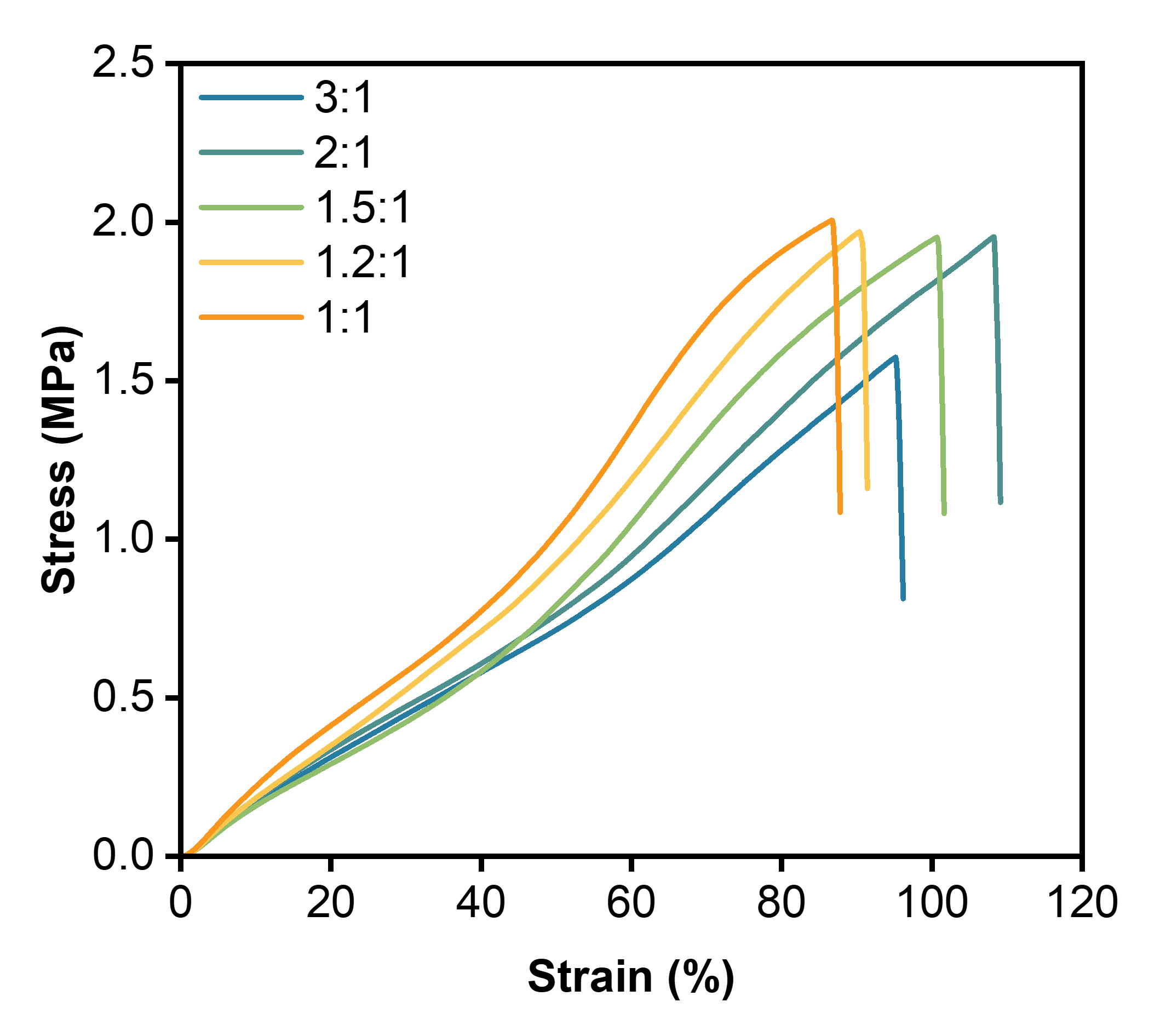


**Fig. S13** The stress-strain curves of SBO:Pr/PDMS elastomer film with different mass ratio.


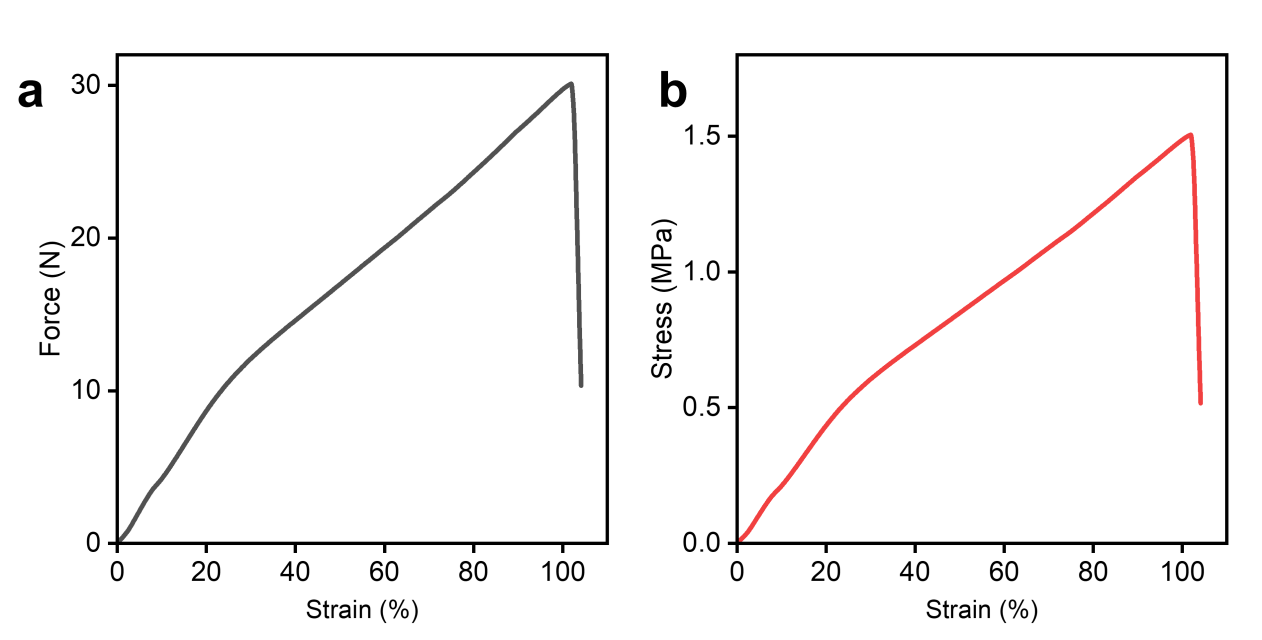


**Fig. S14** **(a)** Force-strain and **(b)** stress-strain curve of the SBO:Pr/PDMS elastomer film.


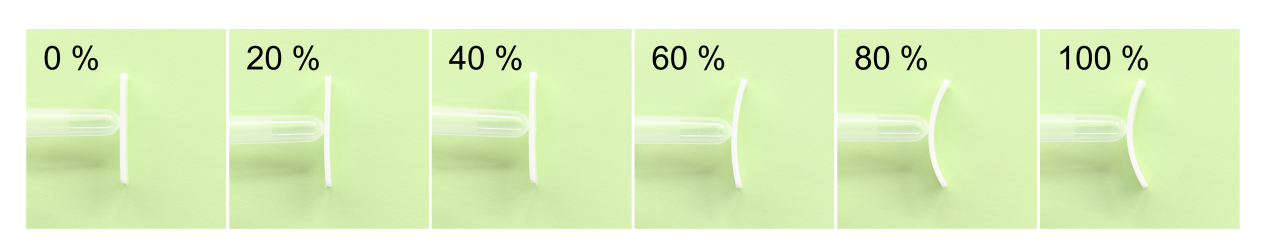


**Fig. S15** The side-view images of the SBO:Pr/PDMS elastomer film after varied stretching strains.


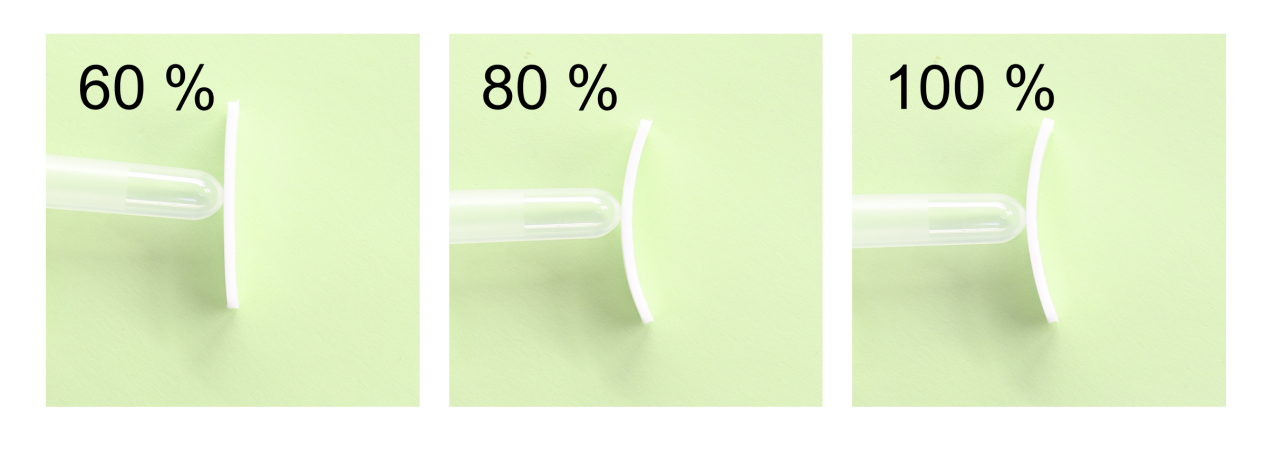


**Fig. S16** The side-view images of the SBO:Pr/PDMS elastomer film after being naturally placed at room temperature for 24 h.


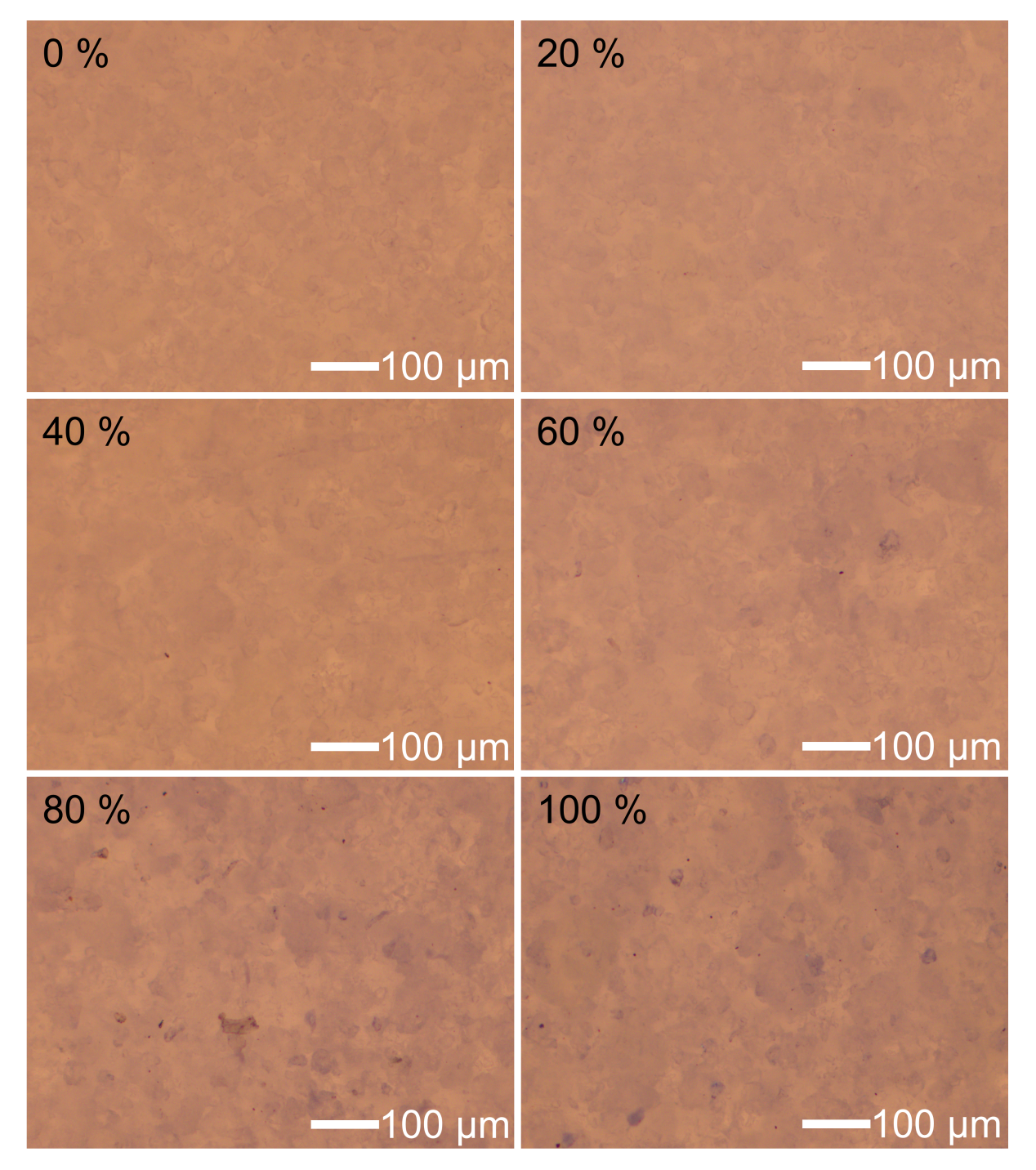


**Fig. S17** The color optical micrographs of the surface morphology of the SBO:Pr/PDMS elastomer film after varied stretching strains.


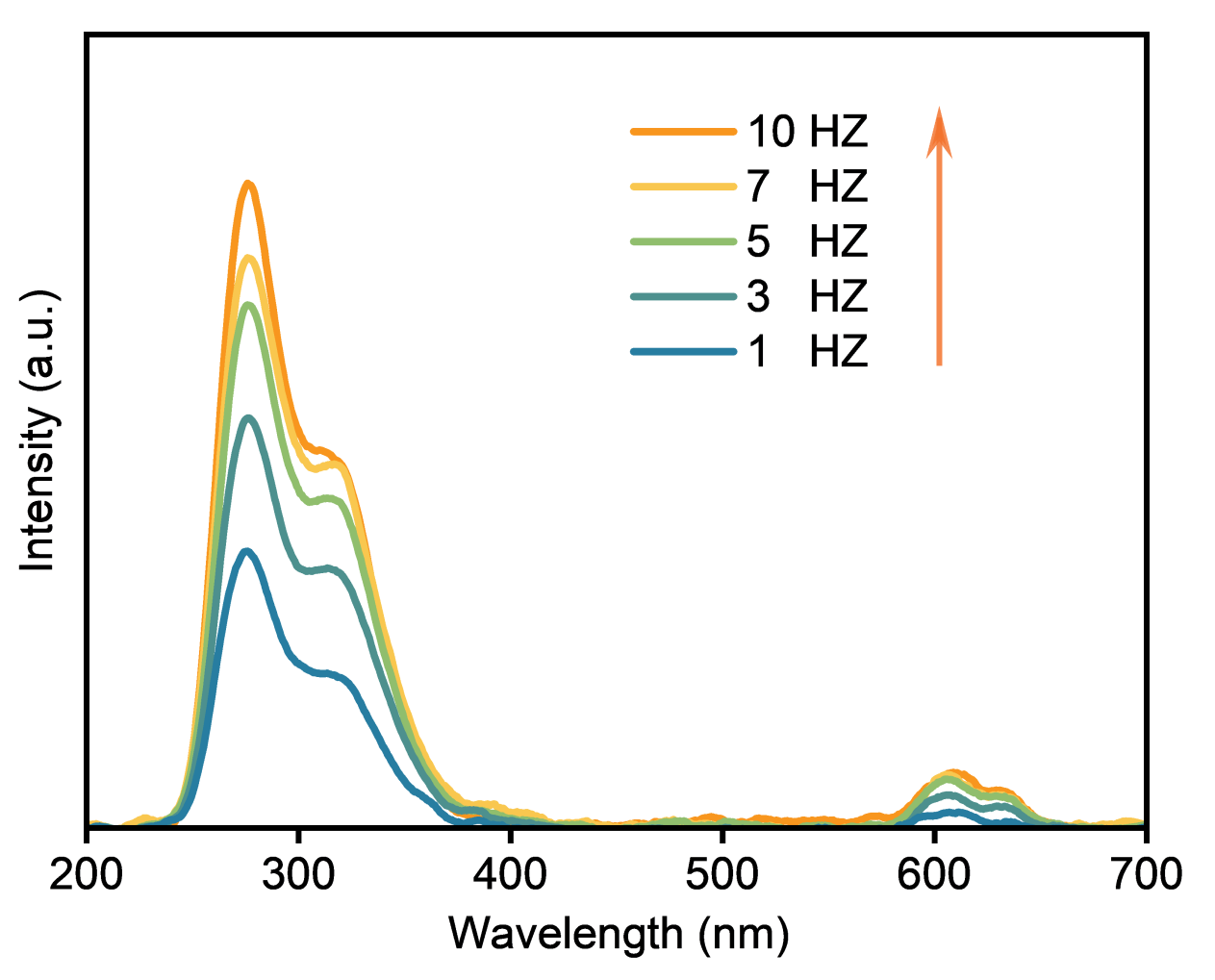


**Fig. S18** The ML emission spectra of the SBO:Pr/PDMS elastomer film under various stretching frequency.


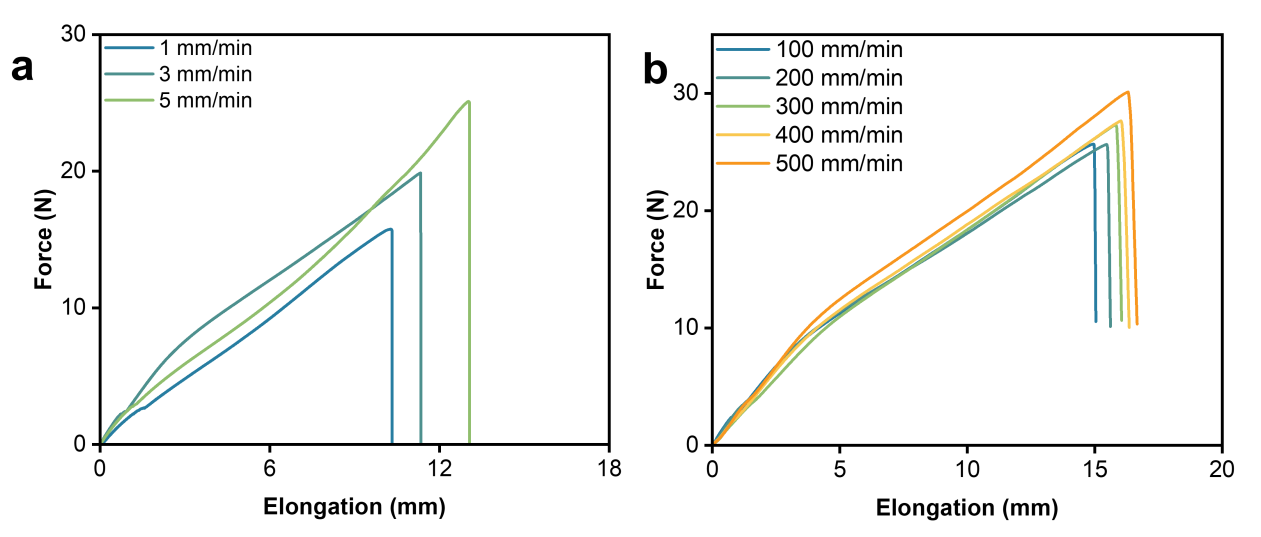


**Fig. S19** The force-elongation curves of the SBO:Pr/PDMS elastomer film under various stretching rate: **(a)** low stretching rate and **(b)** high stretching rate.


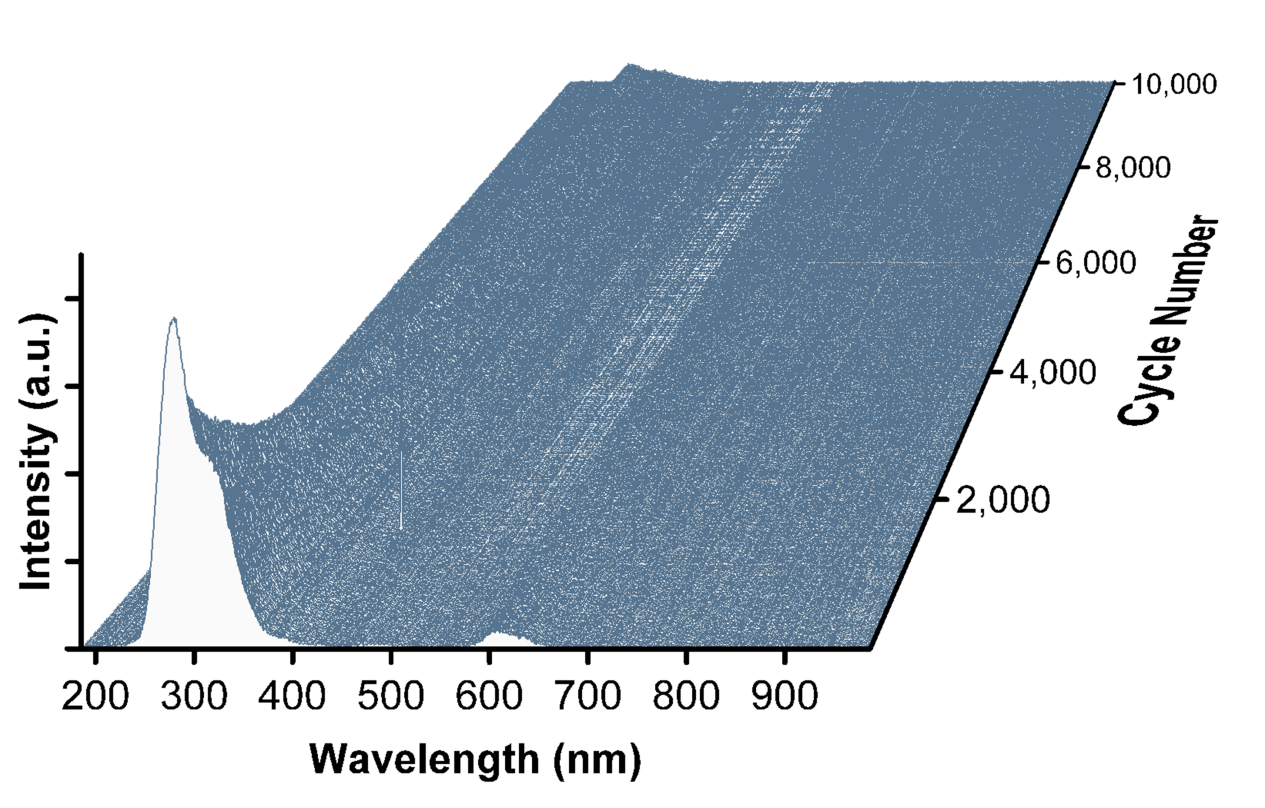


**Fig. S20** The ML emission spectra of the SBO:Pr/PDMS elastomer film under 10,000 stretching cycles (frequency: 5 HZ; strain: 40%).


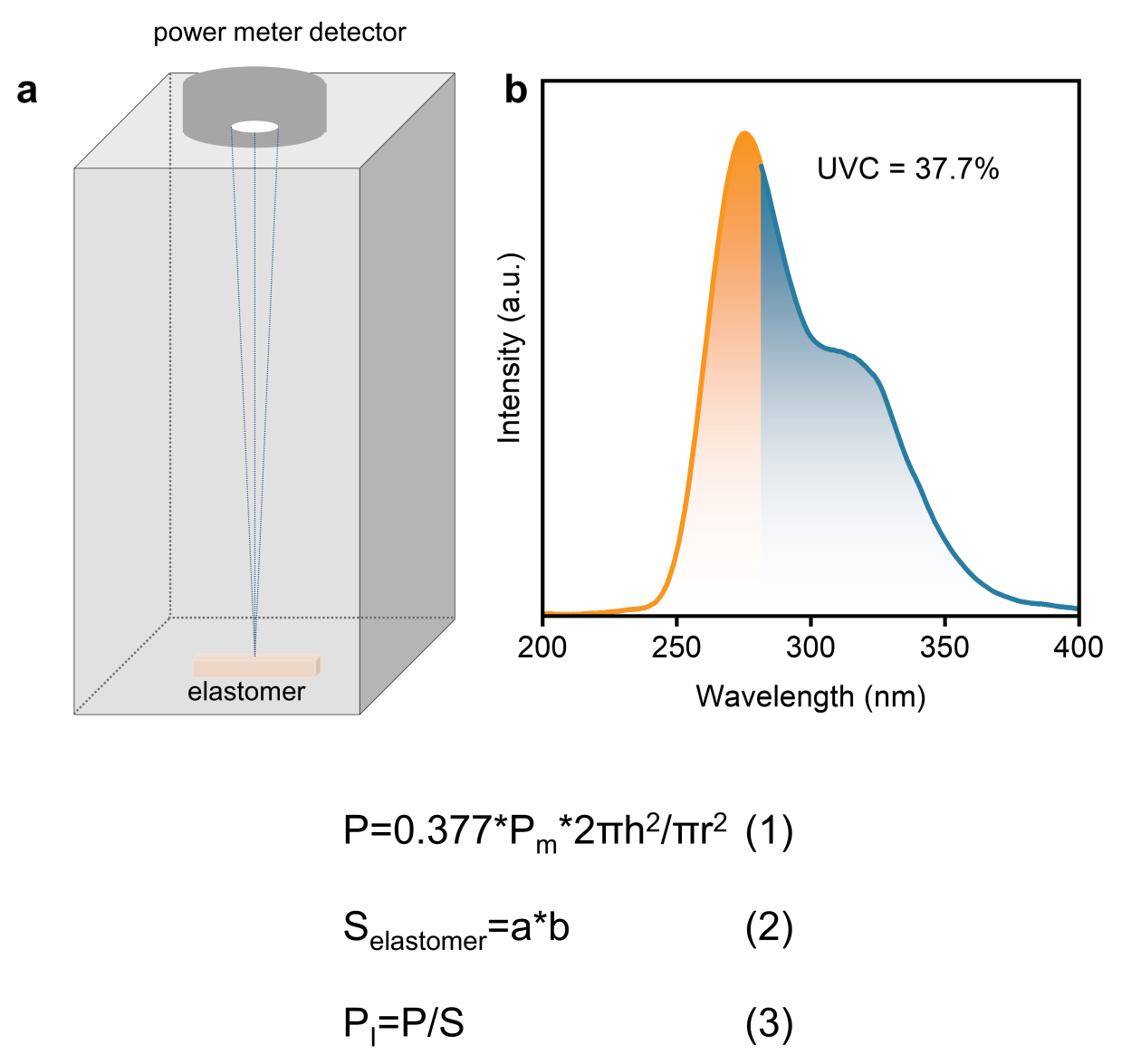


**Fig. S21** **(a)** The schematic illustration of the SBO:Pr/PDMS elastomer power measurement. **(b)** The ML emission of the SBO:Pr/PDMS elastomer and the calculation formula.

Besides UVC emission at 240-280 nm (region I), the spectrum also contains emission in the 280-400 nm range (region II). We integrated the areas of region I and region II and found that region I accounted for 37.7% of the whole emission area. Therefore, to calculate the UVC ML power intensity, the power measured by the UV photodetector needs to be multiplied by 0.377.

We assumed the UVC ML signal was hemispherical radiation and the intensity at each position of the elastomer was the same as that at the center position. We calculated the UVC ML power using equation (1), where *P_m_* is the measured power by the power meter, *h* is the distance between the photodetector and the elastomer, which is 0.2 m, and *r* is the radius of sensor, which is 0.005 m. The UVC ML power intensity (*P_I_*) was then obtained by dividing the power (*P*) with the area of elastomer (S=a*b=0.02*0.01=0.0002 m^2^ ).


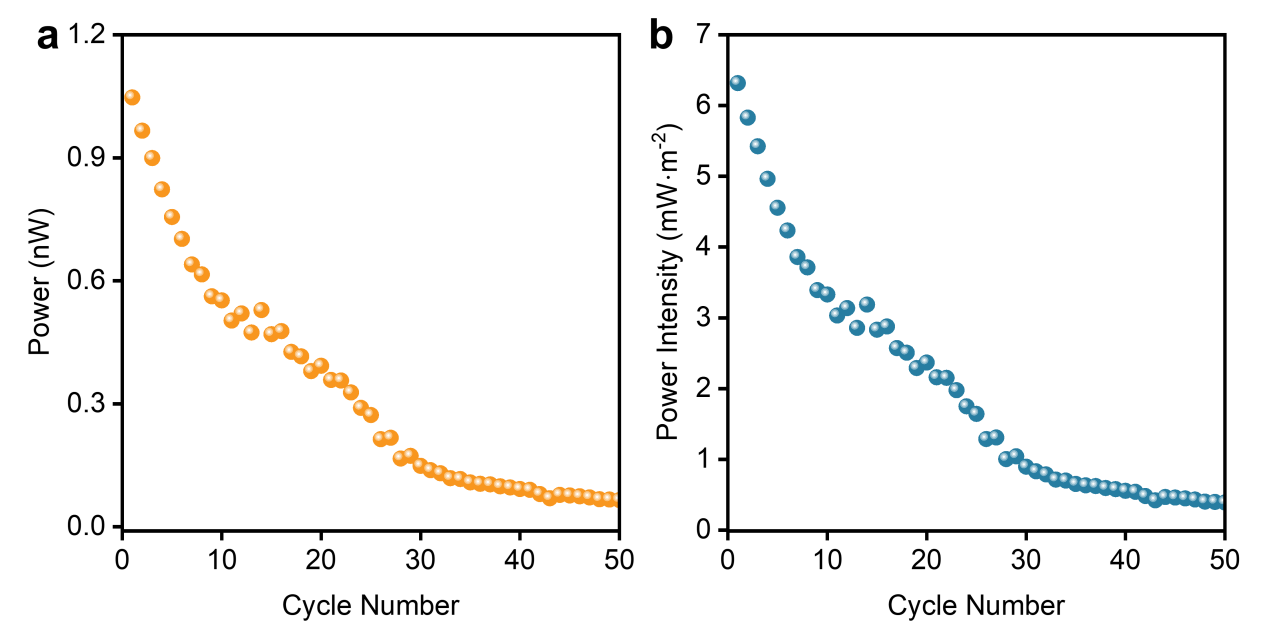


**Fig. S22** The **(a)** power and **(b)** power intensity of the SBO:Pr/PDMS elastomer.


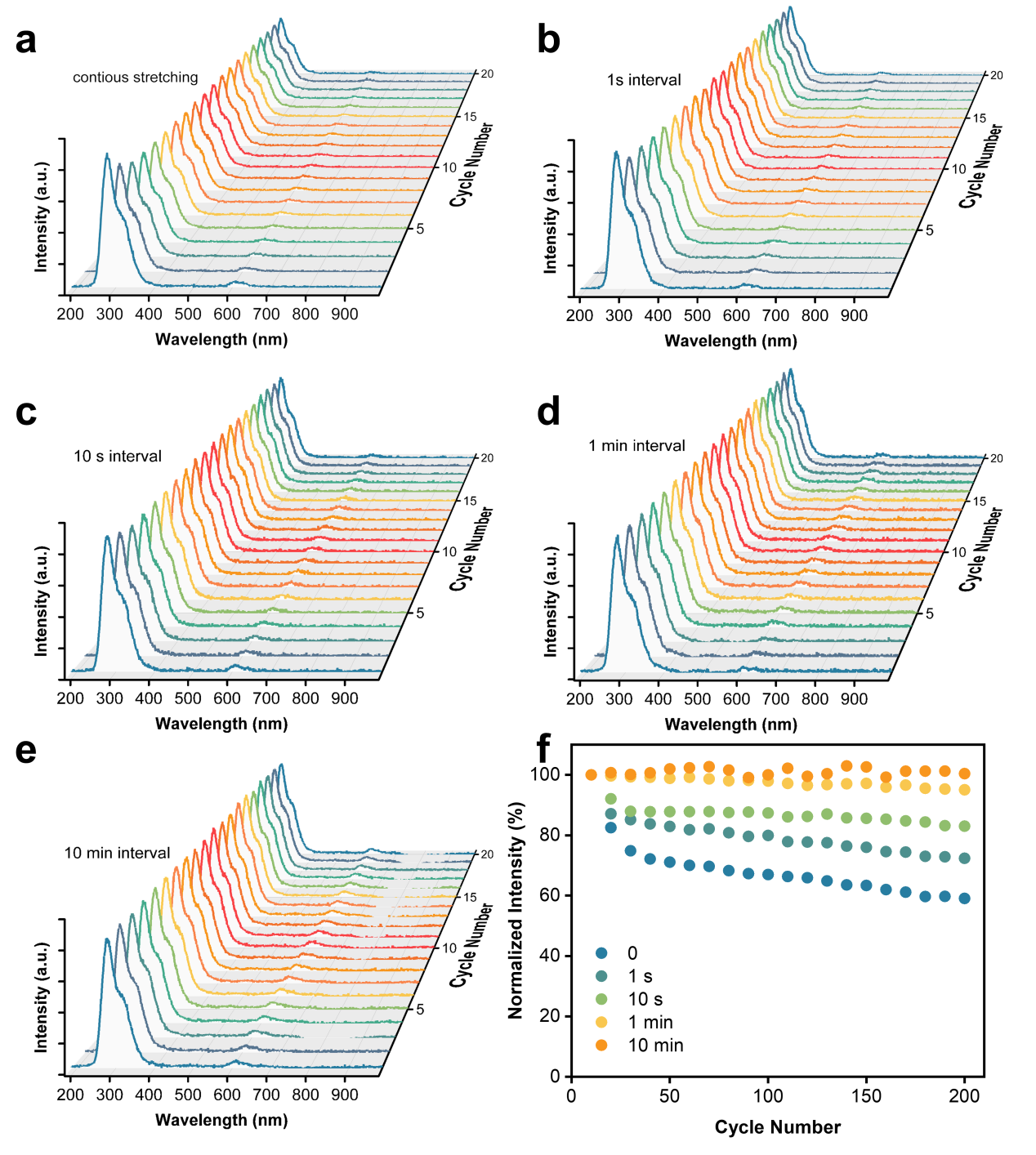


**Fig. S23** Repeatability test of the ML of SBO:Pr/PDMS elastomer film with various time intervals. ML emission of SBO:Pr/PDMS elastomer film within **(a)** 0 s, **(b)** 1 s, **(c)** 10 s, **(d)** 1 min and **(e)** 10 min interval. **(f)** Corresponding ML intensity evolution of SBO:Pr/PDMS elastomer film with various time intervals.


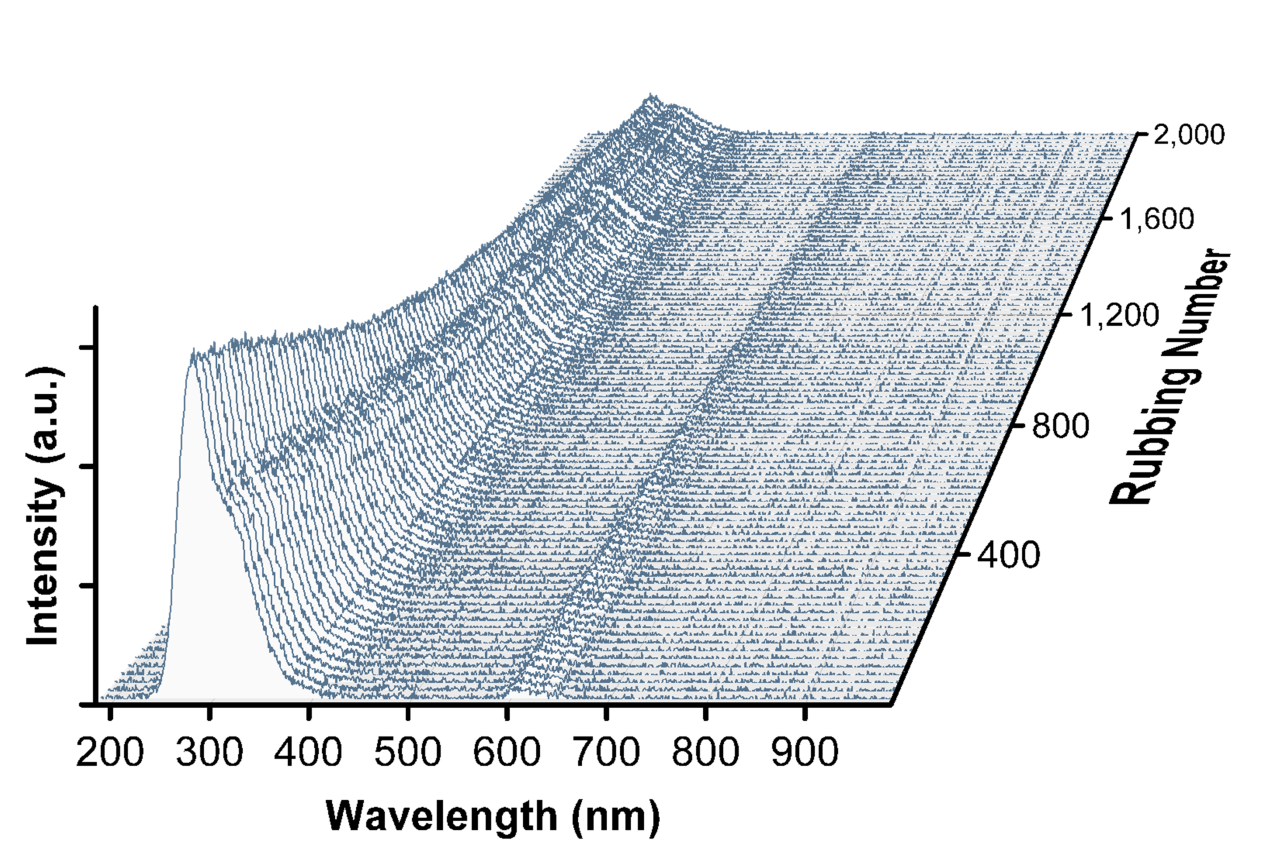


**Fig. S24** The ML emission spectra of the SBO:Pr/PDMS elastomer film under 2,000 rubbing cycles (frequency: 5 HZ; stress: 5 N).


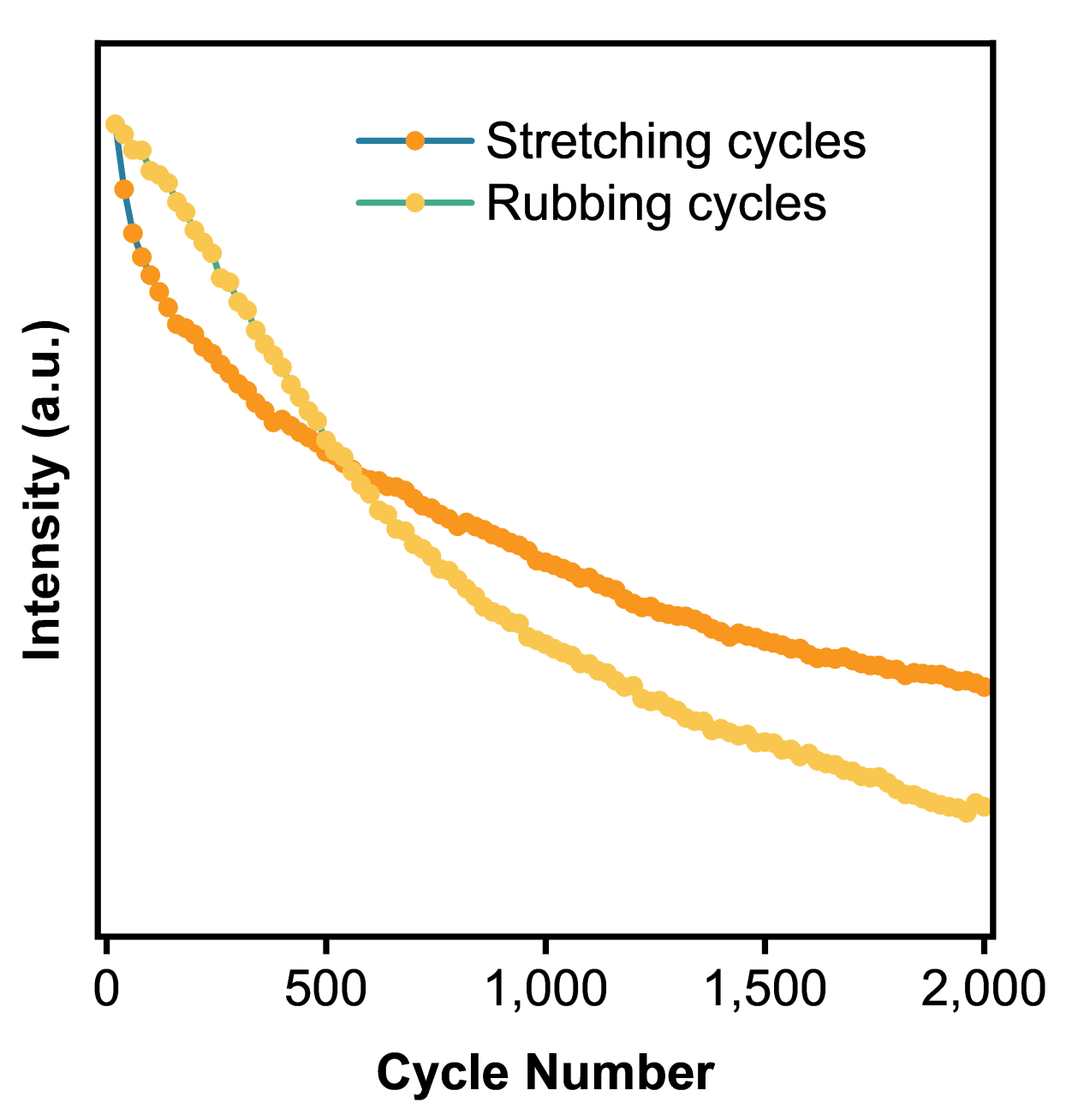


**Fig. S25** The normalized ML intensity evolution of the SBO:Pr/PDMS elastomer film under 2,000 stretching and rubbing cycles.


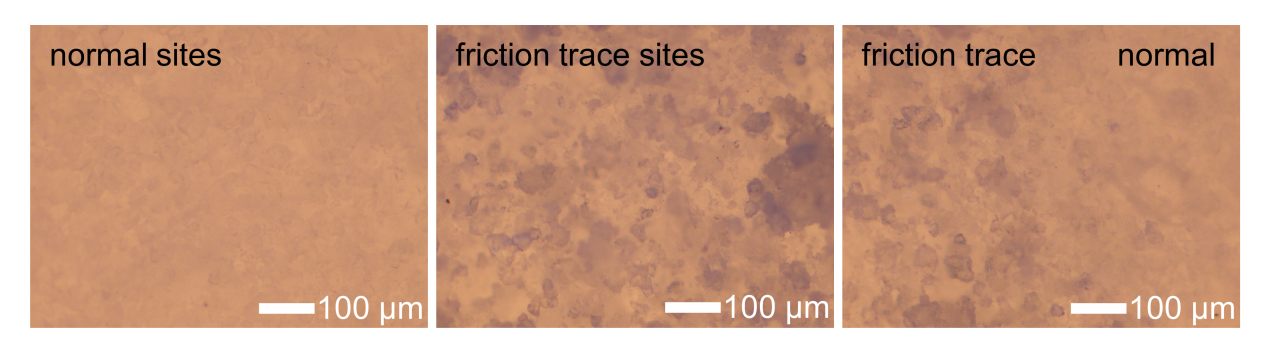


**Fig. S26** The color optical micrographs at surface friction trace sites of the SBO:Pr/PDMS elastomer film.


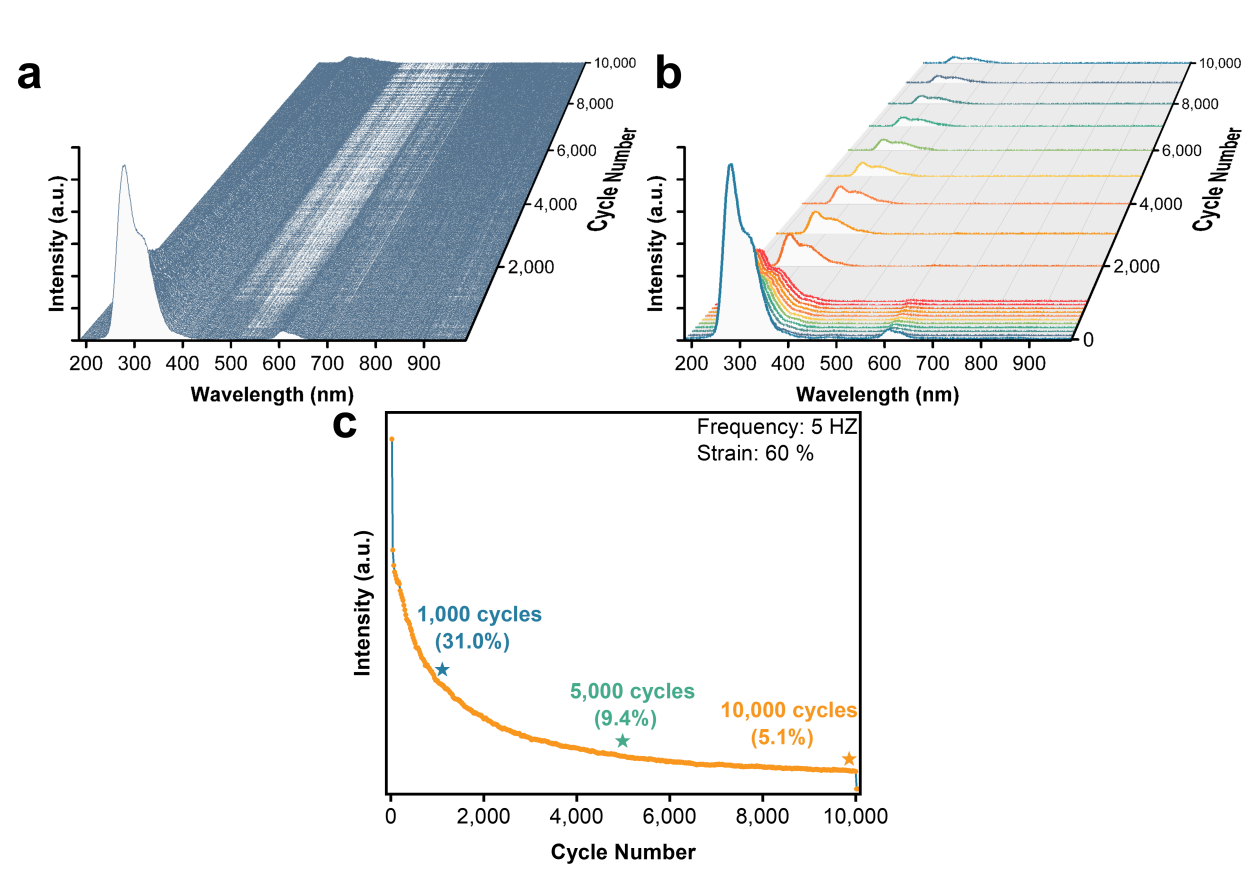


**Fig. S27 (a, b)** ML emission spectra and **(c)** ML intensity evolution over 10,000 stretching cycles of the SBO:Pr/PDMS elastomer film under 60% stretching strain.


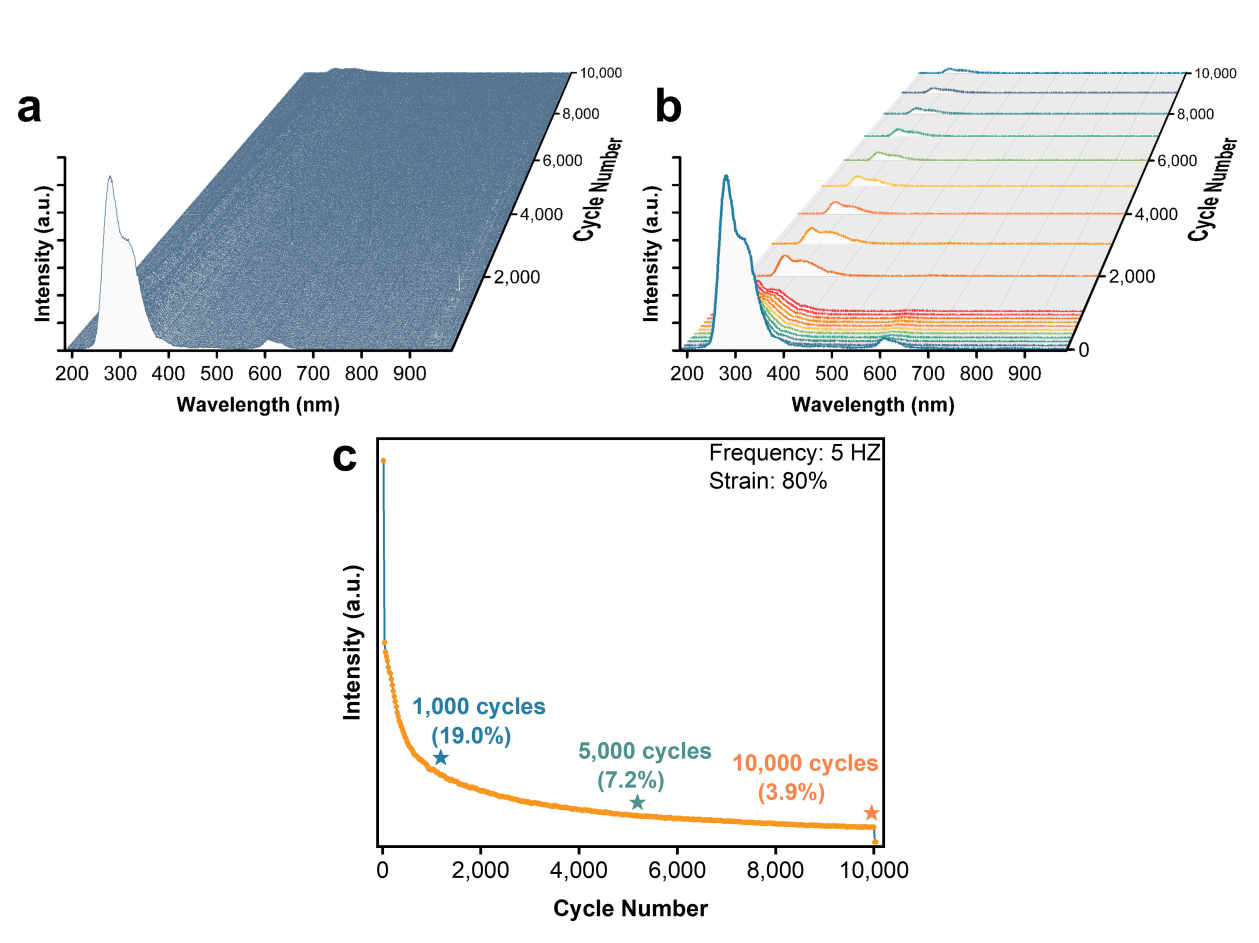


**Fig. S28 (a, b)** ML emission spectra and **(c)** ML intensity evolution over 10,000 stretching cycles of the SBO:Pr/PDMS elastomer film under 80% stretching strain.


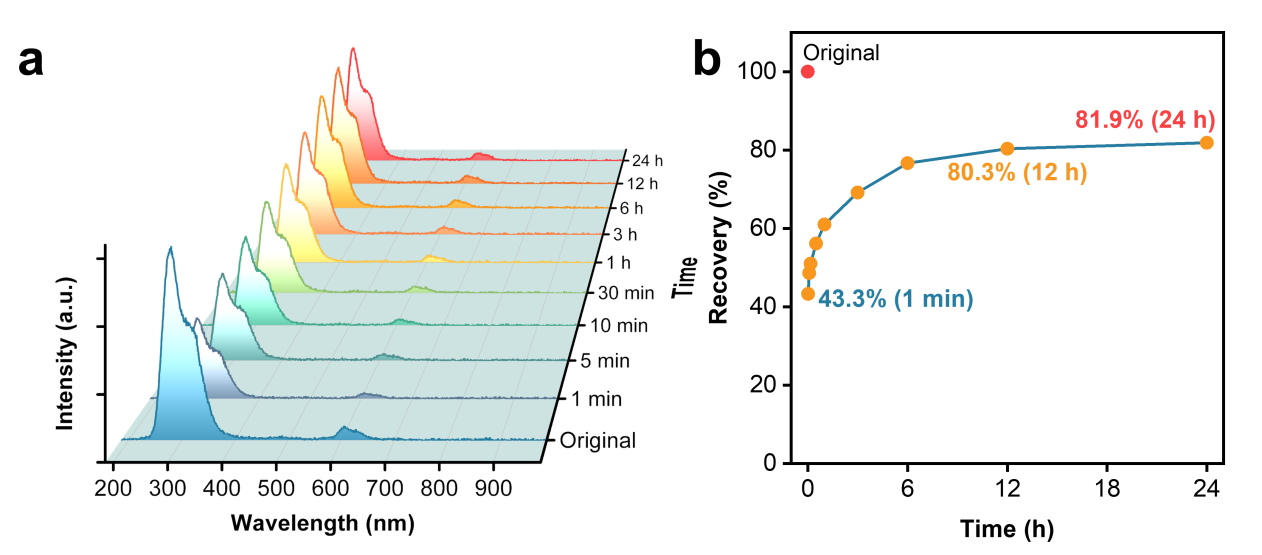


**Fig. S29 (a)** ML emission spectra and **(b)** ML intensity recovery of the SBO:Pr/PDMS elastomer film after natural placing for varied time from 1 min to 24 h at room temperature (frequency: 5 HZ; strain: 60%).


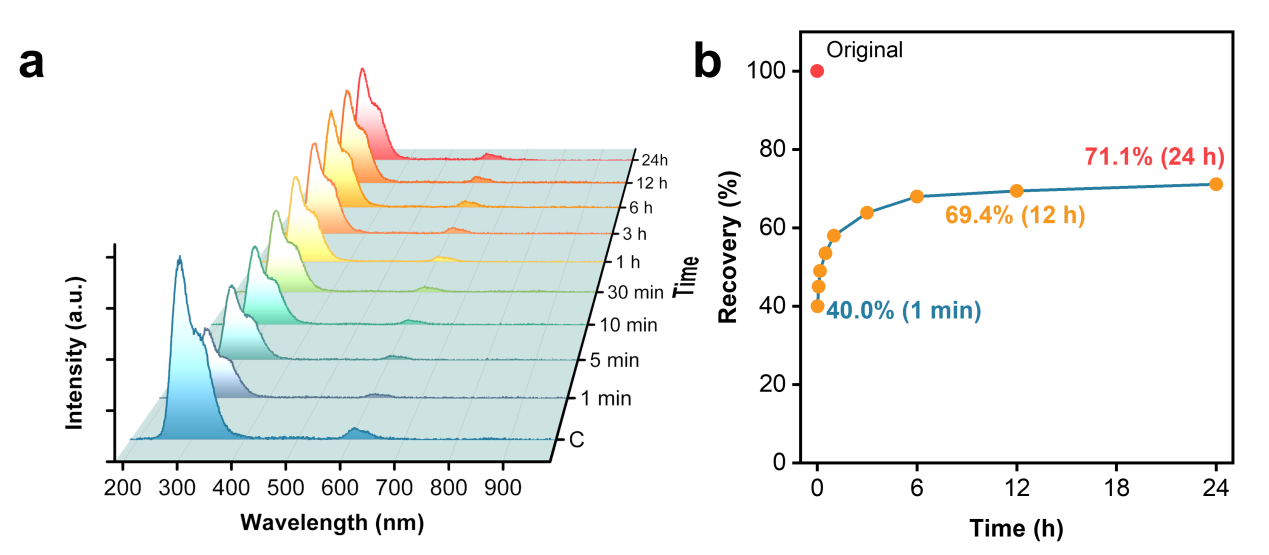


**Fig. S30 (a)** ML emission spectra and **(b)** ML intensity recovery of the SBO:Pr/PDMS elastomer film after natural placing for varied time from 1 min to 24 h at room temperature (frequency: 5 HZ; strain: 80%).


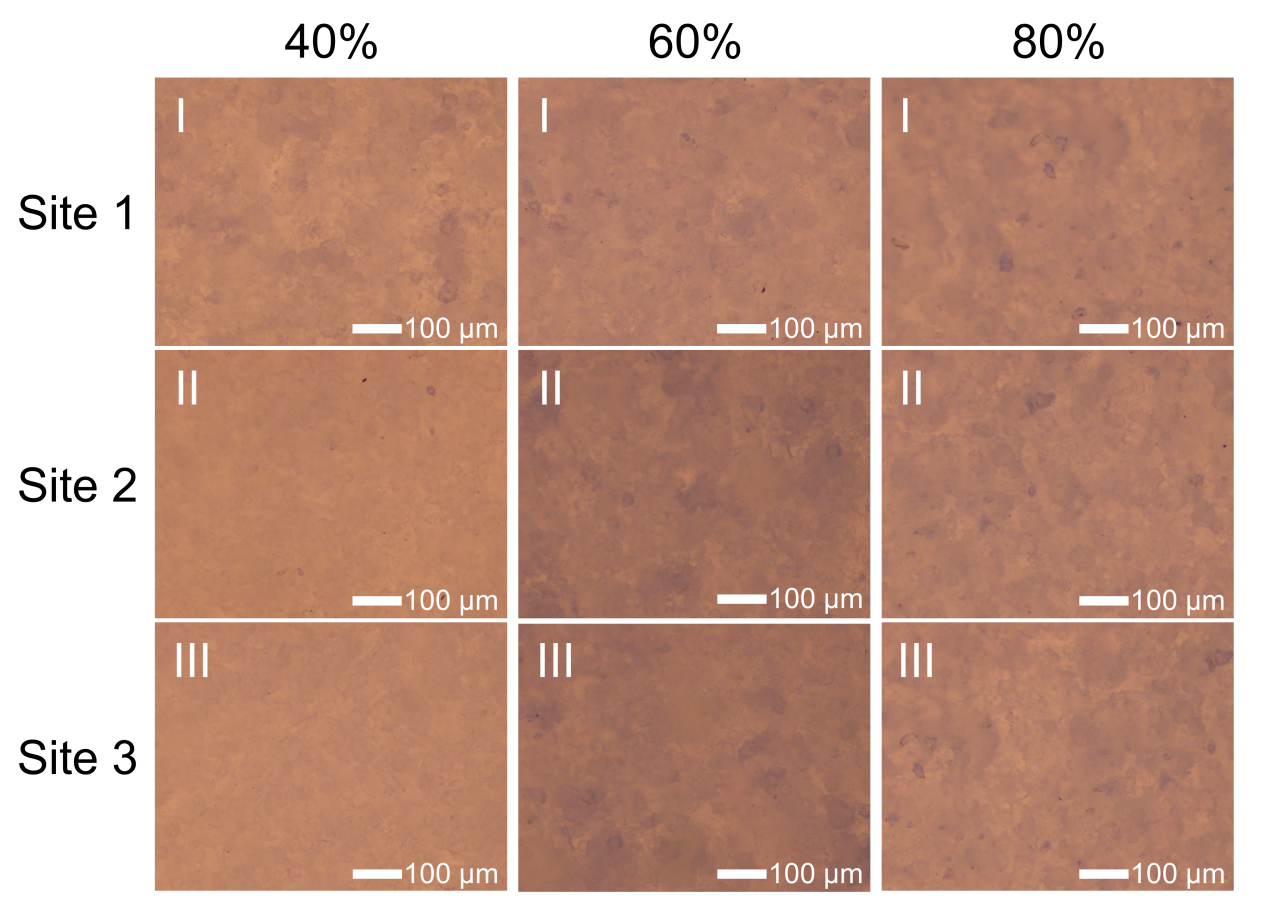


**Fig. S31** The color optical micrographs at the surface sites of the SBO:Pr/PDMS elastomer film under varied stretching strains after 10,000 continuous stretching cycles.


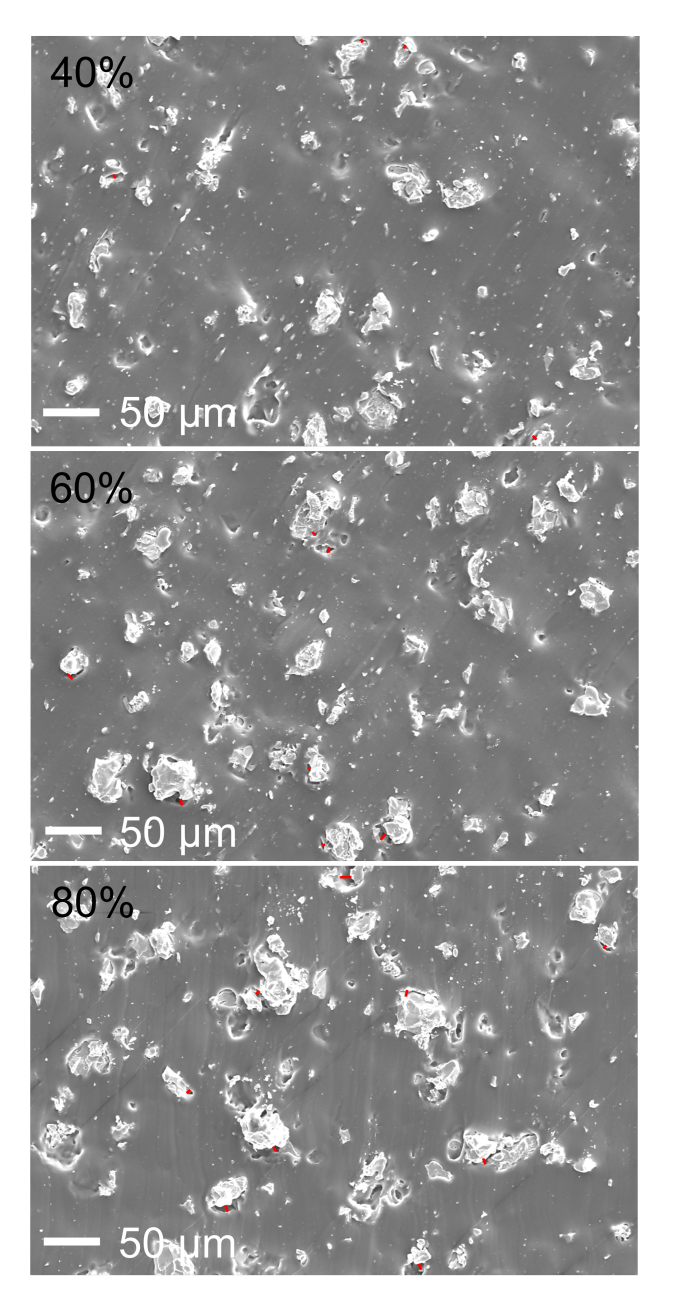


**Fig. S32** Cross-sectional SEM images of the film under different stretching strains.


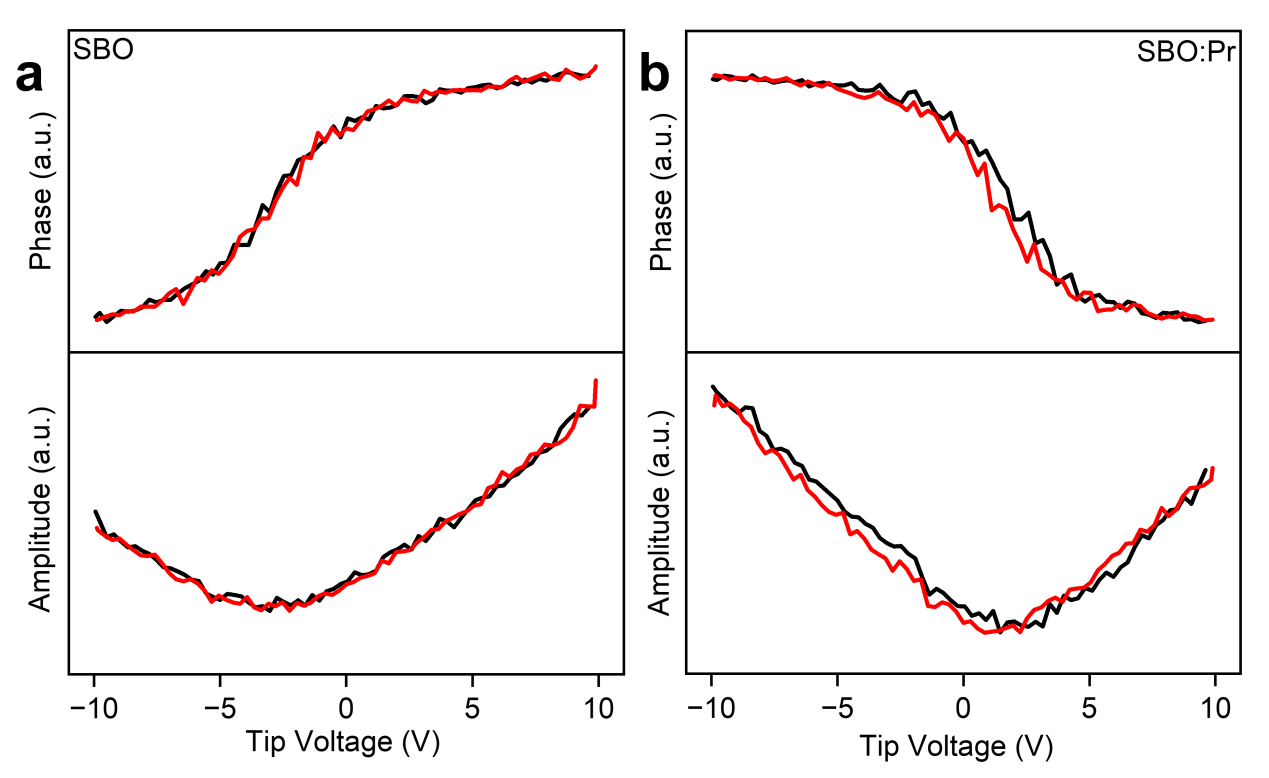


**Fig. S33** Phase and amplitude of piezoresponse force microscopies of **(a)** the SBO host and **(b)** the SBO:Pr phosphor.


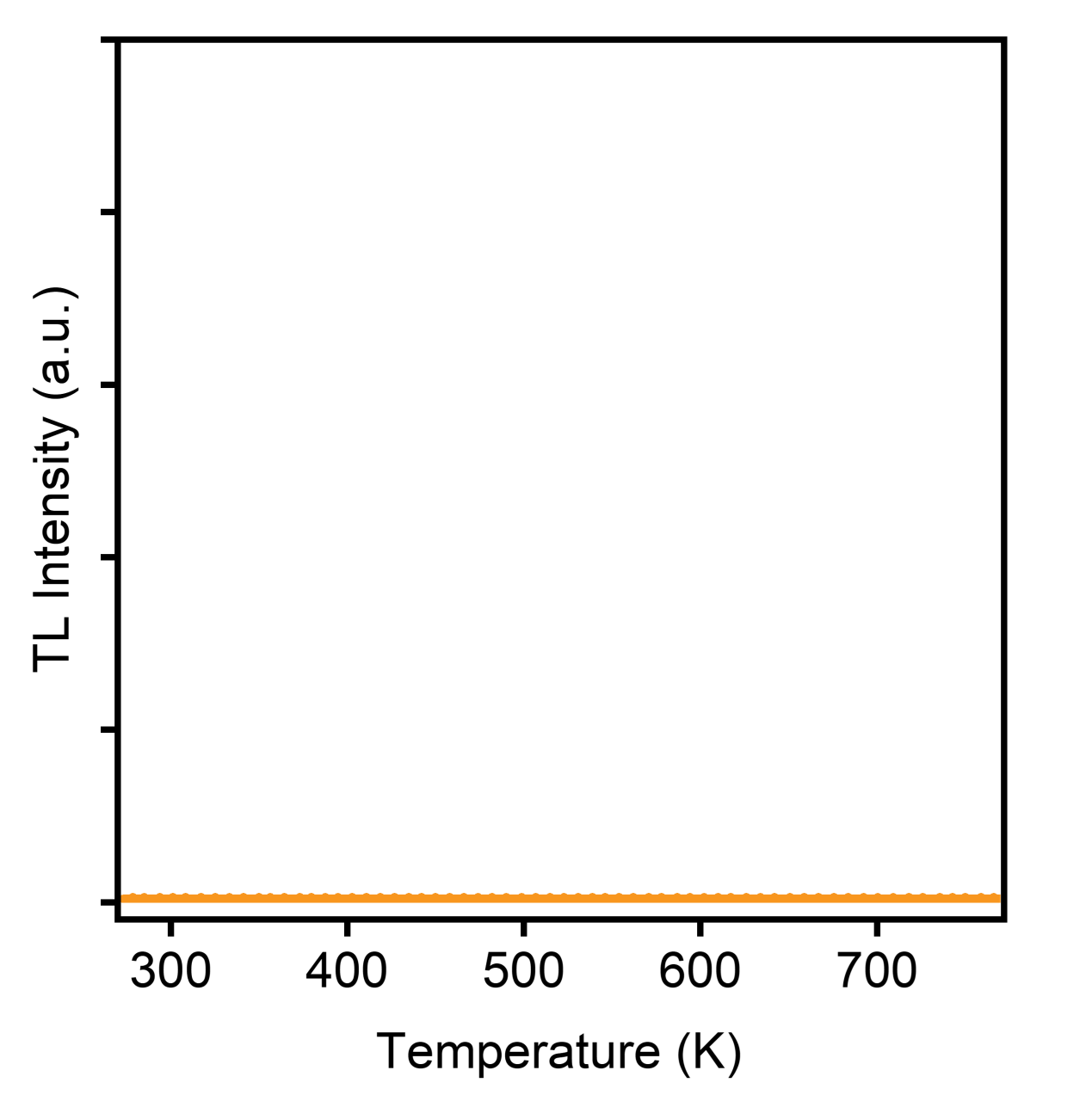


**Fig. S34** The TL curves of the SBO:Pr phosphor after natural placing for 30 days under the indoor lighting condition at room temperature.


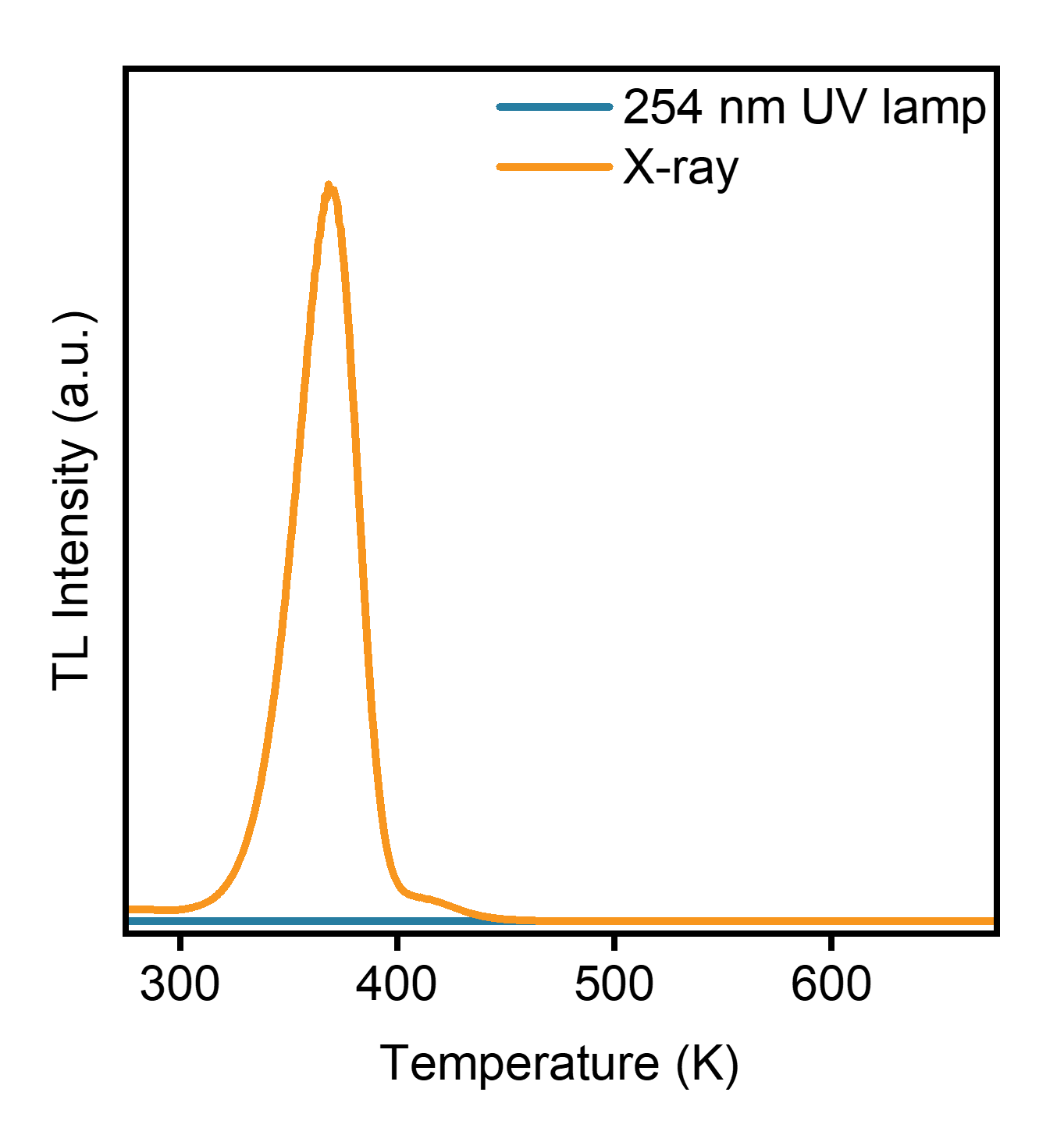


**Fig. S35** The TL curves of the SBO:Pr phosphor after X-ray or UV (254 nm UV lamp) irradiation.


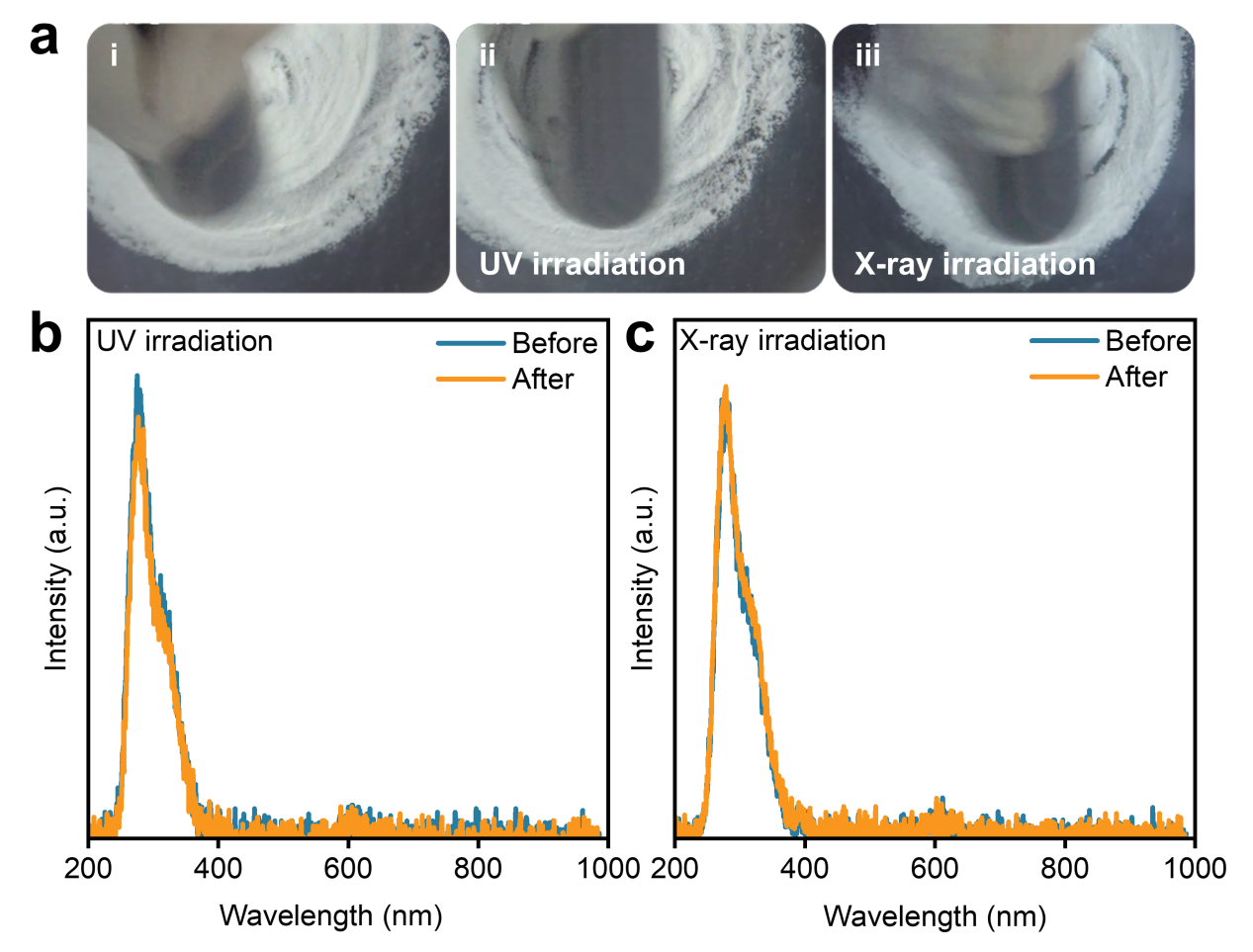


**Fig. S36** The ML behavior of the Sr_3_(BO_3_)_2_:Pr^3+^/PDMS ML elastomer and pure Sr_3_(BO_3_)_2_:Pr^3+^ phosphor before and after UV or X-ray irradiation.


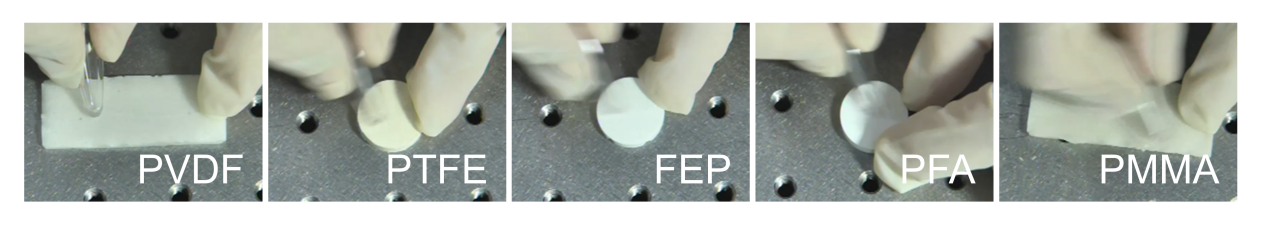


**Fig. S37** UVC ML images of the SBO:Pr phosphor embedded in the extended polymer matrices (PVDF, PTFE, FEP, PFA, and PMMA)


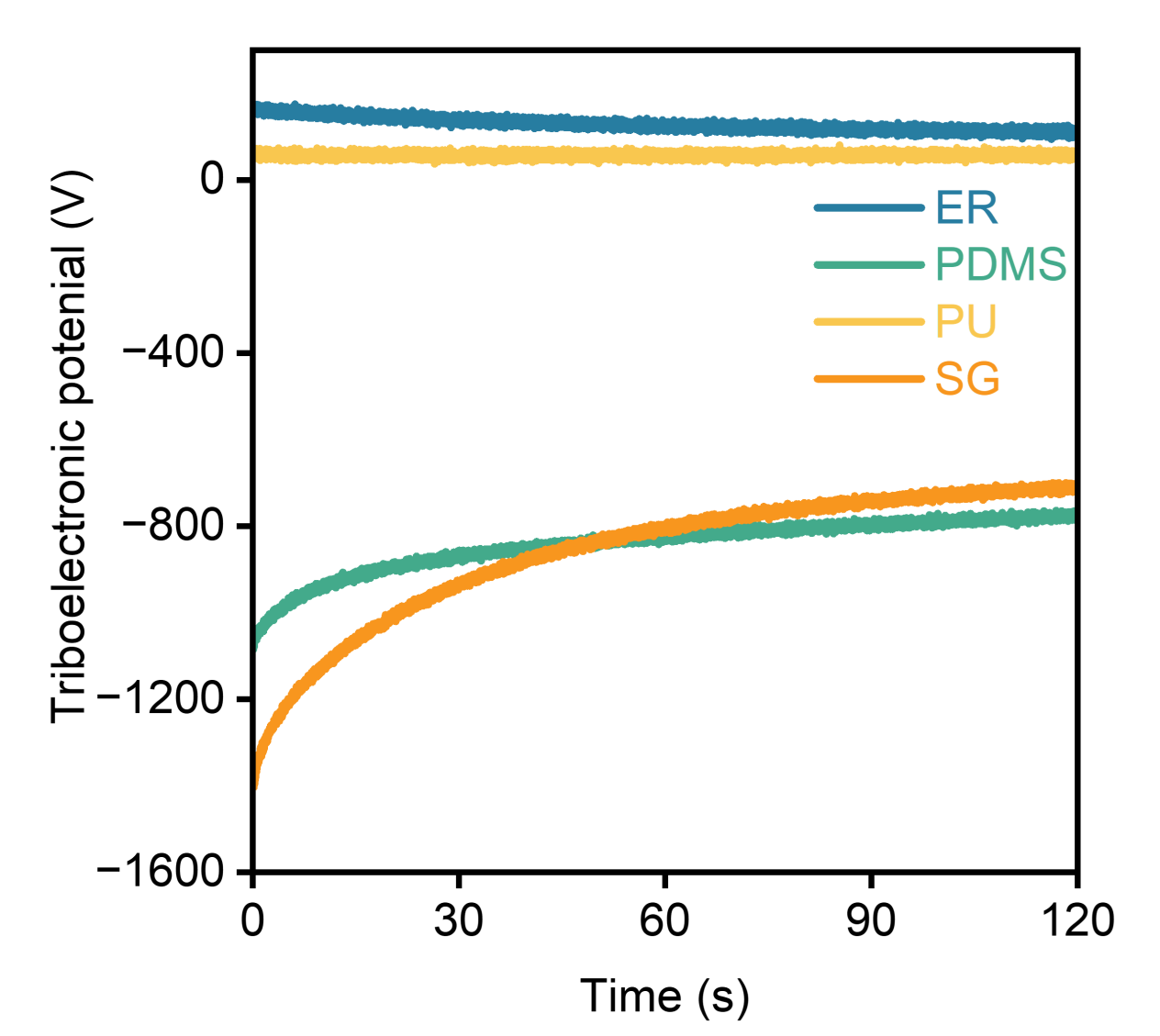


**Fig. S38** Attenuation curves of the triboelectric potential of various matrices after rubbing against SBO:Pr under 120 rpm for 1 min.


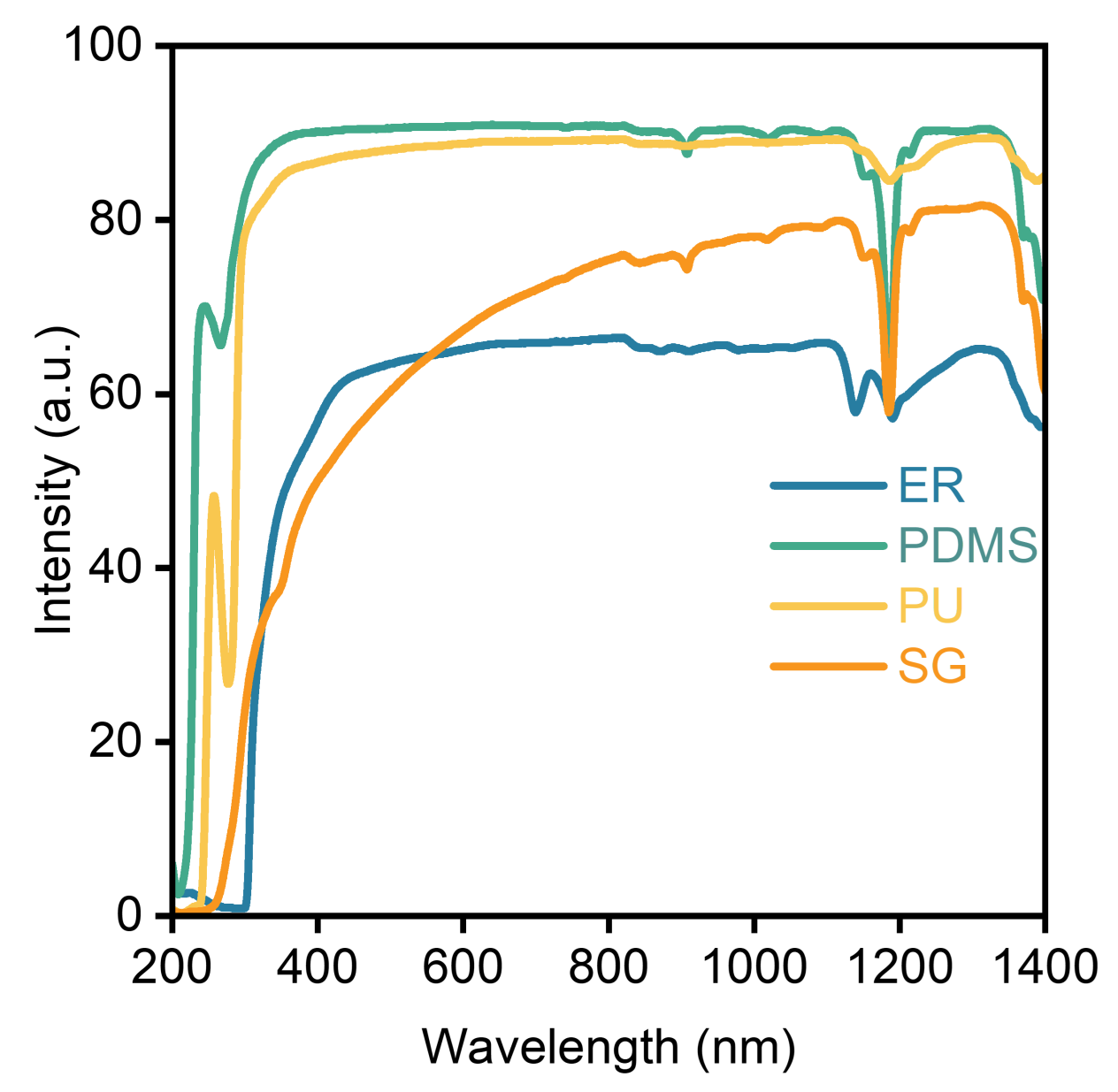


**Fig. S39** Transmittance of different matrices (ER, PDMS, PU and SG).


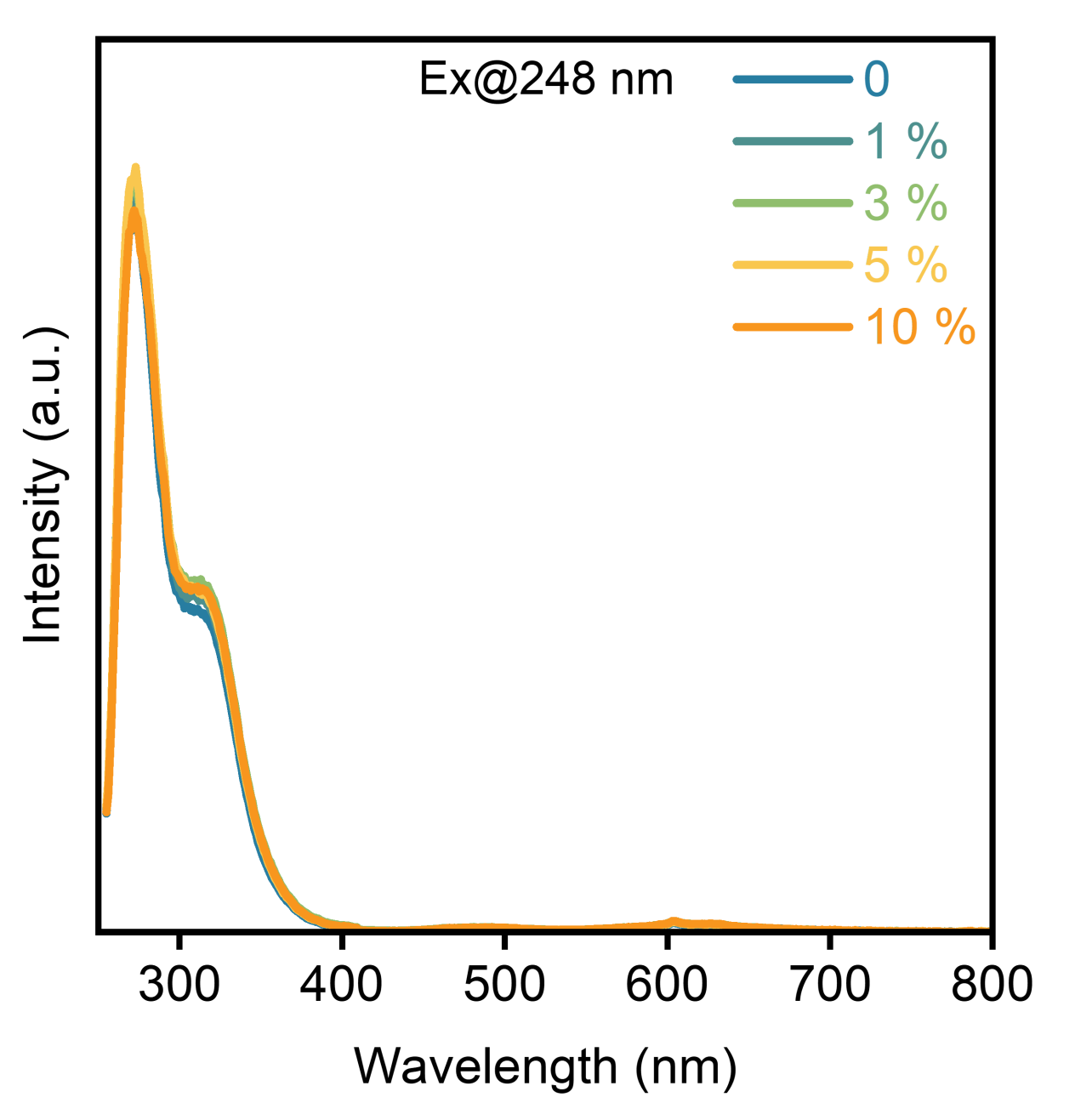


**Fig. S40** The PL emission spectra of the SBO:Pr/PDMS elastomer film with different release agent mass (the mass ratio between the release agent and PDMS).


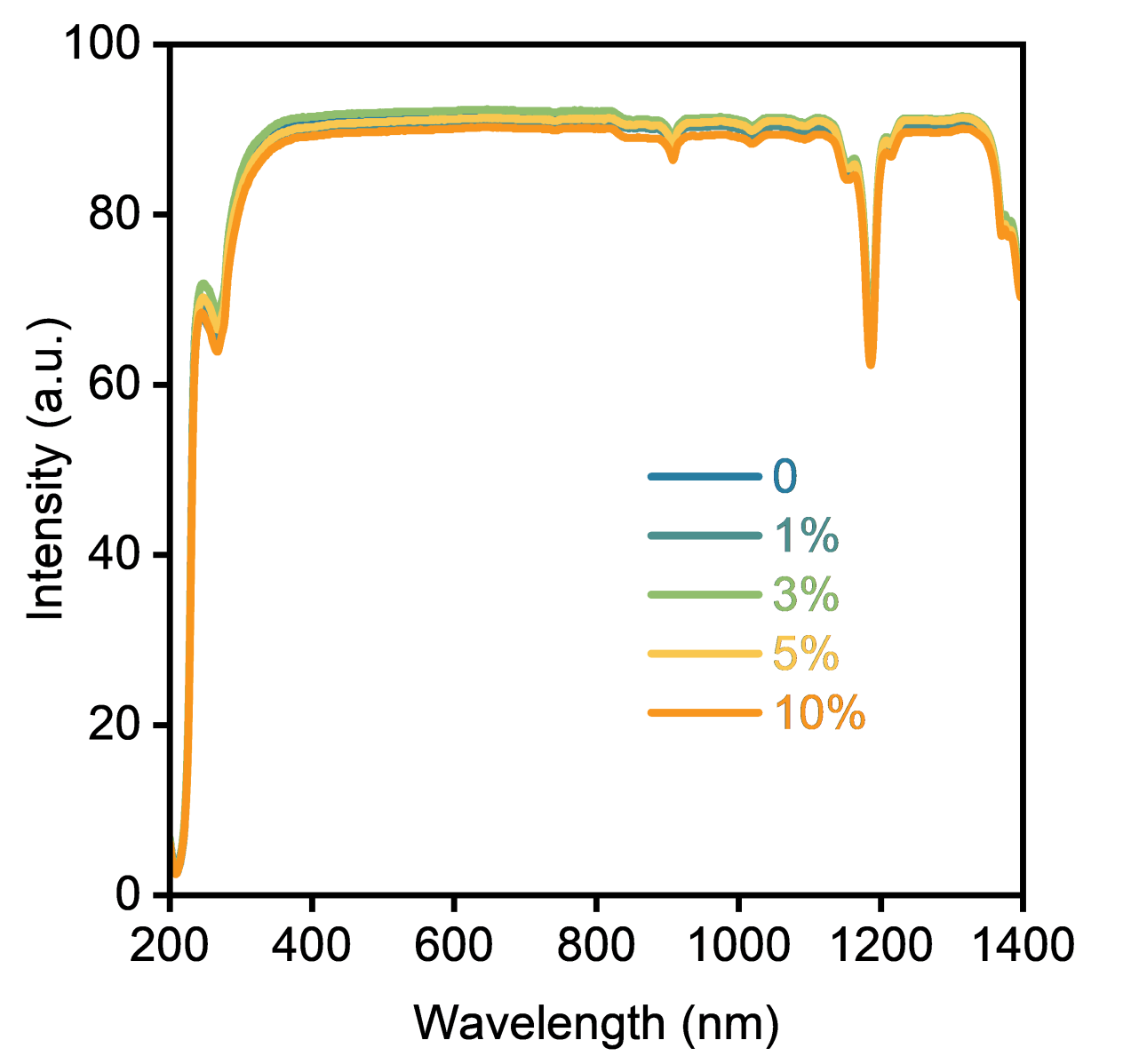


**Fig. S41** Transmittance of the PDMS matrix with different release agent mass (the mass ratio between the release agent and PDMS).


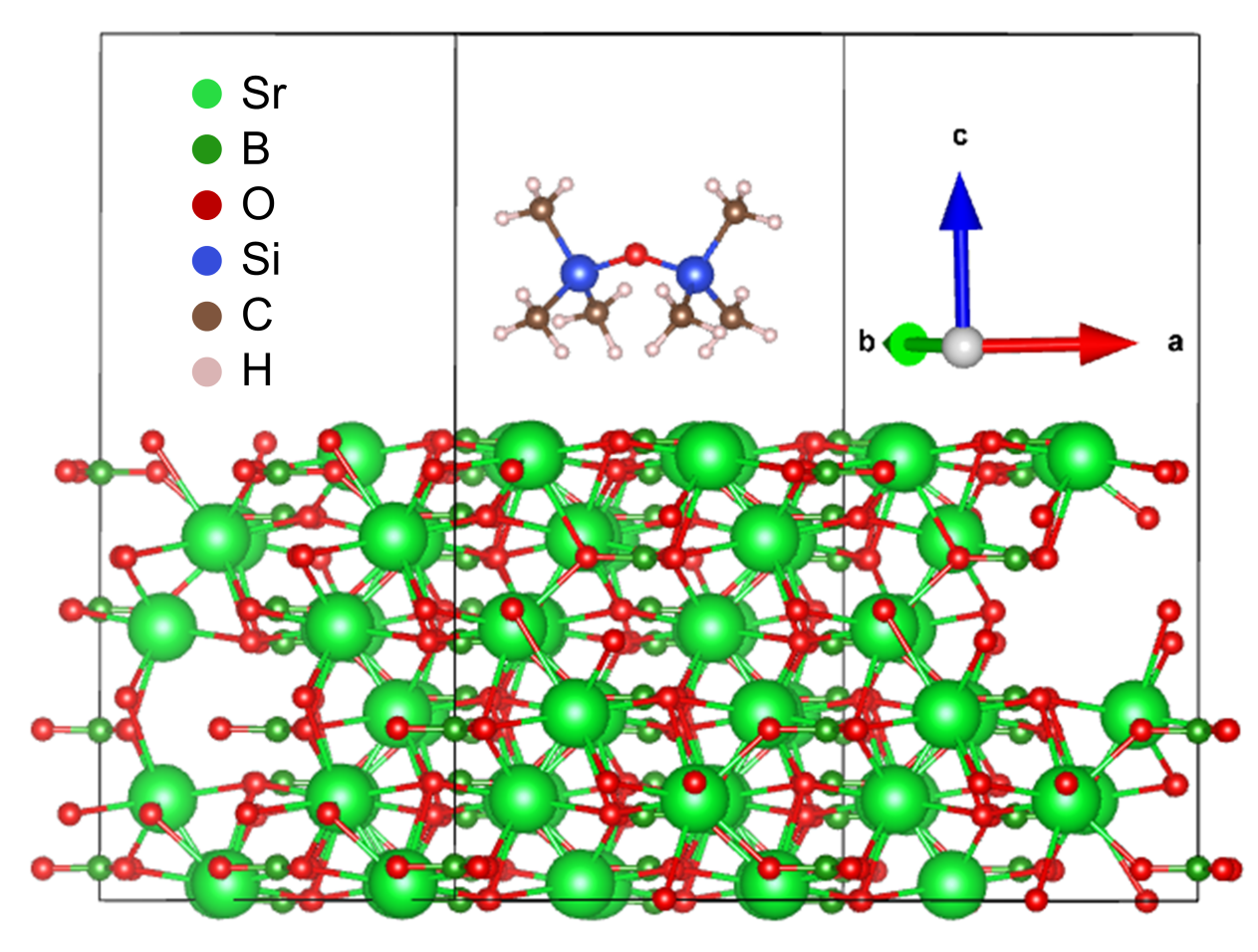


**Fig. S42** The contact models of SBO-amorphous PDMS monomer on SBO (001) surface.


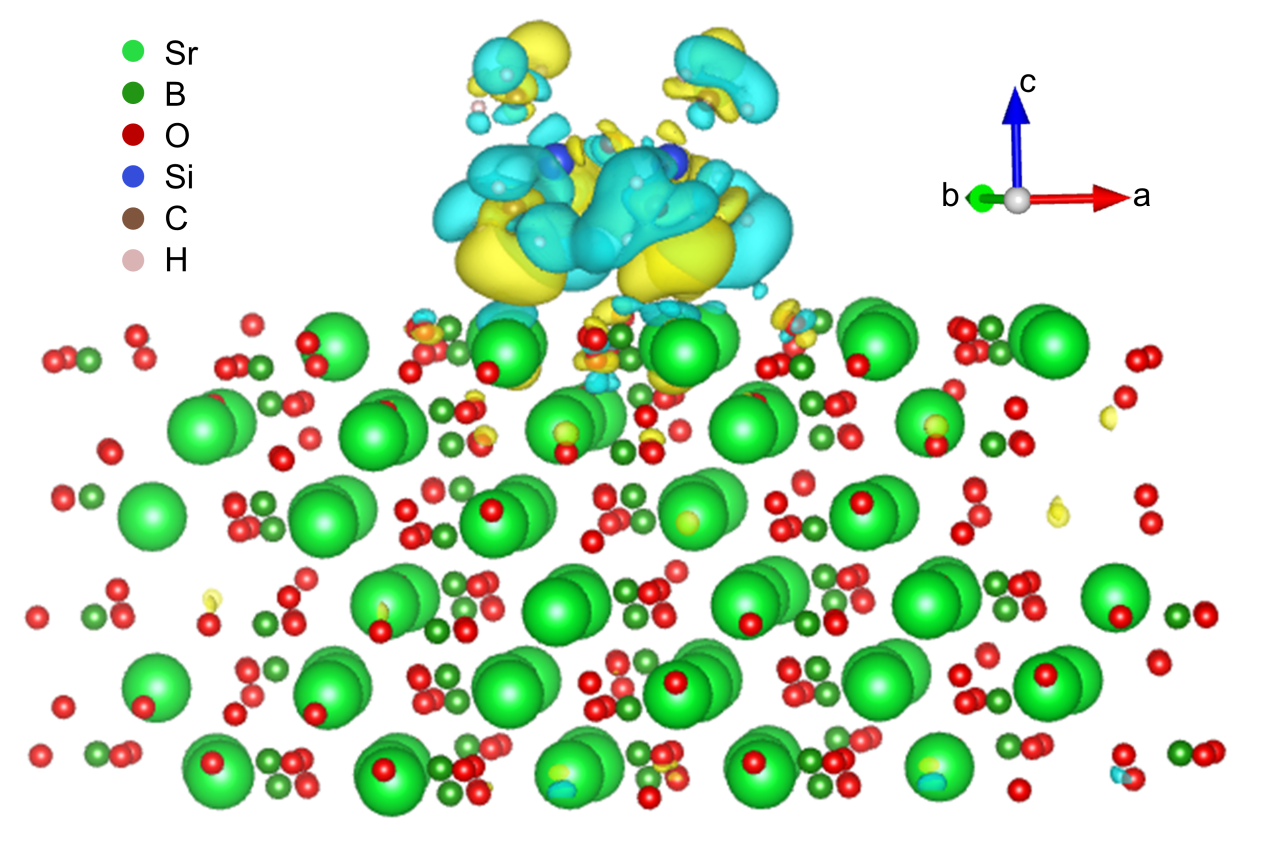


**Fig. S43** Regions of electron-gain (yellow) and electronloss (light-blue) at SBO-PDMS monomer interface.


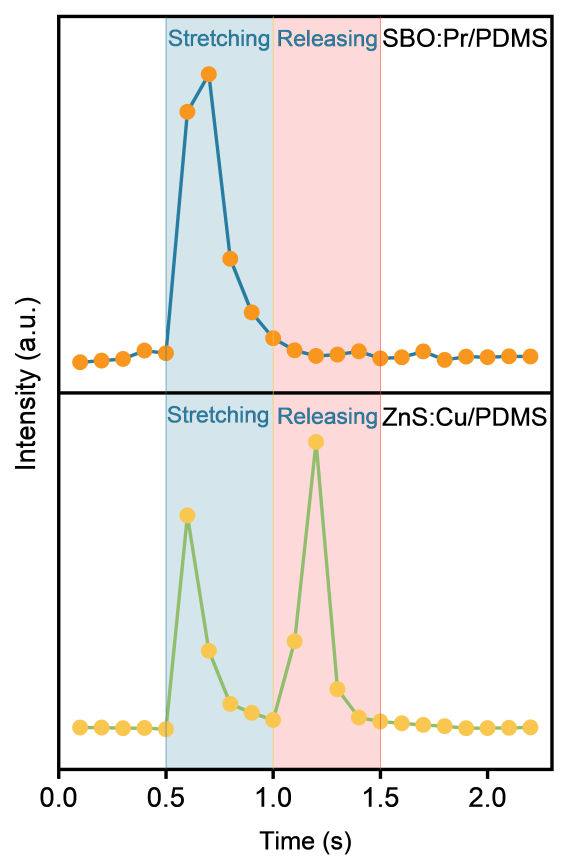


**Fig. S44** ML behaviors of the ZnS:Cu/PDMS and SBO:Pr/PDMS composite elastomers during a single stretching cycle.

During a single cycle, both SBO:Pr/PDMS and ZnS:Cu/PDMS exhibit rapid ML, reaching the maximum at the start of stretching due to interfacial triboelectrification. Thus, the ML is generated when the separation of the phosphor particles and polymer chains occurs, which lies in the beginning of stretching. However, ZnS:Cu/PDMS also shows ML during releasing in one cycle, giving a double-peak profile. This is because ZnS:Cu is a special alternating-current electroluminescence (EL) material, and the releasing process can also excite it to emit light. Therefore, the above single cycle ML behaviors confirm the different mechanisms between the SBO:Pr/PDMS and ZnS:Cu/PDMS elastomers and further strengthen the discussion of the ML mechanism.


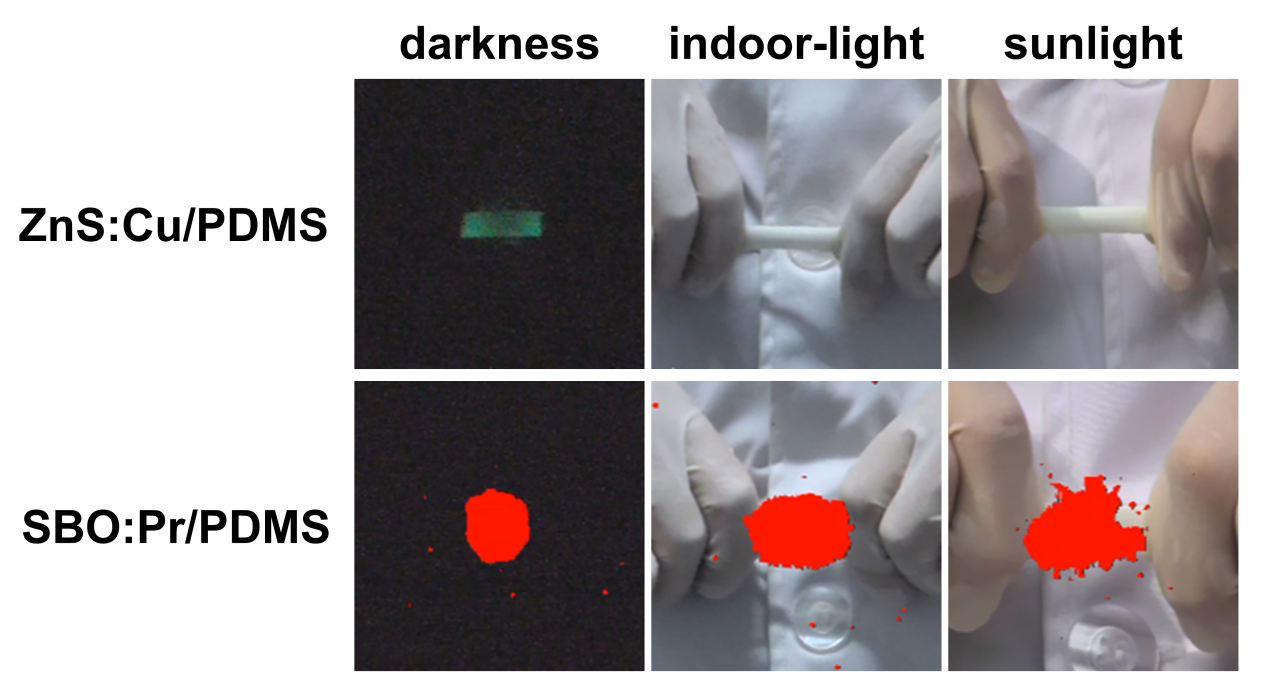


**Fig. S45** UVC ML comparison images of SBO:Pr/PDMS and ZnS:Cu/PDMS elastomer films under different environmental conditions over stretching stimulus.


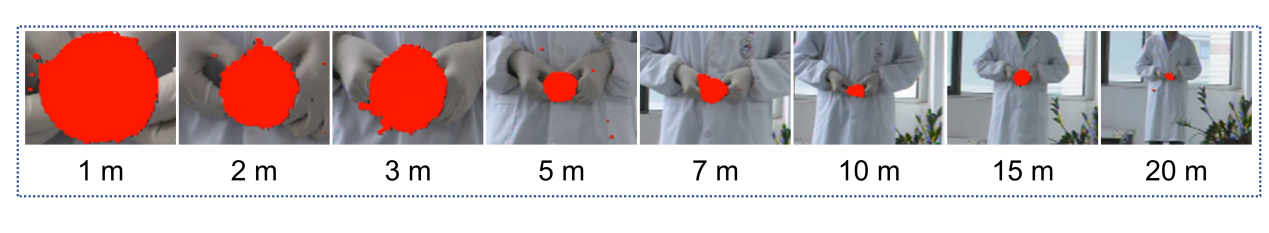


**Fig. S46** UVC ML images at different distances.


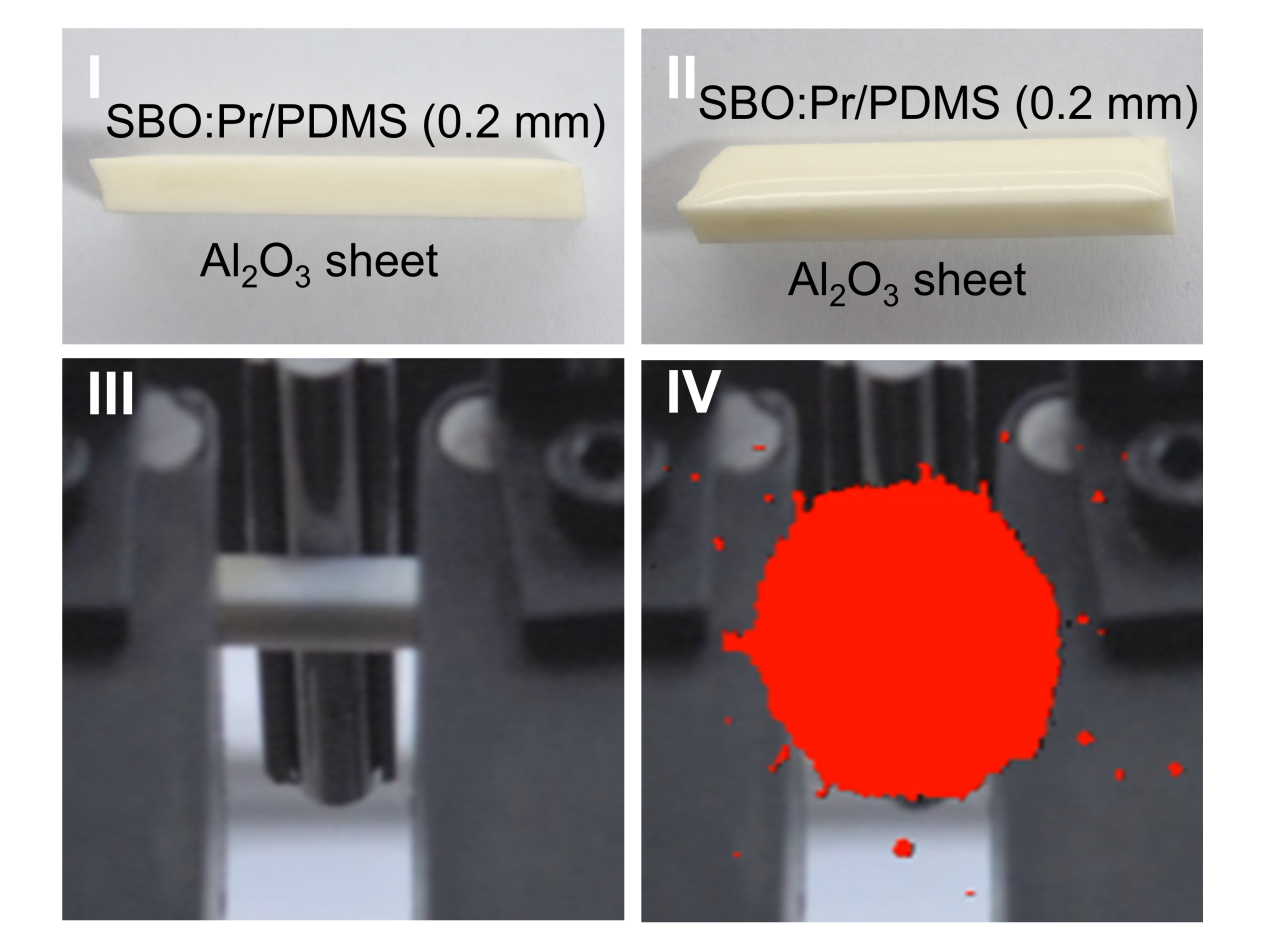


**Fig. S47** Demonstration of structural health monitoring. **I** and **II** show images of SBO:Pr/PDMS coated Al_2_O_3_ sheet. **III** and **IV** show the UVC ML of SBO:Pr/PDMS coated Al_2_O_3_ sheet under stress.


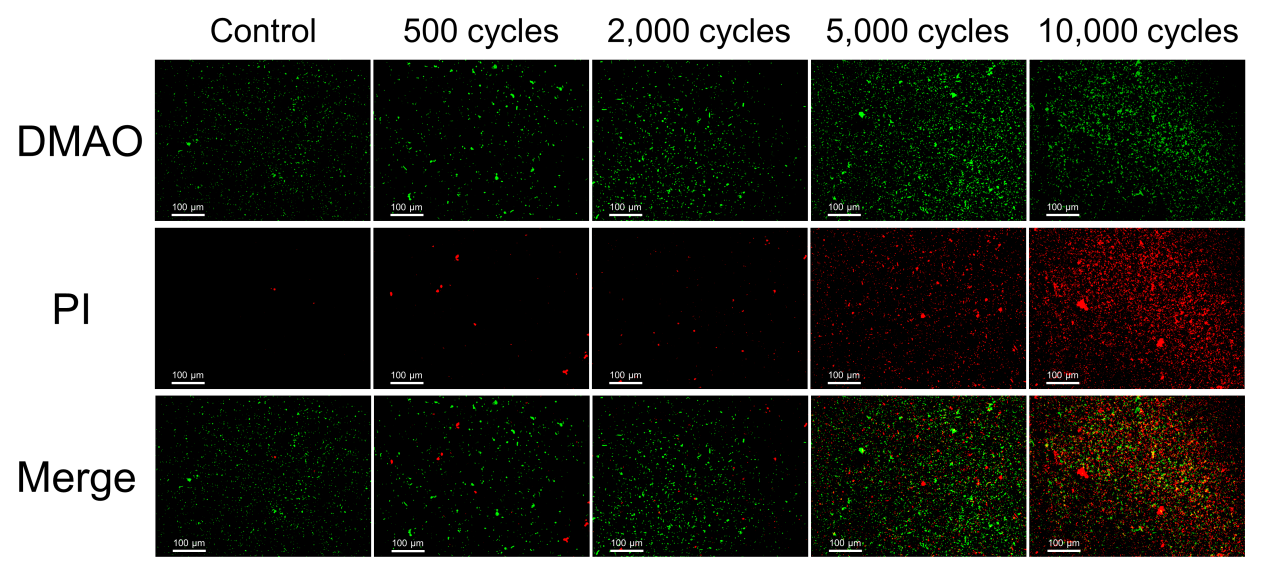


**Fig. S48** Inactivation of Methicillin-resistant *Staphylococcus aureus* using UVC ML.


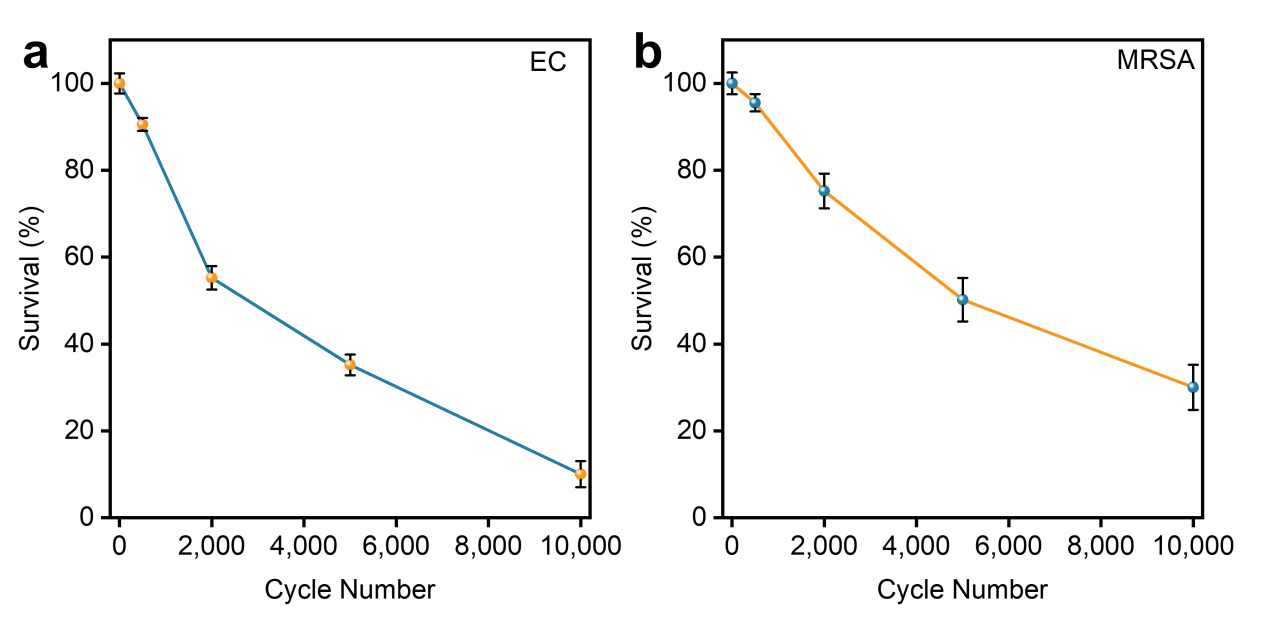


**Fig. S49** The dependence of EC and MRSA survival ratios on the stretching cycle.


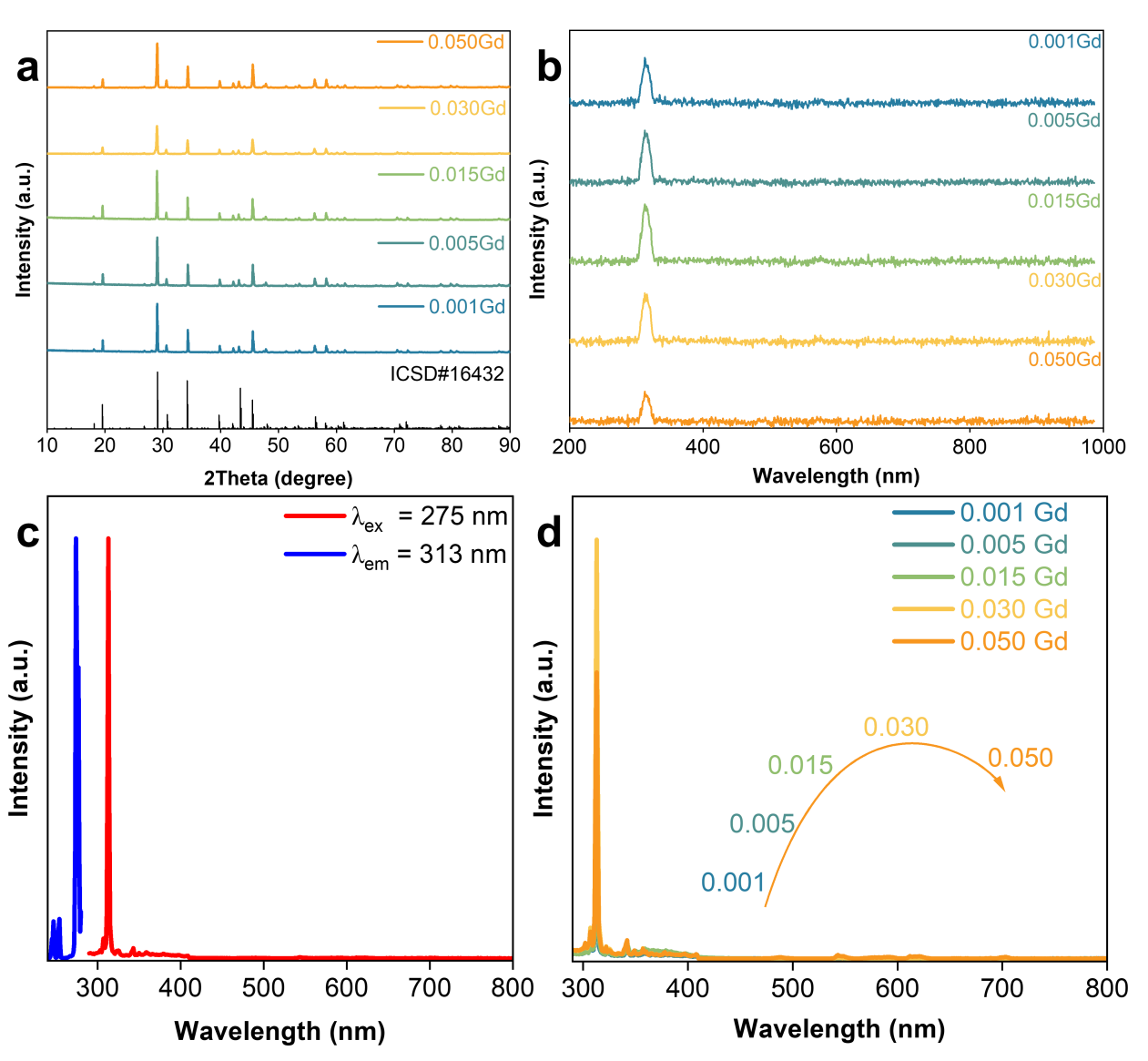


**Fig. S50** **(a)** XRD patterns of the SBO:*x*Gd phosphors. **(b)** ML emission spectra of the SBO:*x*Gd/PDMS elastomer films. **(c)** Normalized PL excitation and emission spectra of the SBO:Gd phosphor. **(d)** PL emission spectra of the SBO:*x*Gd phosphors.


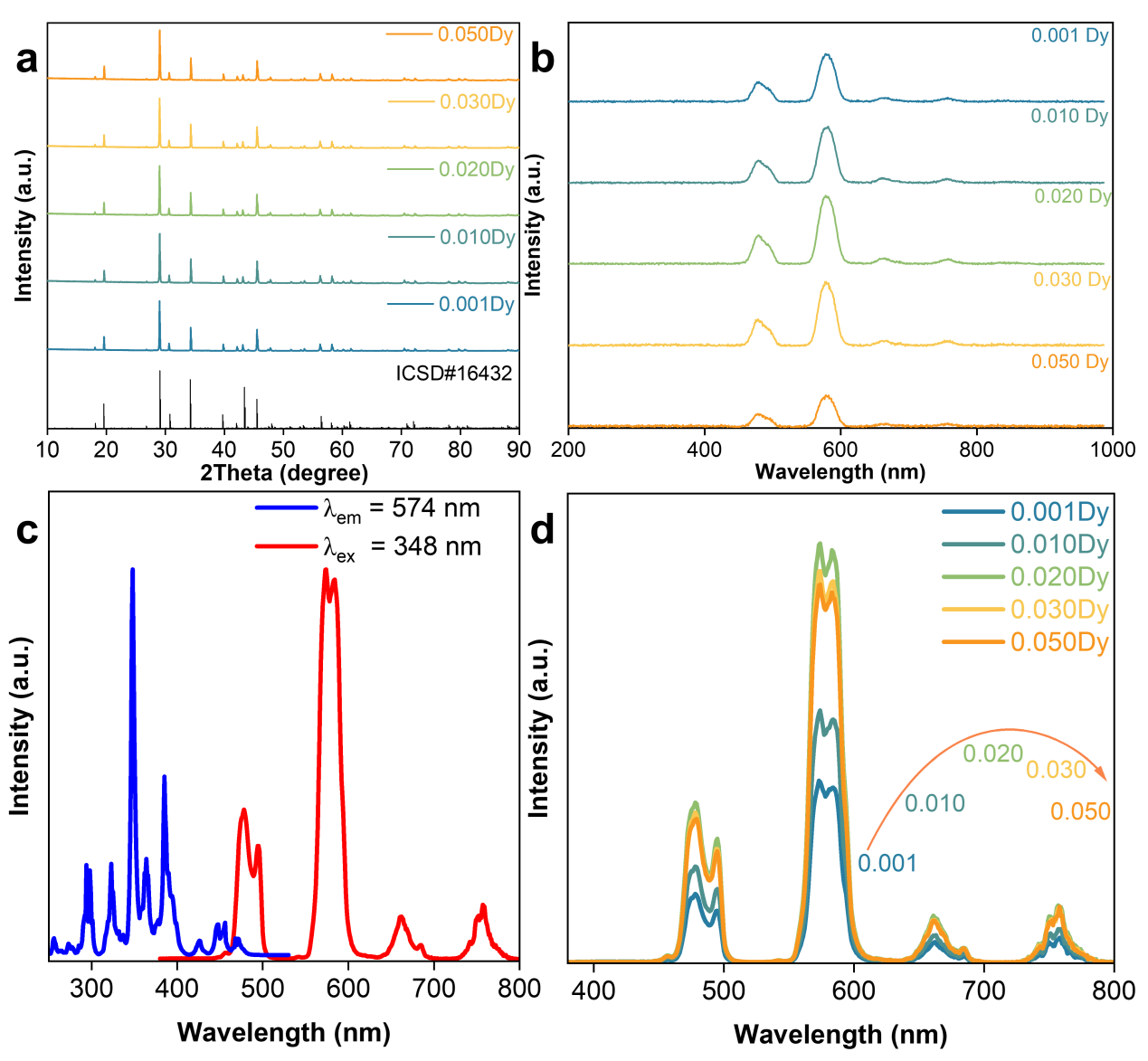


**Fig. S51** **(a)** XRD patterns of the SBO:*x*Dy phosphors. **(b)** ML emission spectra of the SBO:*x*Dy/PDMS elastomer films. **(c)** Normalized PL excitation and emission spectra of the SBO:Dy phosphor. **(d)** PL emission spectra of the SBO:*x*Dy phosphors.


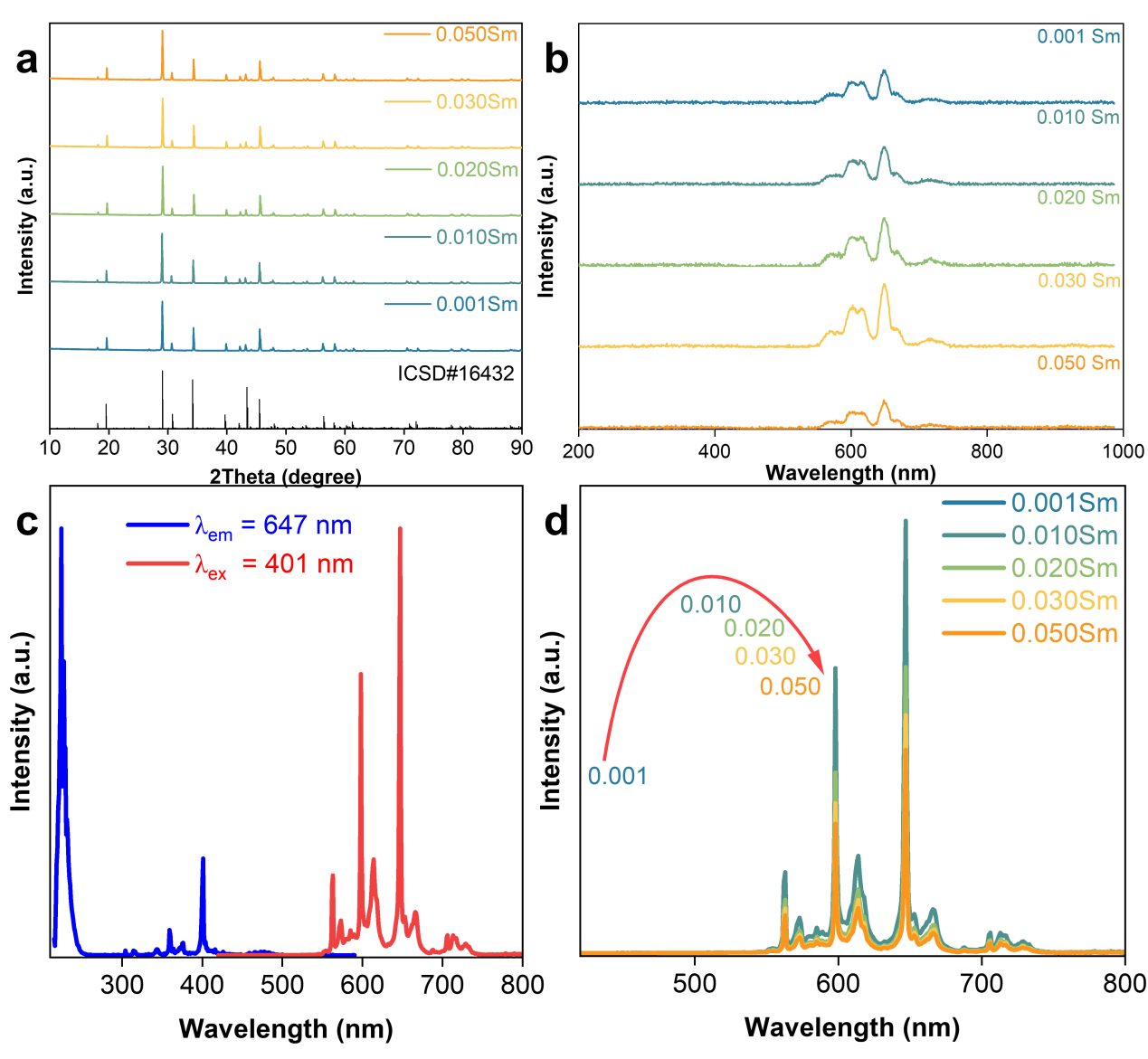


**Fig. S52** **(a)** XRD patterns of the SBO:*x*Sm phosphors. **(b)** ML emission spectra of the SBO:*x*Sm/PDMS elastomer films. **(c)** Normalized PL excitation and emission spectra of the SBO:Sm phosphor. **(d)** PL emission spectra of the SBO:*x*Sm phosphors.


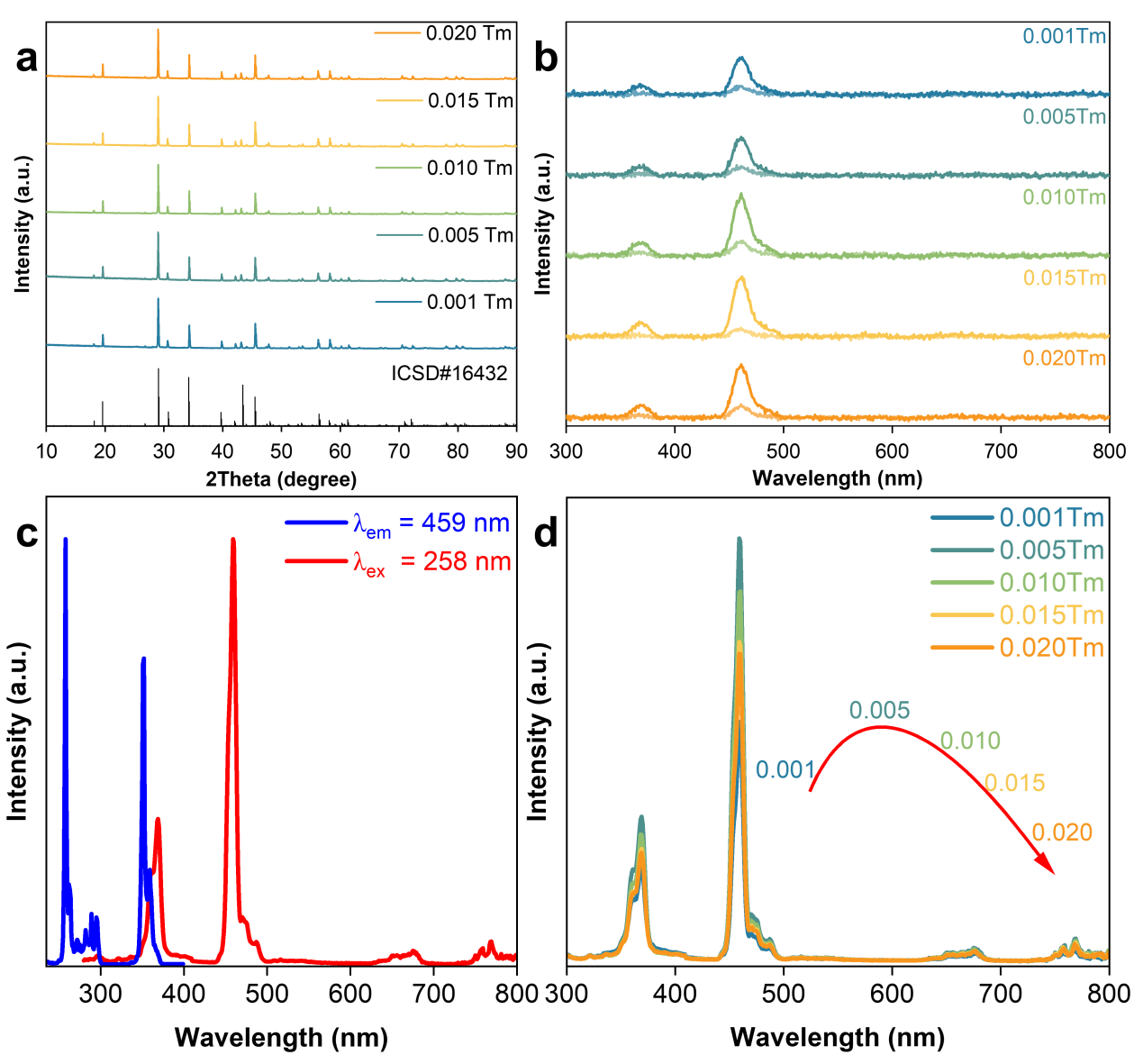


**Fig. S53** **(a)** XRD patterns of the SBO:*x*Tm phosphors. **(b)** ML emission spectra of the SBO:*x*Tm/PDMS elastomer films. **(c)** Normalized PL excitation and emission spectra of the SBO:Tm phosphor. **(d)** PL emission spectra of SBO:*x*Tm phosphors.


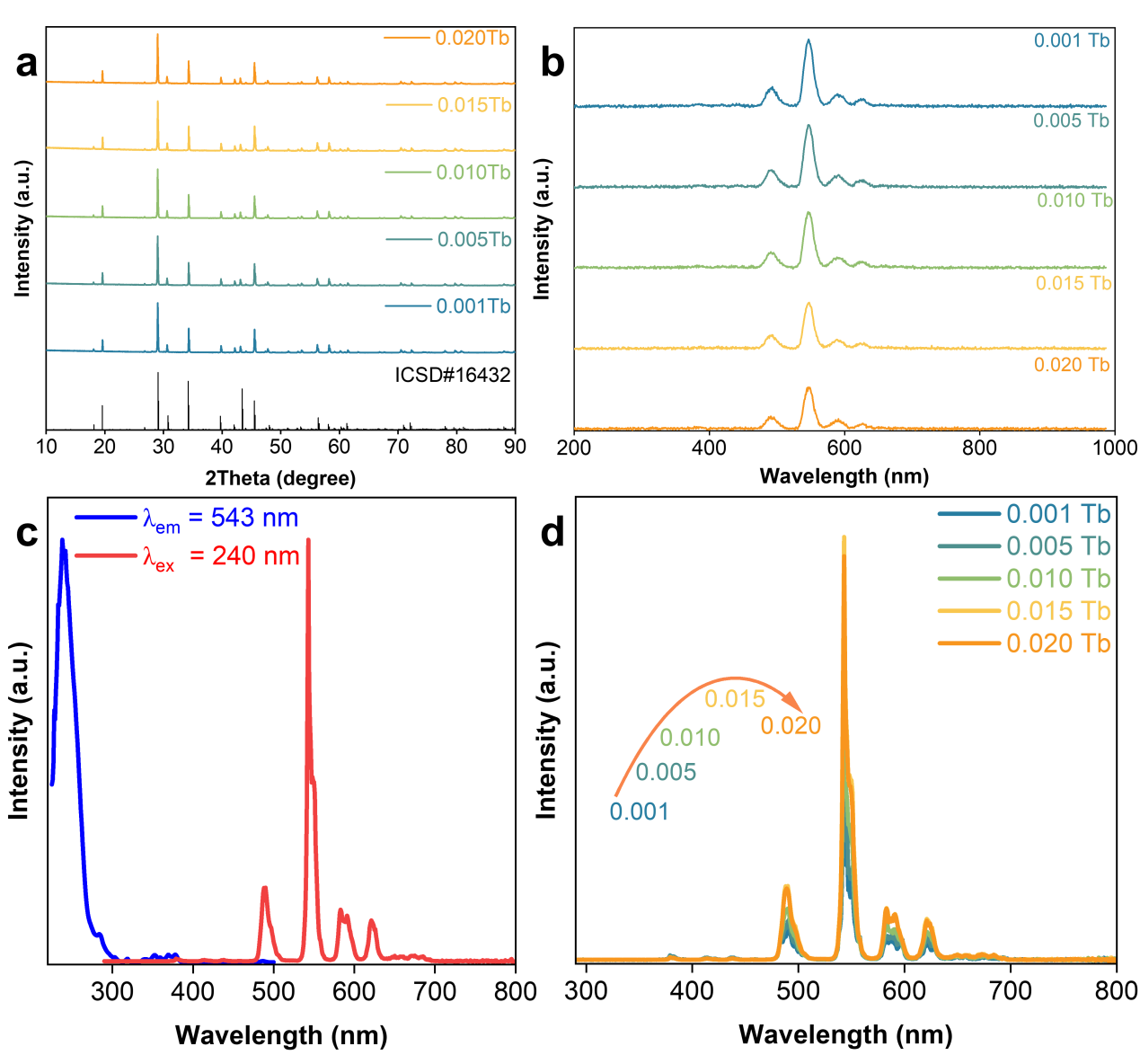


**Fig. S54** **(a)** XRD patterns of the SBO:*x*Tb phosphors. **(b)** ML emission spectra of the SBO:*x*Tb/PDMS elastomer films. **(c)** Normalized PL excitation and emission spectra of the SBO:Tb phosphor. **(d)** PL emission spectra of the SBO:*x*Tb phosphors.


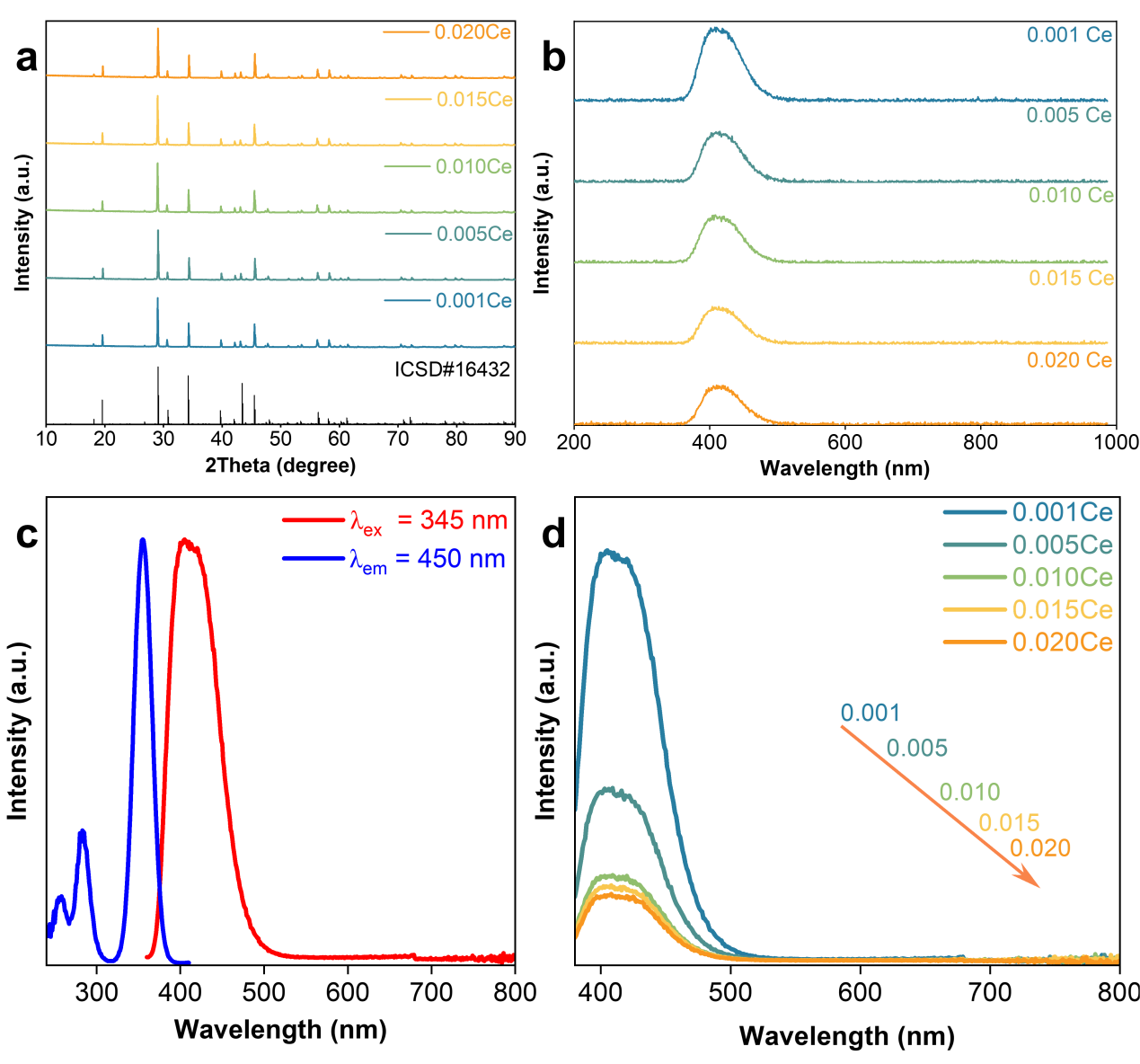


**Fig. S55** **(a)** XRD patterns of the SBO:*x*Ce phosphors. **(b)** ML emission spectra of the SBO:*x*Ce/PDMS elastomer films. **(c)** Normalized PL excitation and emission spectra of the SBO:Ce phosphor. **(d)** PL emission spectra of the SBO:*x*Ce phosphors.


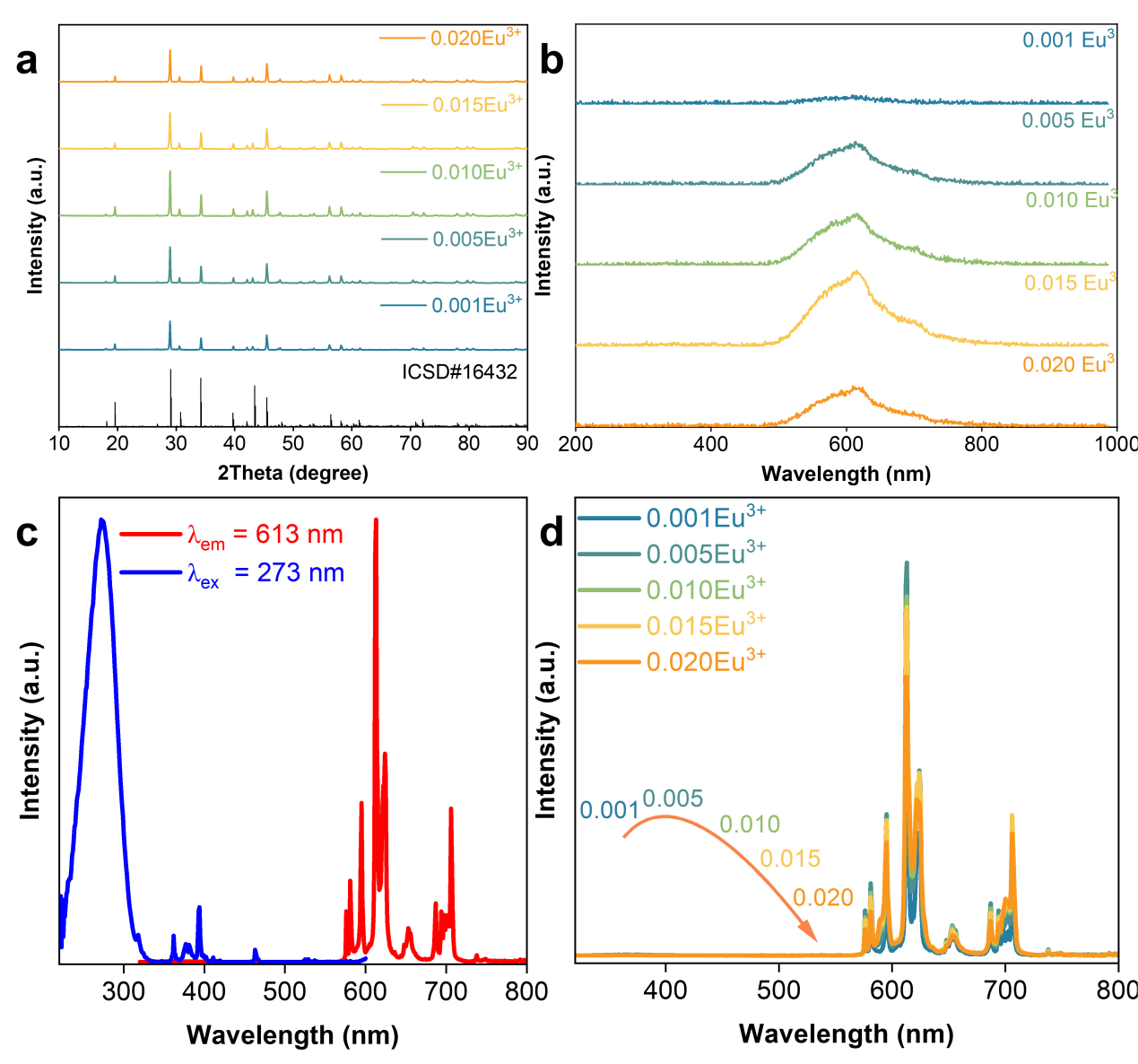


**Fig. S56** **(a)** XRD patterns of the SBO:*x*Eu^3+^ phosphors. **(b)** ML emission spectra of the SBO:*x*Eu^3+^/PDMS elastomer films. **(c)** Normalized PL excitation and emission spectra of the SBO:Eu^3+^ phosphor. **(d)** PL emission spectra of the SBO:*x*Eu^3+^ phosphors.


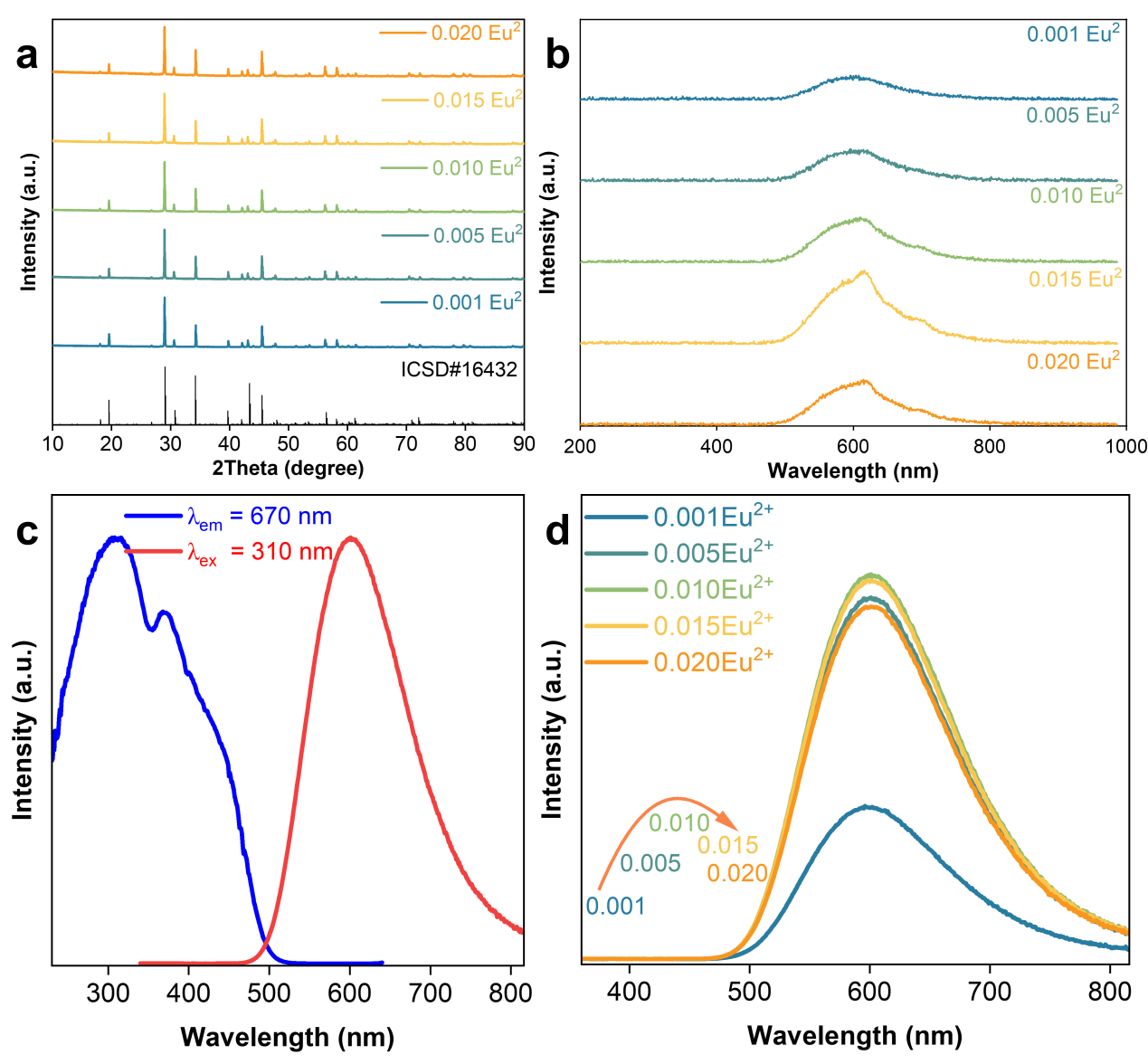


**Fig. S57** **(a)** XRD patterns of the SBO:*x*Eu^2+^ phosphors. **(b)** ML emission spectra of the SBO:*x*Eu^2+^/PDMS elastomer films. **(c)** Normalized PL excitation and emission spectra of the SBO:Eu^2+^ phosphor. **(d)** PL emission spectra of the SBO:*x*Eu^2+^ phosphors.


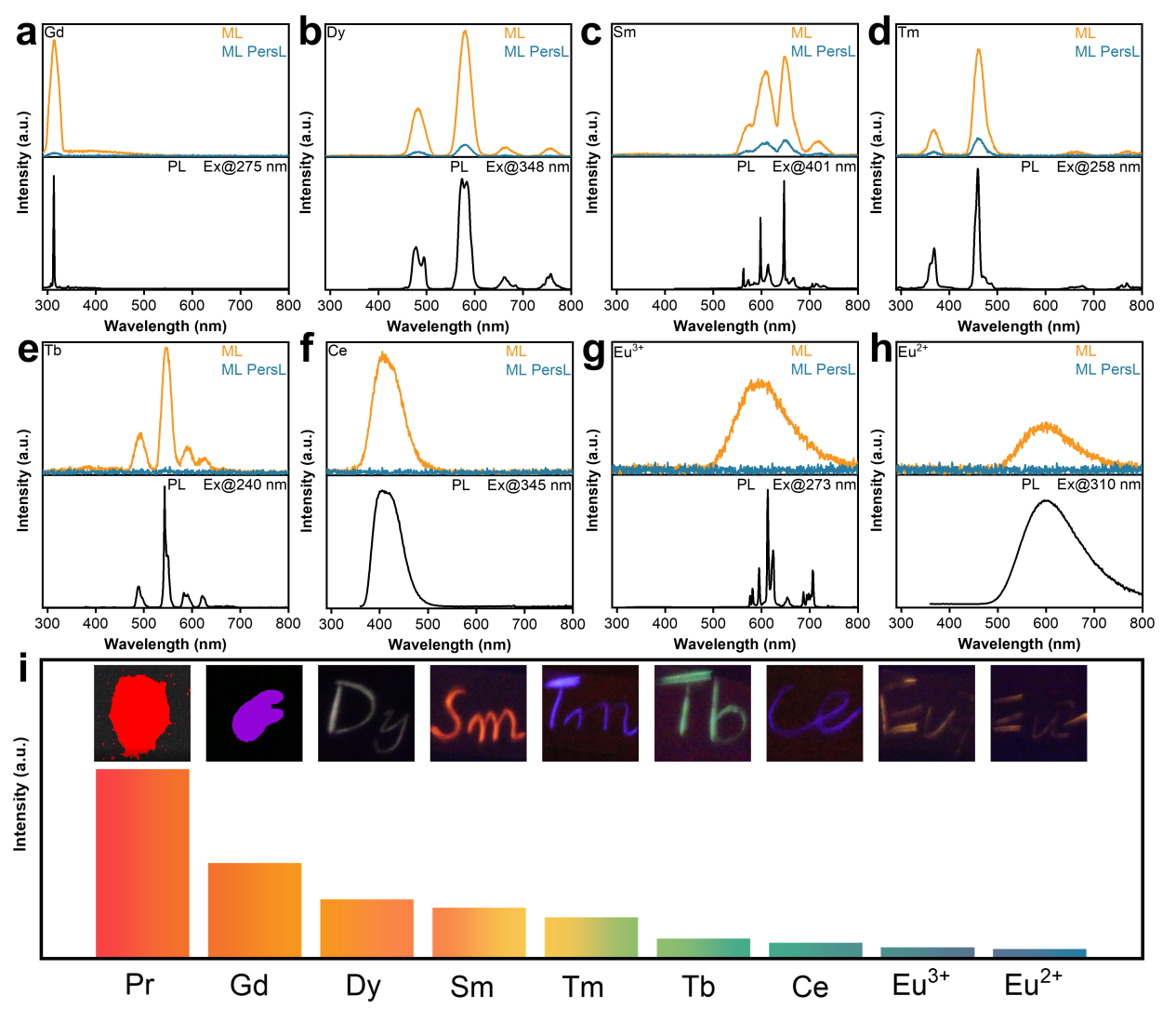


**Fig. S58 ML spectral tunability of the SBO:Ln/PDMS elastomer film.** The ML and PL emission spectra of the SBO:Ln/PDMS elastomer film, **(a)** Gd^3+^, **(b)** Dy^3+^, **(c)** Sm^3+^, **(d)** Tm^3+^, **(e)** Ce^3+^, **(f)** Tb^3+^, **(g)** Eu^3+^ and **(h)** Eu^2+^. **(i)** Comparison of the ML intensities of the SBO:Ln/PDMS elastomer film. Insets show photos of the corresponding elastomer films under external rubbing.


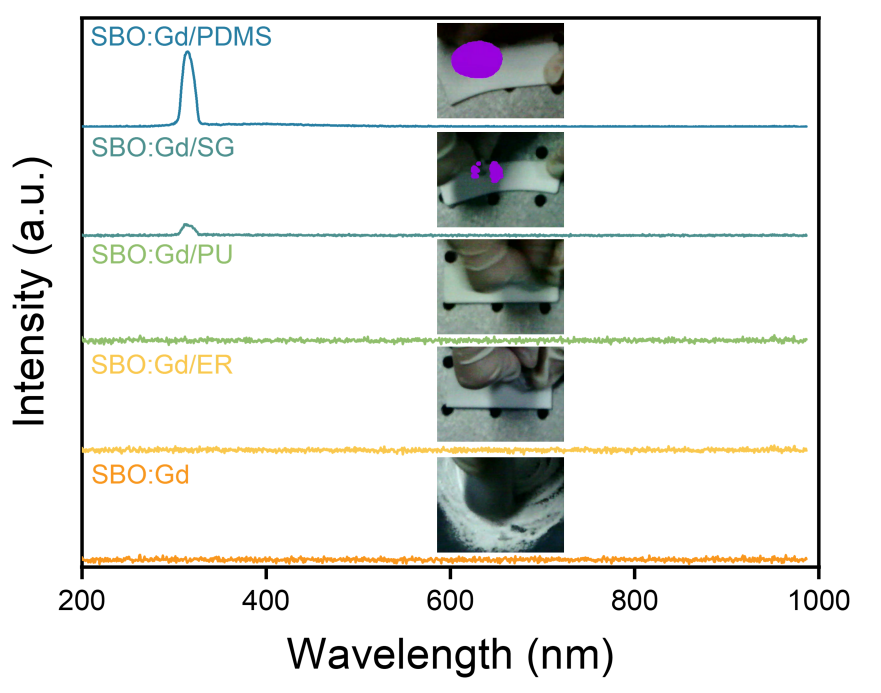


**Fig. S59** ML emission spectra of SBO:Gd phosphors embedded in different matrices (ER, PU, SG and PDMS) under rubbing stimuli. Insets show the corresponding ML images.


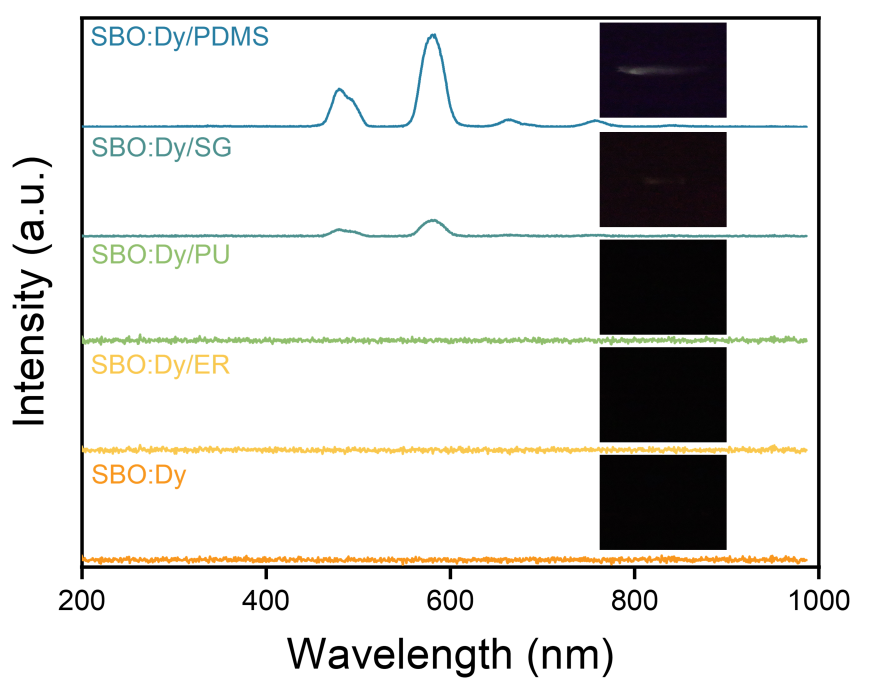


**Fig. S60** ML emission spectra of SBO:Dy phosphors embedded in different matrices (ER, PU, SG and PDMS) under rubbing stimuli. Insets show the corresponding ML images.


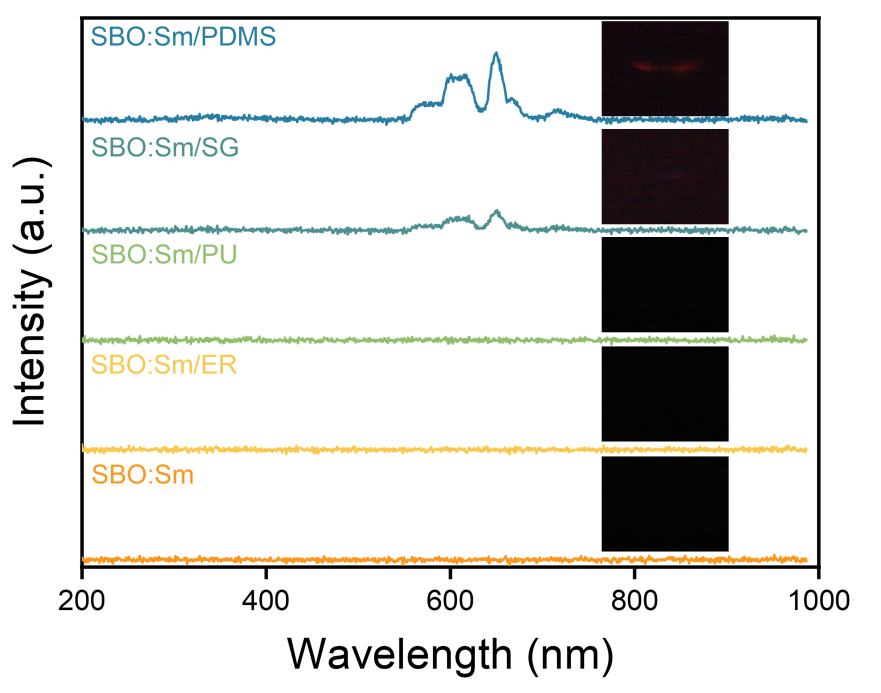


**Fig. S61** ML emission spectra of SBO:Sm phosphors embedded in different matrices (ER, PU, SG and PDMS) under rubbing stimuli. Insets show the corresponding ML images.


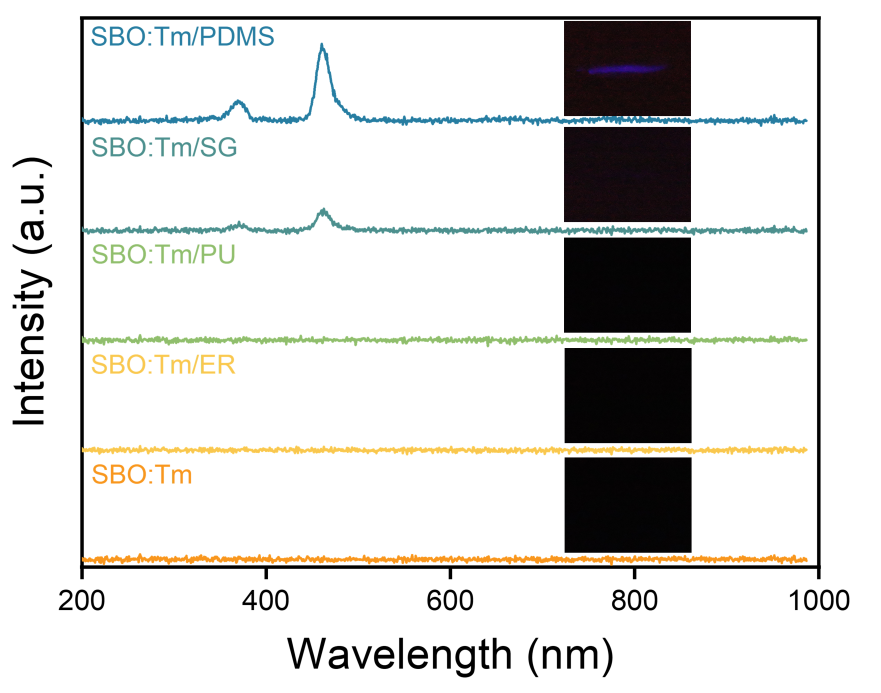


**Fig. S62** ML emission spectra of SBO:Tm phosphors embedded in different matrices (ER, PU, SG and PDMS) under rubbing stimuli. Insets show the corresponding ML images.


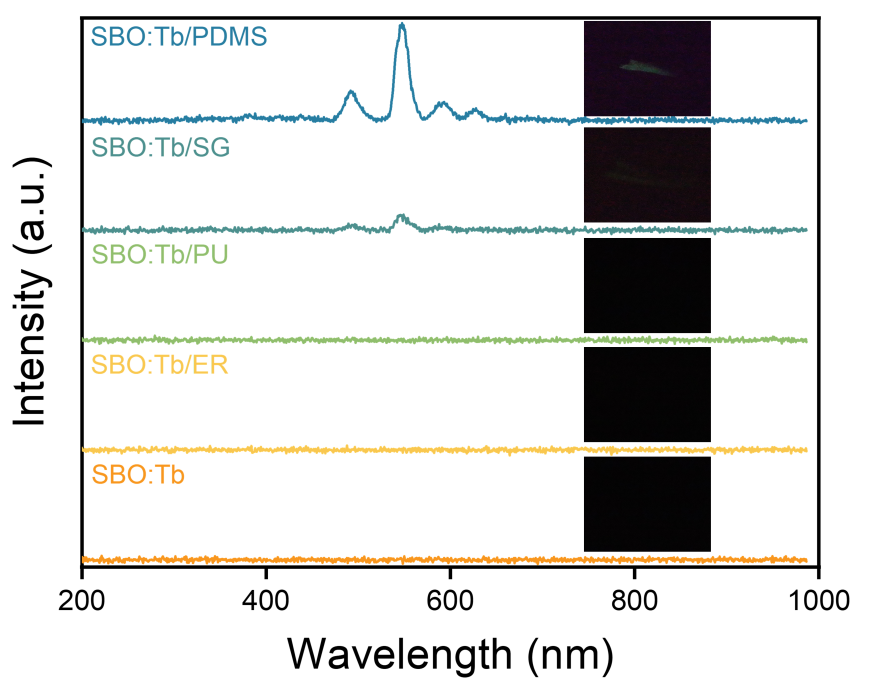


**Fig. S63** ML emission spectra of SBO:Tb phosphors embedded in different matrices (ER, PU, SG and PDMS) under rubbing stimuli. Insets show the corresponding ML images.


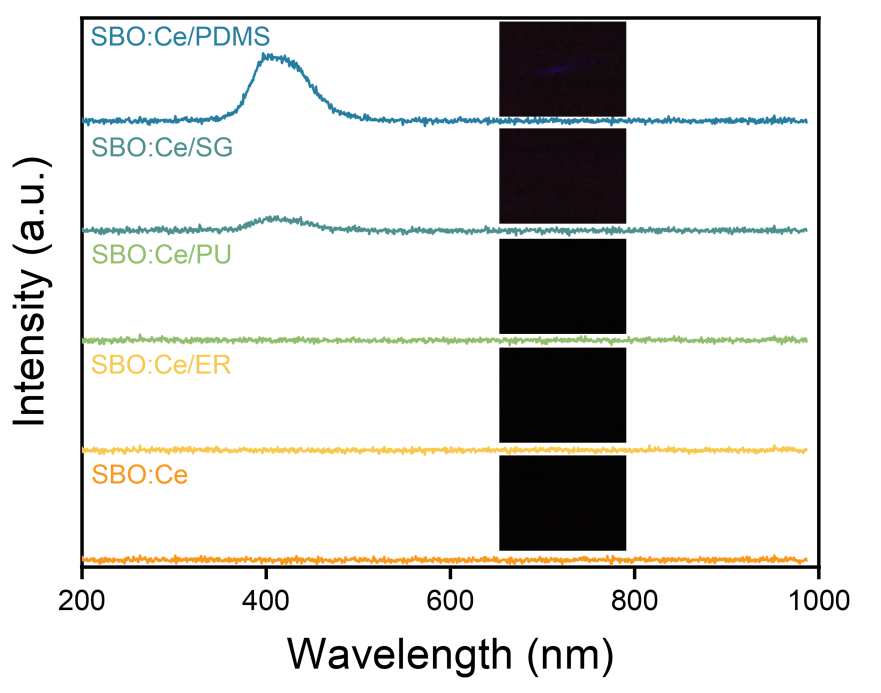


**Fig. S64** ML emission spectra of SBO:Ce phosphors embedded in different matrices (ER, PU, SG and PDMS) under rubbing stimuli. Insets show the corresponding ML images.


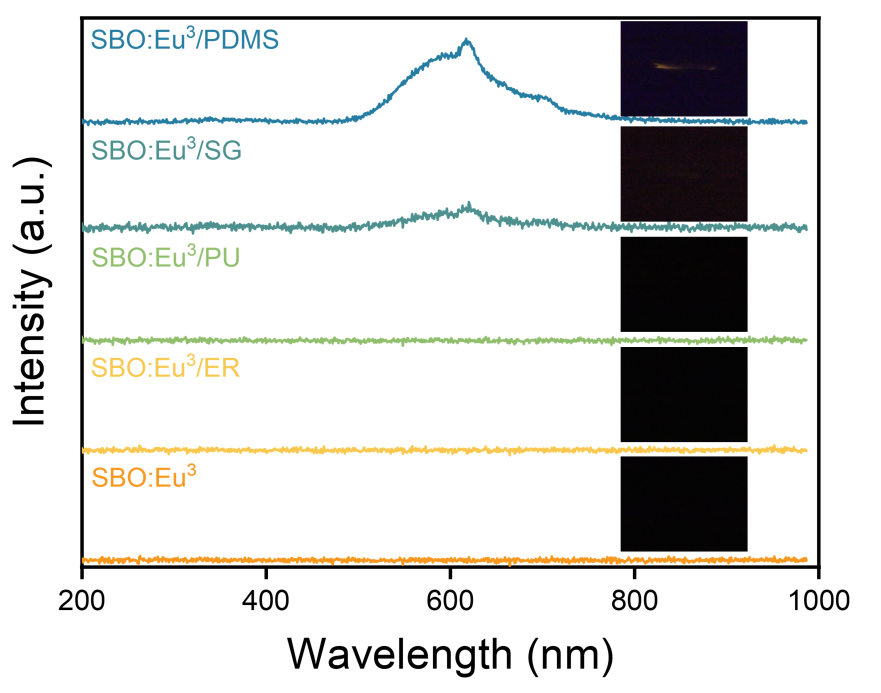


**Fig. S65** ML emission spectra of SBO:Eu^3^ phosphors embedded in different matrices (ER, PU, SG and PDMS) under rubbing stimuli. Insets show the corresponding ML images.


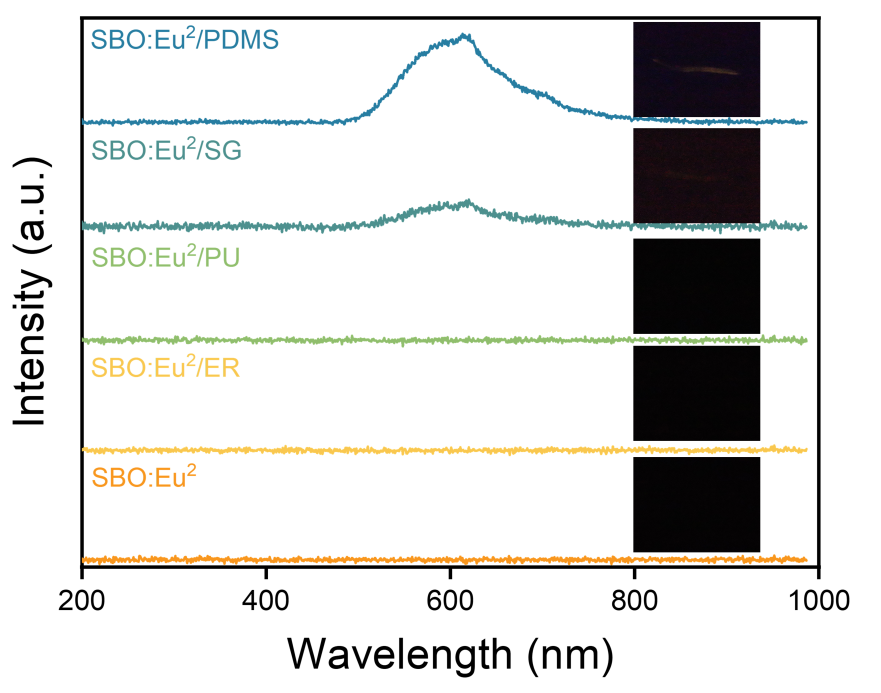


**Fig. S66** ML emission spectra of SBO:Eu^2^ phosphors embedded in different matrices (ER, PU, SG and PDMS) under rubbing stimuli. Insets show the corresponding ML images.


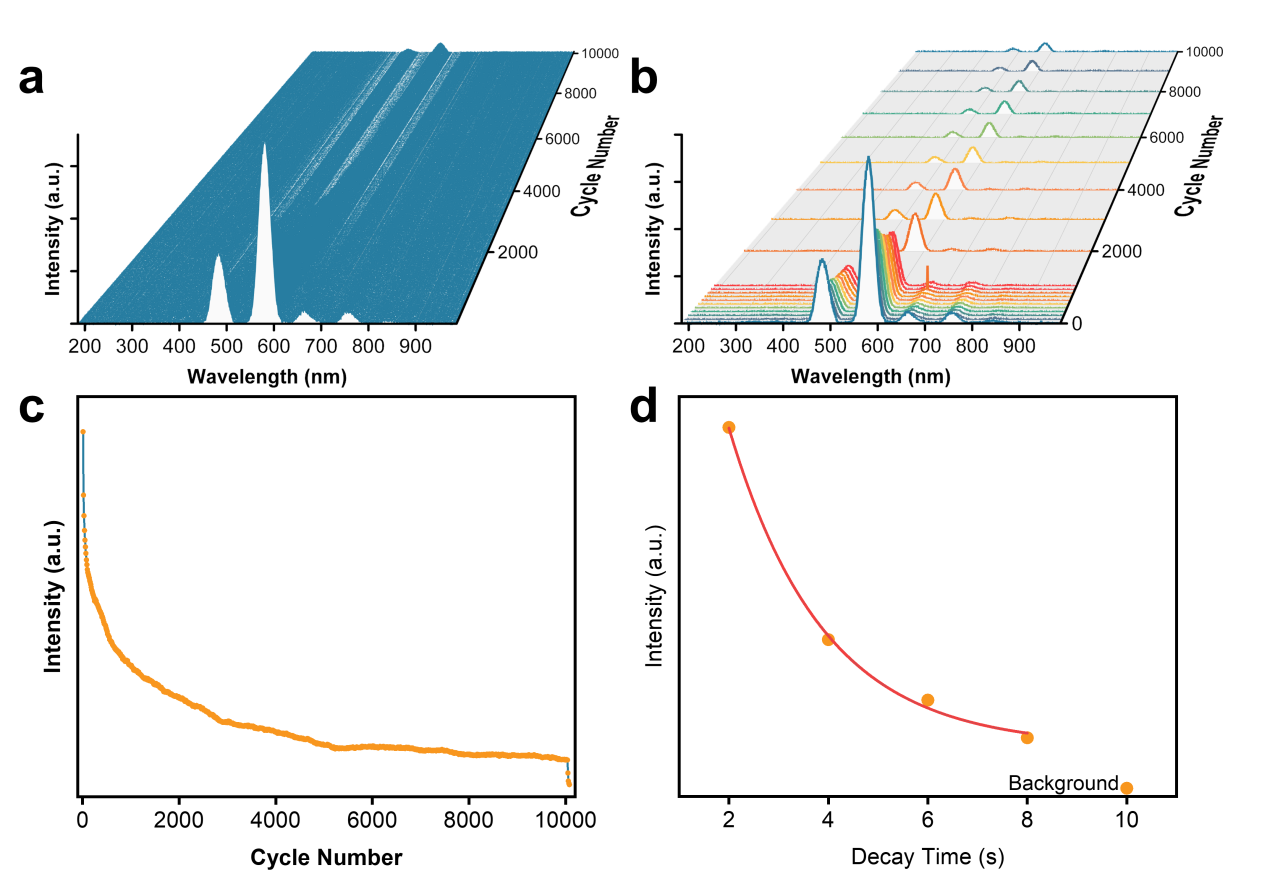


**Fig. S67 (a) (b)** The ML emission spectra and **(c)** the ML intensity evolution at 575 nm over 10,000 stretching cycles of the SBO:Dy/PDMS elastomer film under 40% stretching strain. **(d)** The ML persistent luminescence intensity evolution after stopping stretching.

**Table S1** ICP-MS results of the Sr_2.99_Pr_0.01_(BO_3_)_2_ phosphor.


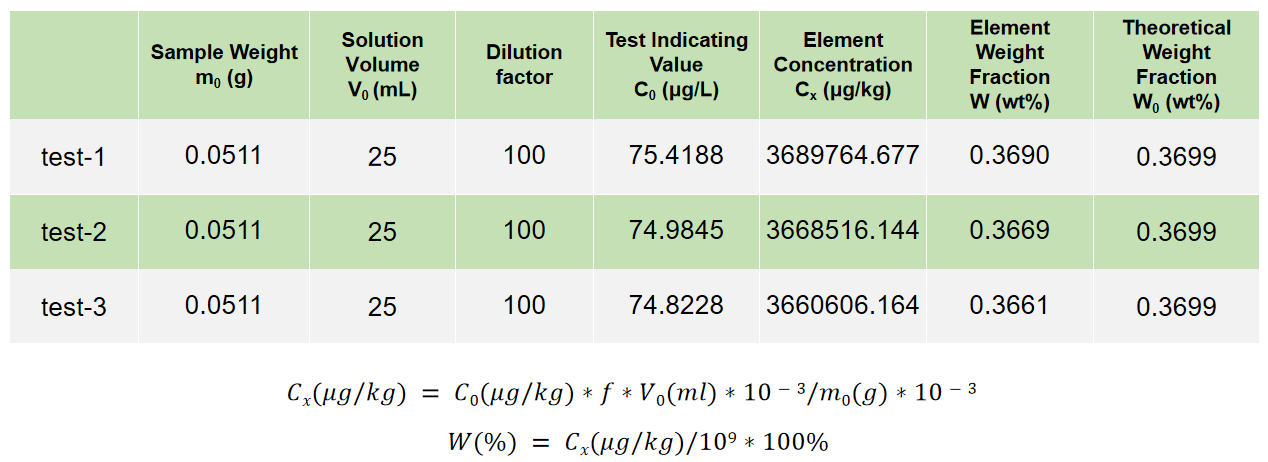


**Table S2** The strain and corresponding force and stress of the SBO:Pr/PDMS elastomer film under stretching stimulus.


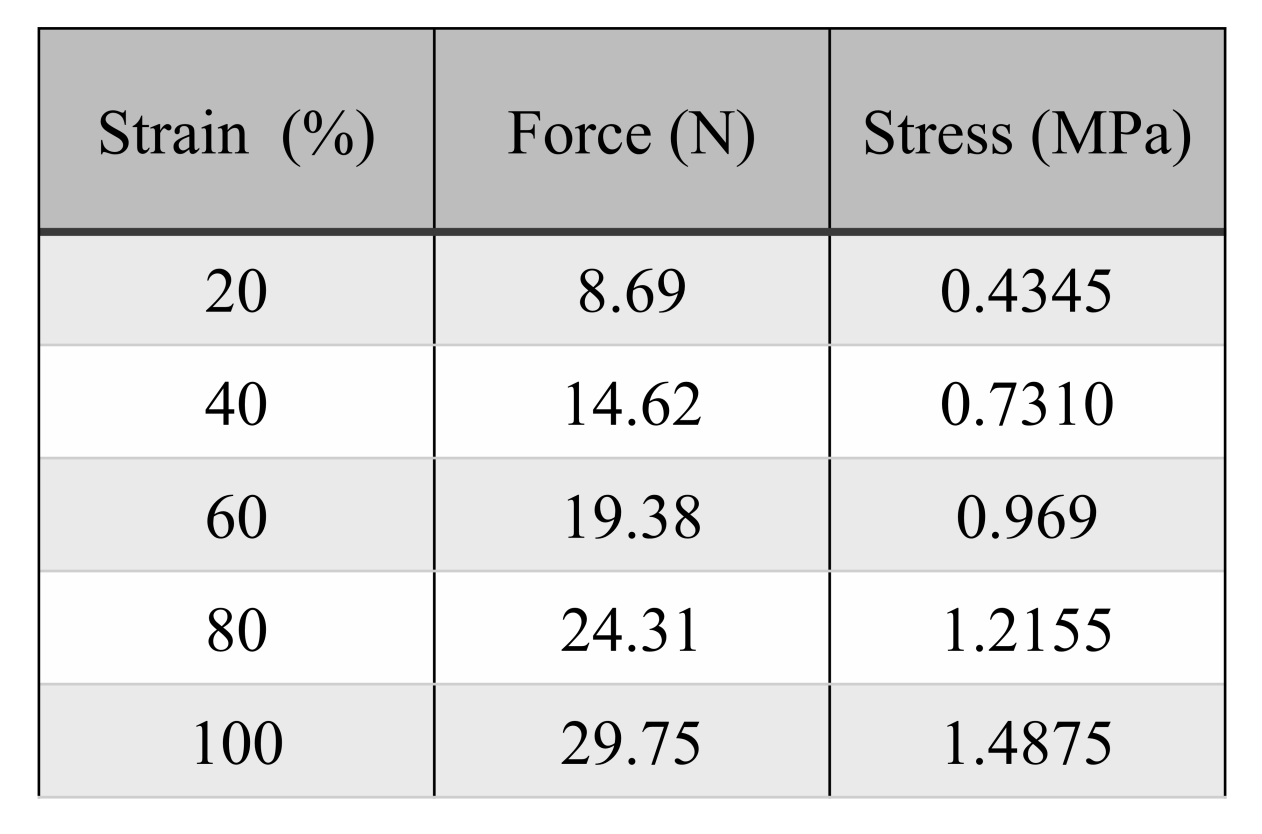

Supplement: Supplementary file 1 — Supporting Information [file 41377_2025_2131_MOESM1_ESM.docx]
